# Supplementary material for: Maize pan-transcriptome provides novel insights into genome complexity and quantitative trait variation
Source: Sci Rep. 2016 Jan 5;6:18936. doi: 10.1038/srep18936 (PMC4733048; doi:10.1038/srep18936)
Supplement: Supplementary Information [file srep18936-s1.pdf]

Supplemental Information for:

Maize pan-transcriptome provides novel insights into genome complexity and quantitative trait variation

Minliang Jin[1,†], Haijun Liu[1,†],Cheng He[2,3,†], Junjie Fu[3,*], Yingjie Xiao[1], Yuebin Wang[1], Weibo Xie[1], Guoying Wang[3] & Jianbing Yan[1,*]

[1]National Key Laboratory of Crop Genetic Improvement, Huazhong Agricultural University, Wuhan 430070, China

[2]College of Agriculture and Biotechnology, China Agricultural University, Beijing 100193, China

[3]Institute of Crop Science, Chinese Academy of Agricultural Sciences, Beijing 100081, China

[†]These authors contributed equally to this work.

[*]Correspondence should be addressed to Yan J (*yjianbing@mail.hzau.edu.cn*) and Fu J (*fujunjie@caas.cn*).

This file includes following supplementary figures and tables:

Supplementary Fig. S1. Comparison between the ePAV genes and common expression genes.
Supplementary Fig. S2. Comparison between our identified ePAV and genes identified as PAV in Mo17 inbred.
Supplementary Fig. S3. Validation of 10 randomly chosen ePAV genes which were predicted as PAV.
Supplementary Fig. S4. Length distribution of novel genes.
Supplementary Fig. S5. Length distribution of novel genes identified by two different assembly strategies.
Supplementary Fig. S6. A case for comparing the assembly reliability of two different strategies.
Supplementary Fig. S7. PCR-based validation of 10 randomly chosen novel genes.
Supplementary Fig. S8. Re-sequencing and reads distribution of 6 randomly selected novel genes.
Supplementary Fig. S9. Re-sequencing details of all referred novel genes.
Supplementary Fig. S10. Distribution of enzyme codes of annotated novel genes.
Supplementary Fig. S11. Pie chart of statistics on final annotation of novels.
Supplementary Fig. S12. GO enrichment analysis on novel genes in comparison with maize B73 reference.
Supplementary Fig. S13. A case to show the novel genes' mapping to reference based on LD mapping.
Supplementary Fig. S14. The chromosomes distribution of different kinds of variations.
Supplementary Fig. S15. Advantages of exploring maize genetics using ePAV.
Supplementary Fig. S16. Estimation of maize pan-transcriptome size.
Supplementary Fig. S17. Procedure of *de novo* assembly.

Supplementary Table S1. Detail information of enriched GO terms in ePAV and non-ePAV gene sets.
Supplementary Table S2. ePAV validation by genomic PCR of the previous identified 2M region (on chromosome 6) absence in MO17.
Supplementary Table S3. Premiers and materials used in genomic PCR validation for both ePAV candidates and novel genes.
Supplementary Table S4. The sequencing and assembly information for 368 inbred lines.
Supplementary Table S5. GO enrichment of novel genes compared to reference genome.
Supplementary Table S6. The predicted location of novel genes based on LD mapping to the reference genome.

Supplementary Figures and Figure Legends:

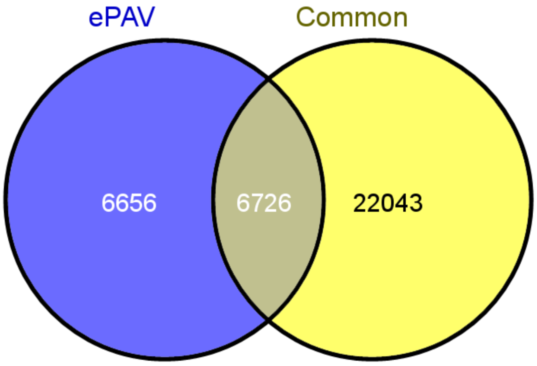

Supplementary Fig. S1. Comparison between the ePAV genes and common expression genes identified in a previous study (Fu et al. 2013).

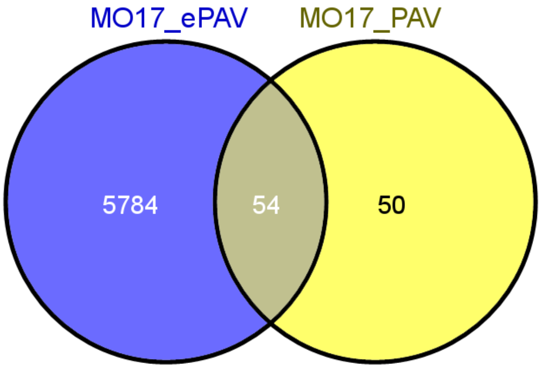

Supplementary Fig. S2. Comparison between our identified ePAV and genes identified as PAV (Lai et al. 2010) in Mo17 inbred.

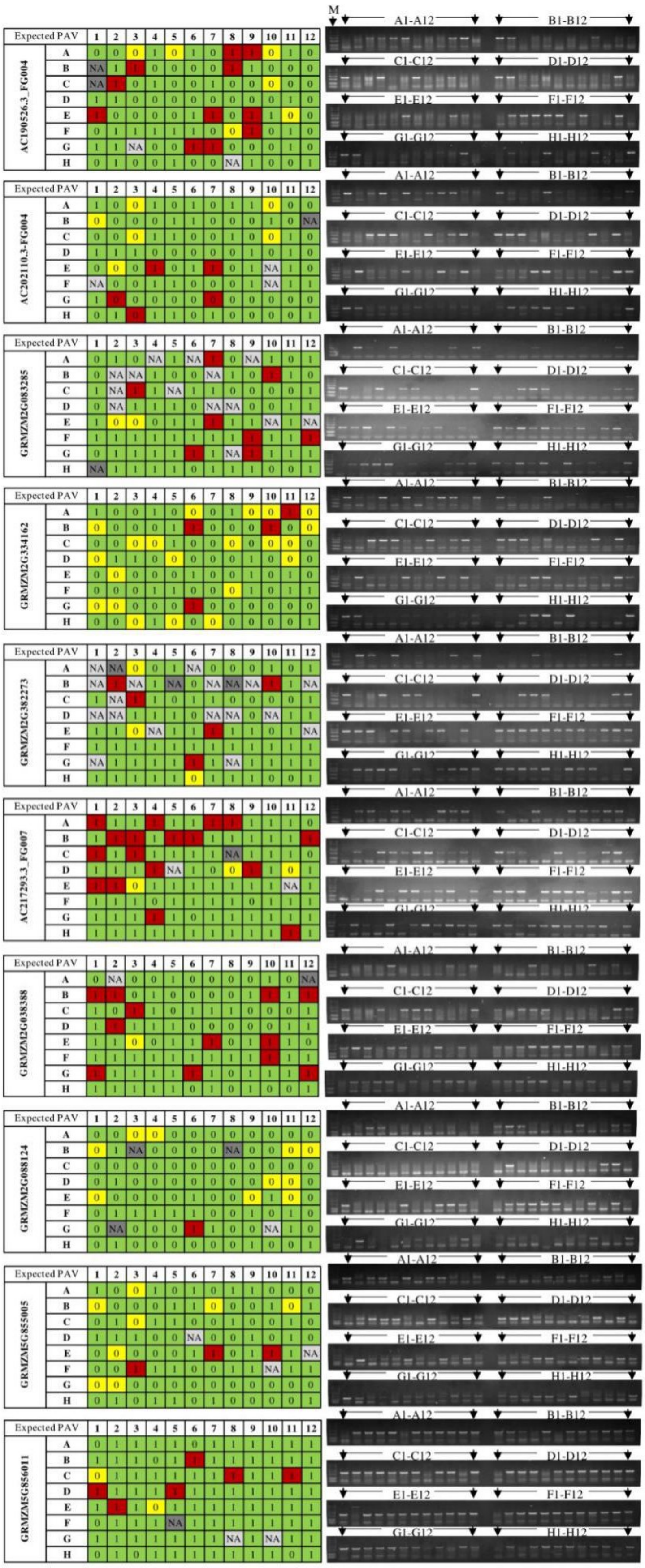

Supplementary Fig. S3. Validation of 10 randomly chosen ePAV genes that were predicted as PAV. Ten randomly chosen ePAV genes that were predicted as PAV were validated by genomic PCR using a set of 96 inbred lines. Left panel represents the correspondence between predicted ePAV and PCR results: green means completely consistent, red means present in expression but not validated as present by PCR, yellow represents absent in expression but present by PCR, and grey means expression not determined and absent in PCR (light) and present in PCR (dark).

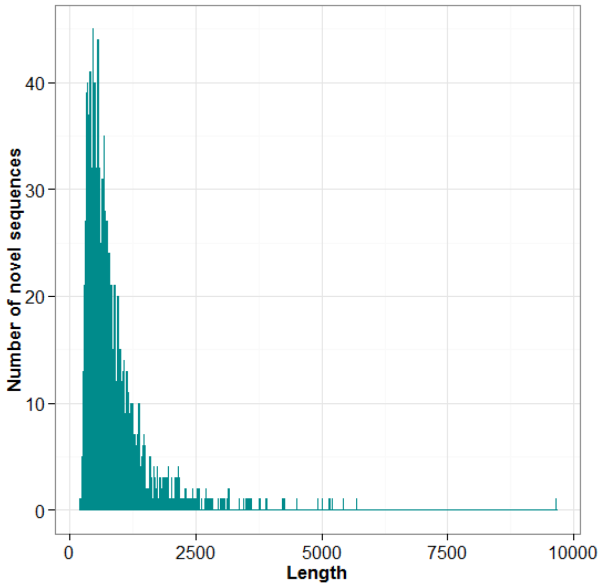

Supplementary Fig. S4. Length distribution of novel genes.

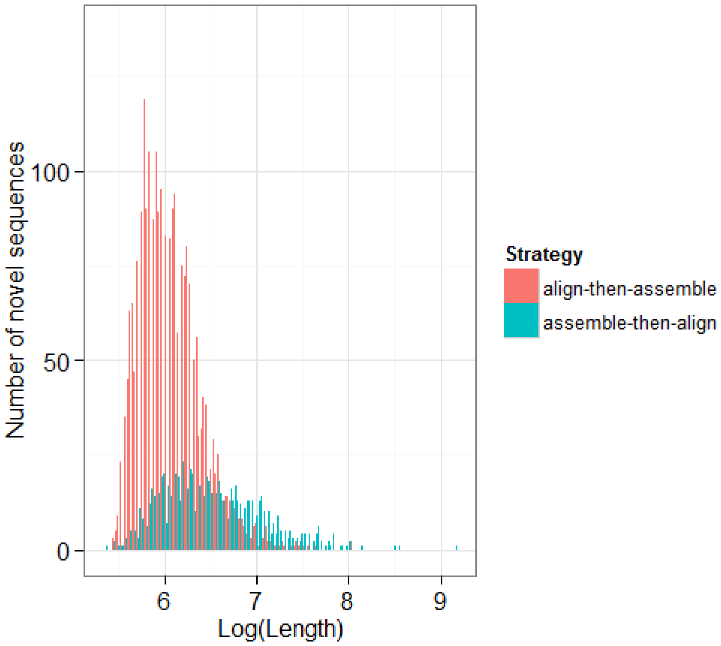

Supplementary Fig. S5. Length distribution of novel genes identified by two different assembly strategies.

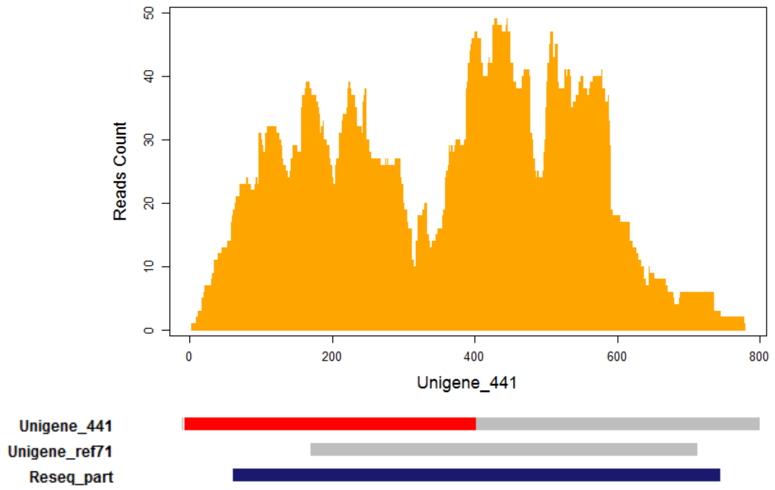

Supplementary Fig. S6. A case for comparing the assembly reliability of two different strategies.
Red represents the DUF789 domain and blue represents genes validated by resequencing, indicating that Unigene_ref71 was unable to completely predict the DUF789 domain.

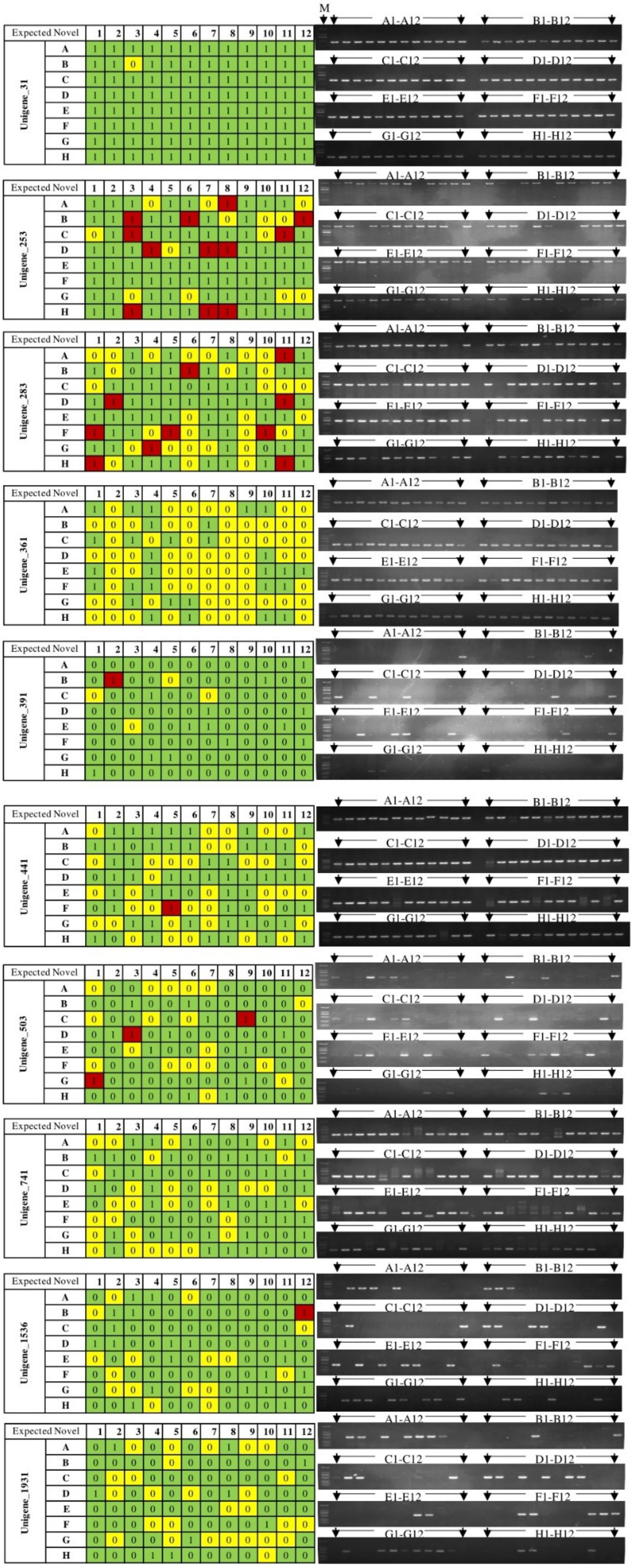

Supplementary Fig. S7. PCR-based validation of 10 randomly chosen novel genes.

Ten randomly chosen novel genes were validated by PCR using a set of 96 inbred lines. The left panel represents the correspondence of predicted presence or absence of novel genes and their PCR results. Green means completely consistent, red means present in expression but not validated as present by PCR, and yellow means absent in expression but present by PCR.

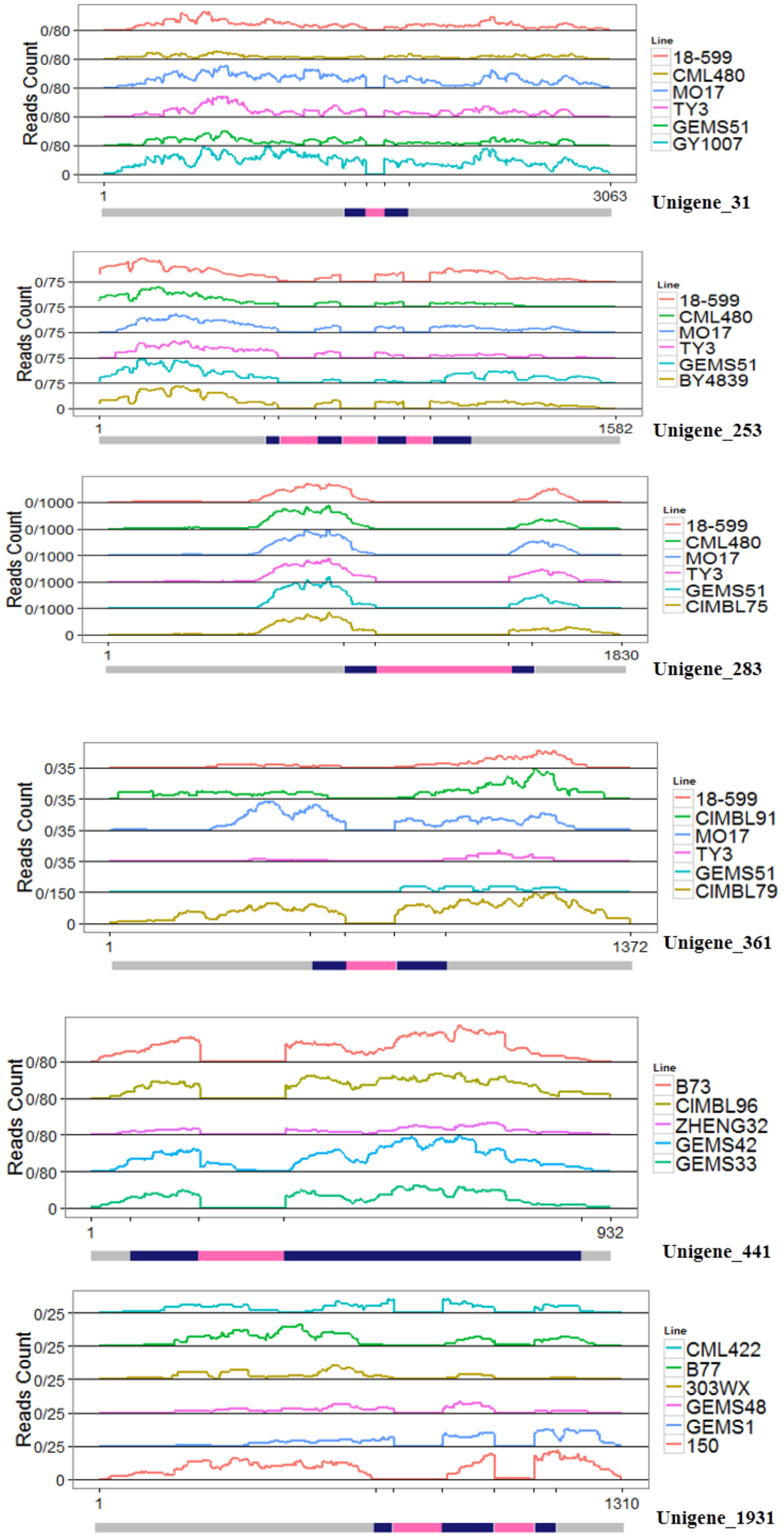

Supplementary Fig. S8. Re-sequencing and reads distribution of 6 randomly selected novel genes.
The blue segments were consistent with assembly sequences. The pink segments are most likely introns because there were no reads that could be mapped to these sections.

Supplementary Fig. S9. Re-sequencing details of all referred novel genes.

Unigene_ID [SeqStart-SeqEnd; Unigene length]. Pink segments indicate predicted introns.

Unigene_31 [1456-1728;2948]

```
Ref-Unigene_31  TATCCCCAGTATCCAACTTAAGTTCCCCGTAGTACATAAACTGAAGGAGAAGAAAGAATG
18-599          TATCCCCAGTATCCAACTTAAGTTCCCCGTAGTACATAAACTGAAGGAGAAGAAAGAATG
CML480          TATCCCCAGTATCCAACTTAAGTTCCCCGTAGTACATAAACTGAAGGAGAAGAAAGAATG
MO17            TATCCCCAGTATCCAACTTAAGTTCCCCGTAGTACATAAACTGAAGGAGAAGAAAGAATG
TY3             TATCCCCAGTATCCAACTTAAGTTCCCCGTAGTACATAAACTGAAGGAGAAGAAAGAATG
GEMS51          TATCCCCAGTATCCAACTTAAGTTCCCCGTAGTACATAAACTGAAGGAGAAGAAAGAATG
                ************************************************************

Ref-Unigene_31  GTTTAGGAGGAACATCTTCGAAAAAGATATCAGCGGCATTGCTTTCCTTCATCCCGTTTG
18-599          GTTTAGGAGGAACATCTTCGAAAAAGATATCAGCGGCATTGCTTTCCTTCATCCCGTTTG
CML480          GTTTAGGAGGAACATCTTCGAAAAAGATATCAGCGGCATTGCTTTCCTTCATCCCGTTTG
MO17            GTTTAGGAGGAACATCTTCGAAAAAGATATCAGCGGCATTGCTTTCCTTCATCCCGTTTG
TY3             GTTTAGGAGGAACATCTTCGAAAAAGATATCAGCGGCATTGCTTTCCTTCATCCCGTTTG
GEMS51          GTTTAGGAGGAACATCTTCGAAAAAGATATCAGCGGCATTGCTTTCCTTCATCCCGTTTG
                ************************************************************

Ref-Unigene_31  TGAACATCT---------------------------------------------------
18-599          TGAACATCTGCACAAATATTTGTGCCCATACTTTATTTATGAAAAAATGTGCATGATGCA
CML480          TGAACATCTGCACAAATATTTGTGCCCATACTTTATTTATGAAAAAATGTGCATGATGCA
MO17            TGAACATCTGCACAAATATTTGTGCCCATACTTTATTTATGAAAAAATGTGCATGATGCA
TY3             TGAACATCTGCACAAATATTTGTGCCCATACTTTATTTATGAAAAAATGTGCATGATGCA
GEMS51          TGAACATCTGCACAAATATTTGTGCCCATACTTTATTTATGAAAAAATGTGCATGATGCA
                *********

Ref-Unigene_31  ------------------------------------------------------------
18-599          TATTAAATAGTTATGCCAAAATTAGAATATGGCACTGGACACATAAGGGAAAATGTGTGT
CML480          TATTAAATAGTTATGCCAAAATTAGAATATGGTACTGGATACATAAGGGAAAATGTGTGT
MO17            TATTAAATAGTTATGCCAAAATTAGAATATGGCACTGGATACATAAGGGAAAATGTGTGT
TY3             TATTAAATAGTTATGCCAAAATTAGAATATGGCACTGGATACATAAGGGAAAATGTGTGT
GEMS51          TATTAAATAGTTATGCCAAAATTAGAATATGGCACTGGATACATAAGGGAAAATGTGTGT

Ref-Unigene_31  ----TGGCAAGAGGCACGCTCCACAGGCTAAGGATAAGCTTGTGACCCTTAGCGACAAGA
18-599          ACCTTGGCAAGAGGCACGCTCCACAGGCTAAGGATAAGCTTGTGACCCTTAGCGACAAGA
CML480          ACCTTGGCAAGAGGCACGCTCCACAGGCTAAGGATAAGCTTGTGACCCTTAGCGACAAGA
MO17            ACCTTGGCAAGAGGCACGCTCCACAGGCTAAGGATAAGCTTGTGACCCTTAGCGACAAGA
TY3             ACCTTGGCAAGAGGCACGCTCCACAGGCTAAGGATAAGCTTGTGACCCTTAGCGACAAGA
GEMS51          ACCTTGGCAAGAGGCACGCTCCACAGGCTAAGGATAAGCTTGTGACCCTTAGCGACAAGA
                    ********************************************************

Ref-Unigene_31  CCATGTCCATTAACATAGATATTTATATCACTATGTTCACCATTTGCAAGGAACTCTGCA
18-599          CCATGTCCATTAACATAGATATTTATATCACTATGTTCACCATTTGCAAGGAACTCTGCA
CML480          CCATGTCCATTAACATAGATATTTATATCACTATGTTCACCATTTGCAAGGAACTCTGCA
MO17            CCATGTCCATTAACATAGATATTTATATCACTATGTTCACCATTTGCAAGGAACTCTGCA
TY3             CCATGTCCATTAATATAGATATTTATATCACTATGTTCACCATTTGCAAGGAACTCTGCA
GEMS51          CCATGTCCATTAATATAGATATTTATATCACTATGTTCACCATTTGCAAGGAACTCTGCA
                ************* **********************************************

Ref-Unigene_31  ACTTTCTGCACATTCAGTGGCGCTTTGA
18-599          ACTTTCTGCACATTCAGTGGCGCTTTGA
CML480          ACTTTCTGCACATTCAGTGGCGCTTTGA
```

MO17 ACTTTCTGCACATTCAGTGGCGCTTTGA
TY3 ACTTTCTGCACATTCAGTGGCGCTTTGA
GEMS51 ACTTTCTGCACATTCAGTGGCGCTTTGA
****

Unigene_253 [506-829;1281]

Ref-Unigene_253 CTGCTCCGCCTGAGAAAGATGCCACAATGGGATCTACCTGG-------------------
18-599 CTGCTCCGCCTGAGAAAGATGCCACAATGGGATCTACCTGGCTGCAGTACAAGTAGACAA
CML480 CTGCTCCGCCTGAGAAAGATGCCACAATGGGATCTACCTGGCTGCAGAACAAGTAGACAA
MO17 CTGCTCCGCCTGAGAAAGATGCCACAATGGGATCTACCTGGCTGCAGTACAAGTAGACAA
TY3 CTGCTCCGCCTGAGAAAGATGCCACAATGGGATCTACCTGGCTGCAGTACAAGTAGACAA
GEMS51 CTGCTCCGCCTGAGAAAGATGCCACAATGGGATCTACCTGGCTGCAGTACAAGTAGACAA
****

Ref-Unigene_253 --
18-599 ATGAAGAGAGCAGTCAGTAGCCATAGCAGATCAGTCTTGACTTCTCTAGCTAGATGGTCC
CML480 ATGAAGAGAGCAGTCAGTAGCCATAGCAGATCAGTCTTGACTTCTCTAGCTAGATGGTCC
MO17 ATGAAGAGAGCAGTCAGTAGCCATAGCAGATCAGTCTTGACTTCTCTAGCTAGATGATCC
TY3 ATGAAGAGAGCAGTCAGTAGCCATAGCAGATCAGTCTTGACTTCTCTAGCTAGATGATCC
GEMS51 ATGAAGAGAGCAGTCAGTAGCCATAGCAGATCAGTCTTGACTTCTCTAGCTAGATGATCC

Ref-Unigene_253 ------------------------------------CTCAACTGCATCGGCAGATGAACG
18-599 TTGATACATTAATGGACCATGGAAAAGTGGTAGTACCTCAACTGCATCGGCAGATGAACG
CML480 TTGATACATTAATGGACCATGGAAAAGTGGTAGTACCTCAACTGCATCGGCAGATGAACG
MO17 TTGATACATTAATGGACCATGGAAAAGTGGTAGTACCTCAACTGCATCGGCAGATGAACG
TY3 TTGATACATTAATGGACCATGGAAAAGTGGTAGTACCTCAACTGCATCGGCAGATGAACG
GEMS51 TTGATACATTAATGGACCATGGAAAAGTGGTAGTACCTCAACTGCATCGGCAGATGAACG
****

Ref-Unigene_253 CTGCGGATGAAGTCTTCATAAGACGTGCCCCCTACACCAAGTTTAAGTTCCAG-------
18-599 CTGCGGATGAAGTCTTCATAAGACGTGCCCCCTACACCAAGTTTAAGTTCCAGCTGCATG
CML480 CTGCGGATGAAGTCTTCATAAGACGTGCCCCCTACACCAAGTTTAAGTTCCAGCTGCATG
MO17 CTGCGGATGAAGTCTTCATAAGACGTGCCCCCTACACCAAGTTTAAGTTCCAGCTGCATG
TY3 CTGCGGATGAAGTCTTCATAAGACGTGCCCCCTACACCAAGTTTAAGTTCCAGCTGCATG
GEMS51 CTGCGGATGAAGTCTTCATAAGACGTGCCCCCTACACCAAGTTTAAGTTCCAGCTGCATG
****

Ref-Unigene_253 --
18-599 ACATCACATCCACTTGTTAAGCATTCAAAGCAATTTATATGGCAAAATAAACTATGATAG
CML480 ACATCACATCCACTTGTTAAGCATTCAAAGCAATTTATATGGCAAAATAAACTATGATAG
MO17 ACATCACATCCACTTGTTAAGCATTCAAAGCAATTTATATGGCAAAATAAACTATGATAG
TY3 ACATCACATCCACTTGTTAAGCATTCAAAGCAATTTATATGGCAAAATAAACTATGATAG
GEMS51 ACATCACATCCACTTGTTAAGCATTCAAAGCAATTTATATGGCAAAATAAACTATGATAG

Ref-Unigene_253 --GATTGGTGCAAGAAAACCACC
18-599 TCACCATTAGCGATACCAAAATTGAAAACTGTGACTTACGATTGGTGCAAGAAAACCACC
CML480 TCACCATTAGCGATACCAAAATTGAAAACTGTGACTTACGATTGGTGCAAGAAAACCACC
MO17 TCACCATTAGCGATACCAAAATTGAAAACTGTGACTTACGATTGGTGCAAGAAAACCACC
TY3 TCACCATTAGCGATACCAAAATTGAAAACTGTGACTTACGATTGGTGCAAGAAAACCACC

GEMS51 TCACCATTAGCGATACCAAAATTGAAAACTGTGACTTACGATTGGTGCAAGAAAACCACC
****

Ref-Unigene_253 AAACACCATTATCCCAGCTATGAGGGTGAAACATGTGGCATAGTAGACCTTTAGATTAGC
18-599 AAACACCATTATCCCAGCTATGAGGGTGAAACATGTGGCATAGTAGACCTTTAGATTAGC
CML480 AAACACCATTATCCCAGCTATGAGGGTGAAACATGTGGCATAGTAGACCTTTAGATTAGC
MO17 AAACACCATTATCCCAGCTATGAGGGTGAAACATGTGGCATAGTAGACCTTTAGATTAGC
TY3 AAACACCATTATCCCAGCTATGAGGGTGAAACATGTGGCATAGTAGACCTTTAGATTAGC
GEMS51 AAACACCATTATCCCAGCTATGAGGGTGAAACATGTGGCATAGTAGACCTTTAGATTAGC
****

Ref-Unigene_253 TGCACTC---
18-599 TGCACTCTGTTCACATAAGGTAGAAGTTTATACCTTAGTAACAACTAGGCATCAAAGTGT
CML480 TGCACTCTGTTCACATAAGGTAGAAGTTTATACCTTAGTAACAACTAGGCATCAAAGTGT
MO17 TGCACTCTGTTCACATAAGGTAGAAGTTTATACCTTAGTAACAACTAGGCATCAAAGTGT
TY3 TGCACTCTGTTCACATAAGGTAGAAGTTTATACCTTAGTAACAACTAGGCATCAAAGTGT
GEMS51 TGCACTCTGTTCACATAAGGTAGAAGTTTATACCTTAGTAACAACTAGGCATCAAAGTGT
****

Ref-Unigene_253 -----------------------------AGAGGAGGCAAGAACGGAATAAATGATGGAA
18-599 CTGAGTGCTGAACGCCACACATACATACCAGAGGAGGCAAGAACGGAATAAATGATGGAA
CML480 CTGAGTGCTGAACGCCACACATACATACCAGAGGAGGCAAGAACGGAATAAATGATGGAA
MO17 CTGAGTGCTGAACGCCACACATACATACCAGAGGAGGCAAGAACGGAATAAATGATGGAA
TY3 CTGAGTGCCGAA--CCACACATACATACCAGAGGAGGCAAGAACGGAATAAATGATGGAA
GEMS51 CTGAGTGCTGAACGCCACACATACATACCAGAGGAGGCAAGAACGGAATAAATGATGGAA
****

Ref-Unigene_253 AATCAGGGAGTTCATGGTCACGCTCTTCATCTGAAATTCCGAGTTCCACATTTTTATTC
18-599 AATCAGGGAGTTCATGGTCACGCTCTTCATCTGAAATTCCGAGTTCCACATTTTTATTC
CML480 AATCAGGGAGTTCATGGTCACGCTCTTCATCTGAAATTCCGAGTTCCACATTTTTATTC
MO17 AATCAGGGAGTTCATGGTCACGCTCTTCATCTGAAATTCCGAGTTCCACATTTTTATTC
TY3 AATCAGGGAGTTCATGGTCACGCTCTTCATCTGAAATTCCGAGTTCCACATTTTTATTC
GEMS51 AATCAGGGAGTTCATGGTCACGCTCTTCATCTGAAATTCCGAGTTCCACATTTTTATTC
****

Ref-Unigene_253 TTTGCTGTATTCTCAATCTTCGAATCT
18-599 TTTGCTGTATTCTCAATCTTCGAATCT
CML480 TTTGCTGTATTCTCAATCTTCGAATCT
MO17 TTTGCTGTATTCTCAATCTTCGAATCT
TY3 TTTGCTGTATTCTCAATCTTCGAATCT
GEMS51 TTTGCTGTATTCTCAATCTTCGAATCT
****

Unigene_283 [839-1034;1355]

Ref-Unigene_283 GGAAGAGCACCCGAGGTCATCAAAACAGCCTAAAGAAAATAACAGGGATAGGAGGAGATC
18-599 GGAAGAGCACCCGAGGTCATCAAAACAGCCTAAAGAAAATAACAGGGATAGGAGGAGATC
CML480 GGAAGAGCACCCGAGGTCATCAAAACAGCCTAAAGAAAATAACAGGGATAGGAGGAGATC
MO17 GGAAGAGCACCCGAGGTCATCAAAACAGCCTAAAGAAAATAACAGGGATAGGAGGAGATC
TY3 GGAAGAGCACCCGAGGTCATCAAAACAGCCTAAAGAAAATAACAGGGATAGGAGGAGATC
GEMS51 GGAAGAGCACCCGAGGTCATCAAAACAGCCTAAAGAAAATAACAGGGATAGGAGGAGATC

```
                                          ************************************************************
Ref-Unigene_283 CTATACTCCTGATGATATAAATGATCGCCGTGGTGCAGACAATGGTCGTGATGAG-----
18-599          CTATACTCCTGATGATATAAATGATCGCCGAGGTGCAGACAATGGTCGTGATGAGTAAGT
CML480          CTATACTCCTGATGATATAAATGATCGCCGAGGTGCAGACAATGGTCGTGATGAGTAAGT
MO17            CTATACTCCTGATGATATAAATGATCGCCGTGGTGCAGACAATGGTCGTGATGAGTAAGT
TY3             CTATACTCCTGATGATATAAATGATCGCCGTGGTGCAGACAATGGTCGTGATGAGTAAGT
GEMS51          CTATACTCCTGATGATATAAATGATCGCCGTGGTGCAGACAATGGTCGTGATGAGTAAGT
                                          ****************************** ************************

Ref-Unigene_283 ------------------------------------------------------------
18-599          TCTGTTCCTTGACTGTTGGTACATGTCTATGGGTTAGTCTAGTTATGTTTTGAAGTAGTG
CML480          TCTGTTCCTTGACTGTTGGTACATGTCTATGGGTTAGTCTAGTTATGTTTTGAAGTAGTG
MO17            TCTGTTCCTTGACTGTTGGTACATGTCTATGGGTTAGTCTAGTTATGTTTTGAAGTAGTG
TY3             TCTGTTCCTTGACTGTTGGTACATGTCTATGGGTTAGTCTAGTTATGTTTTGAAGTAGTG
GEMS51          TCTGTTCCTTGACTGTTGGTACATGTCTATGGGTTAGTCTAGTTATGTTTTGAAGTAGTG

Ref-Unigene_283 ------------------------------------------------------------
18-599          ATATTGTGCATCCTATTGAGTGGTTATTTTTGTTGAGAAACATCCCATCTGTAACATGCA
CML480          ATATTGTGCATCCTATTGAGTGGTTATTTTTGTTGAGAAACATCCCATCTGTAACATGCA
MO17            ATATTGTGCATCCTATTGAGTGGTTATTTTTGTTGAGAAACATCCCATCTGTAACATGCA
TY3             ATATTGTGCATCCTATTGAGTGGTTATTTTTGTTGAGAAACATCCCATCTGTAACATGCA
GEMS51          ATATTGTGCATCCTATTGAGTGGTTATTTTTGTTGAGAAACATCCCATCTGTAACATGCA

Ref-Unigene_283 ------------------------------------------------------------
18-599          ACATTCATTTTGGGGTTGTTAAACTAAACATTTGTTTGACCAAGAAATGAGAAGTGGCCA
CML480          ACATTCATTTTGGGGTTGTTAAACTAAACATTTGTTTGACCAAGAAATGAGAAGTGGCCA
MO17            ACATTCATTTTGGGGTTGTTAAACTAAACATTTGTTTGACTAAGAAATGAGAAGTGGCCA
TY3             ACATTCATTTTGGGGTTGTTAAACTAAACATTTGTTTGACTAAGAAATGAGAAGTGGCCA
GEMS51          ACATTCATTTTGGGGTTGTTAAACTAAACATTTGTTTGACTAAGAAATGAGAAGTGGCCA

Ref-Unigene_283 ------------------------------------------------------------
18-599          CTTCTTTGTGGAGGACATCAGGGAATAGTGCCTTTTATTTGTTTGAGTAATTTTGTAAAG
CML480          CTTCTTTGTGGAGGACATCAGGGAATAGTGCCTTTTATTTGTTTGAGTAATTTTGTAAAG
MO17            CTTCTTTGTGGAGGACATCAGGGAATAGTGCCTTTTATTTGTTTGAGTAATTTTGTAAAG
TY3             CTTCTTTGTGGAGGACATCAGGGAATAGTGCCTTTTATTTGTTTGAGTAATTTTGTAAAG
GEMS51          CTTCTTTGTGGAGGACATCAGGGAATAGTGCCTTTTATTTGTTTGAGTAATTTTGTAAAG

Ref-Unigene_283 ------------------------------------------------------------
18-599          CTTGATGTGAAGTGCCTTTTCCCGAGCAATACTGCTGT-GGACATCCCTTGTACAGTTGC
CML480          CTTGATGTGAAGTGCCTTTTCCCGAGCAATACTGCTGT-GGACATCCCTTGTACAGTTGC
MO17            CTTGATGTGAAGTGCCTTTTCCCGAGCAATACTGCTGTTGGACATCCCTTGTACAGTTGC
TY3             CTTGATGTGAAGTGCCTTTTCCCGAGCAATACTGCTGTTGGACATCCCTTGTACAGTTGC
GEMS51          CTTGATGTGAAGTGCCTTTTCCCGAGCAATACTGCTGTTGGACATCCCTTGTACAGTTGC

Ref-Unigene_283 ------------------------------------------------------------
18-599          ATAATTAATGTATATTCAGGCACATTCTTTGCATTCTTTACAGGCGCGCTCGTTTTGGAT
```

```
CML480          ATAATTAATGTATATTCAGGCACATTCTTTGCATTCTTTACAGGCGCGCTCGTTTTGGAT
MO17            ACAATTAATGTATATTCAGGCACATTCTTTGCATTCTTTACAGGTGT-----------AT
TY3             ACAATTAATGTATATTCAGGCACATTCTTTGCATTCTTTACAGGTGT-----------AT
GEMS51          ACAATTAATGTATATTCAGGCACATTCTTTGCATTCTTTACAGGTGT-----------AT

Ref-Unigene_283 --------------------------------------------------------
18-599          TTTATTTTATATGGTAAAATAGAATTAATTATATATACACAGCAGGAGTAGCTGCTTCAG
CML480          TTTATTTTATATGGTAAAATAGAATTAATTATATATACACAGCAGGAGTAGCTGCTTCAG
MO17            TTTATTTTATATGGTAAAATAGAATTCATTATATATACACAGCAGGAGTAGCTGCTTCAG
TY3             TTTATTTTATATGGTAAAATAGAATTCATTATATATACACAGCAGGAGTAGCTGCTTCAG
GEMS51          TTTATTTTATATGGTAAAATAGAATTCATTATATATACACAGCAGGAGTAGCTGCTTCAG

Ref-Unigene_283 ------------------------------------------------AGGAAGAG
18-599          ATGGGTGTCTTTGTTGAATGGTAGTAACAGCTTGTTGTATGCTGATGACAGGAGGAAGAG
CML480          ATGGGTGTCTTTGTTGAATGGTAGTAACAGCTTGTTGTATGCTGATGACAGGAGGAAGAG
MO17            ATGGGTGTCTTTGTTGAATGGTAGTAACAGCTTGTTGTATGCTGATGACAGGAGGAAGAG
TY3             ATGGGTGTCTTTGTTGAATGGTAGTAACAGCTTGTTGTATGCTGATGACAGGAGGAAGAG
GEMS51          ATGGGTGTCTTTGTTGAATGGTAGTAACAGCTTGTTGTATGCTGATGACAGGAGGAAGAG
                                                                    ********

Ref-Unigene_283 GTCCCCAGCAGGCGAGGAGGATGAGGAGCCTCGGCGCGGTCATCATAGGTCACCCCGTCC
18-599          ATCCCCAGCAGGCGAGGAGGATGAGGAGCCTCGGCGCGGTCATCATAGGTCACCCCGGCC
CML480          ATCCCCAGCAGGCGAGGAGGATGAGGAGCCTCGGCGCGGTCATCATAGGTCACCCCGGCC
MO17            GTCCCCAGCAGGCGAGGAGGATGAGGAGCCTCGGCGCGGTCATCATAGGTCACCCCGGCC
TY3             GTCCCCAGCAGGCGAGGAGGATGAGGAGCCTCGGCGCGGTCATCATAGGTCACCCCGGCC
GEMS51          GTCCCCAGCAGGCGAGGAGGATGAGGAGCCTCGGCGCGGTCATCATAGGTCACCCCGGCC
                 ******************************************************** ***

Ref-Unigene_283 AGCGTCCATGTCA
18-599          AGCTTCCATGTCA
CML480          AGCTTCCATGTCA
MO17            AGCGTCCATGTCA
TY3             AGCGTCCATGTCA
GEMS51          AGCGTCCATGTCA
                *** *********
```

Unigene_361 [529-751;1240]

```
Ref-Unigene_361 CCTCGTCGCCACCGTCAACTGCACCGGCGCCCTGTTCCAGCT-CGCCTACATCTCGCTCT
18-599          CCTCGTCGCCACCGTCAACTGCACCGGCGCCCTGTTCCAGCT-CGCCTACATCTCGCTCT
MO17            CCTCGTCGCCACCGTCAACTGCACCGGCGCCCTGTTCCAGCT-CGCCTACATCTCGCTCT
CML480          CCTCGTCGCCACCGTCAACTGCACCGGCGCCCTGTTCCAGCT-CGCCTACATCTCGCTCT
TY3             CCTCGTCGCCACCGTCAACTGCACCGGCGCCCTGTTCCAGCT-CGCCTACATCTCGCTCT
GEMS51          CCTCGTCGCCACCGTCAACTGCACCGGCGCCCTGTTCCAGCTTCGCCTACATCTCGCTCT
                ****************************************** *****************

Ref-Unigene_361 TCATCTTCTACGCCGACAGCAGGACCACTCGG----------------------------
18-599          TCATCTTCTACGCCGACAGCAGGACCACTCGGGTAAGTAAATTCATGTGCTTGCCGTGGA
MO17            TCATCTTCTACGCCGACAGCAGGACCACTCGGGTAAGTAAATTCATGTGCTTGCC-----
```

CML480 TCATCTTCTACGCCGACAGCAGGACCACCCGGGTAAGTAAATTCATGTGCTTGCC-----
TY3 TCATCTTCTACGCCGACAGCAGGACCACTCGGGTAAGTAAATTCATGTGCTTGCCGTGGA
GEMS51 TCATCTTCTACGCCGACAGCAGGACCACTCGGGTAAGTAAATTCATGTGCTTGCC-----
**************************** ***

Ref-Unigene_361 --
18-599 ATCGTGTTGCCTTGCCTGCTCTCTTCTCTTGCCGTGTCTGTCTGACC-GACCGACTGACA
MO17 -----------TTGCCTGCCCTCTTCTCTTGCCGTGTCTGTCTGACC-GACCGACTGACA
CML480 ------------------GTGGAATCGTGTTGCCTTGCCTGCCCTCTTGCCGTGTCTGT
TY3 ATCGTGTTGCCTTGCCTGCCCTCTTCTCTTGCCGTGTCTGTCTGACCAGACCGACTGACA
GEMS51 -----------TTGCCTGCCCTCTTCTCTTGCCGTGTCTGTCTGACC-GACCGACTGACA

Ref-Unigene_361 --CTCAAGGTCGCGG
18-599 CCAGTCTGCTCTGCTCTTCTCCATCTCCTCCCGTCCTCGATCCGCAGCTCAAGGTCGCGG
MO17 CCAGTCTGCTCTGCTCT-CTCCATCTCCTCCCGTCCTCGATCCGCAGCTCAAGGTCGCGG
CML480 CTGACCGACTGACACCACTAAAATTCATCTCCTCCGGTCCTCCGCAGCTCAAGGTCGCGG
TY3 CCAGTCTGCTCTGCTCT---CCATCTCCTCCCGTCCTCCATCCGCAGCTCAAGGTCGCGG
GEMS51 CCAGTCTGCTCTGCTCT-CTCCATCTCCTCCCGTCCTCGATCCGCAGCTCAAGGTCGCGG
****

Ref-Unigene_361 GGCTTCTGGTGCTAGTGGTCTTCGCGTTCGCGCTCATCGCACACGCCAGCATCGCCTTCT
18-599 GGCTTCTGGTGCTAGTGGTCTTCGCGTTCGCCCTCATCGCACACGCCAGCATCGCCTTCT
MO17 GGCTTCTGGTGCTAGTGGTCTTCGCGTTCGCGCTCATCGCACACGCCAGCATCGCCTTCT
CML480 GGCTTCTGCTGCTAGTCGTCTTCGCGTTCGCGCTCATCGCACACGCCAGCATCGCCTTCT
TY3 GGCTTCTGGTGCTAGTGGTCTTCGCGTTCGCGCTCATCGCACACGCCAACATCGCCTTCT
GEMS51 GGCTTCTGGTGCTAGTGGTCTTCGCGTTCGCGCTCATCGCACACGCCAGCATCGCCTTCT
******** ******* ************** **************** ***********

Ref-Unigene_361 TCGACCAGCCGCTCCGGCAGCTGTTCGTTGGCAGCGTCAGCATGGCGTCCCTGGTCTCC
18-599 TCGACCAGCCGCTCCGGCAGCTGTTCGTTGGCAGCGTCAGCATGGCGTCCCTGGTCTCC
MO17 TCGACCAGCCGCTCCGGCAGCTGTTCGTTGGCAGCGTCAGCATGGCGTCCCTGGTCTCC
CML480 TCGACCAGCCGCTCCGCCAGCTGTTCGTTGGCAGCGTCAGCATGGCGTCCCTGGTCTCC
TY3 TCGACCAGCCGCTCCGGCAGCTGTTCGTTGGCAGCGTCAGCATGGCGTCCCTGGTCTCC
GEMS51 TCGACCAGCCGCTCCGGCAGCTGTTCGTTGGCAGCGTCAGCATGGCGTCCCTGGTCTCC
**************** ***

Unigene_391 [282-538;1894]

Ref-Unigene_391 AGTTTTCTTCCTTTACAGCCTTTGAAACTTATGGCCCTTGCCCTGGTGAGGAAACTTTTT
GEMS51 AGTTTTCTTCCTTTACAGCCTTTGAA-CTTATGGCCCTTGCCCTGGTGAGGAAACTTTTT
R15X1141 AGTTTTCTTCCTTTACAGCCTTTGAA-CTTATGGCCCTTGCCCTGGTGAGGAAACTTTTT
GEMS48 AGTTTTCTTCCTTTACAGCCTTTGAAACTTATGGCCCTTGCCCTGGTGAGGAAACTTTTT
BY809 AGTTTTCTTCCTTTACAGCCTTTGAA-CTTATGGCCCTTGCCCTGGTGAGGAAACTTTTT
CIMBL63 AGTTTTCTTCCTTTACAGCCTTTGAA-CTTATGGCCCTTGCCCTGGTGAGGAAACTTTTT
************************** **********************************

Ref-Unigene_391 GAAATTGTTGAAGCTTCATTGCATTTTAACAATAGGGCATACCGGAACGTTACTTTTTTT
GEMS51 GAAATTGTTGAAGCTTCATTGCATTTTAACAATAGGGCATACCGGAACGTTACTTTTTTT
R15X1141 GAAATTGTTGAAGCTTCATTGCATTTTAACAATAGGGCATACCGGAACGTTACTTTTTTT
GEMS48 GAAATTGTTGAAGCTTCATTGCATTTTAACAATAGGGCATACCGGAACGTTACTTTTTTT
BY809 GAAATTGTTGAAGCTTCATTGCATTTTAACAATAGGGCATACCGGAACGTTACTTTTTTT

```
CIMBL63          GAAATTGTTGAAGCTTCATTGCATTTTAACAATAGGGCATACCGGAACGTTACTTTTTT
                 ************************************************************

Ref-Unigene_391  TTGTTCCTAACAGGTGCTATTTGATCTTCCAAAATTATGAGCTCATCACACGATAGATAT
GEMS51           TTGTTCCTAACAGGTGCTATTTGATCTTCCAAAATTATGAGCTCATCACACGATAGATAT
R15X1141         TTGTTCCTAACAGGTGCTATTTGATCTTCCAAAATTATGAGCTCATCACACGATAGATAT
GEMS48           TTGTTCCTAACAGGTGCTATTTGATCTTCCAAAATTATGAGCTCATCACACGATAGATAT
BY809            TTGTTCCTAACAGGTGCTATTTGATCTTCCAAAATTATGAGCTCATCACACGATAGATAT
CIMBL63          TTGTTCCTAACAGGTGCTATTTGATCTTCCAAAATTATGAGCTCATCACACGATAGATAT
                 ************************************************************

Ref-Unigene_391  CTGGCAAAGCTATGCACCAAGCTGTGCATTTCGAGCCCATTTTTACCATTGGTGAGAACC
GEMS51           CTGGCAAAGCTATGCACCAAGCTGTGCATTTCGAGCCCATTTTTACCATTGGTGAGAACC
R15X1141         CTGGCAAAGCTATGCACCAAGCTGTGCATTTCGAGCCCATTTTTACCATTGGTGAGAACC
GEMS48           CTGGCAAAGCTATGCACCAAGCTGTGCATTTCGAGCCCATTTTTACCATTGGTGAGAACC
BY809            CTGGCAAAGCTATGCACCAAGCTGTGCATTTCGAGCCCATTTTTACCATTGGTGAGAACC
CIMBL63          CTGGCAAAGCTATGCACCAAGCTGTGCATTTCGAGCCCATTTTTACCATTGGTGAGAACC
                 ************************************************************

Ref-Unigene_391  ATACCATTCGCCGAGCA
GEMS51           ATACCATTCGCCGAGC-
R15X1141         ATACCATTCGCCGAGCA
GEMS48           ATACCATTCGCCGAGCA
BY809            ATACCATCCGCCGAGCA
CIMBL63          ATACCATCCGCCGAGCA
                 ******* ********
```

Unigene_441 [70-725;778]

```
Ref-Unigene_441  GCTTCTTTCCTGACATACCACTCCCTAGGAAAATTGGTTCCACAAACTTGCTCTACGGAT
B73              GCTTCTTTCCTGACATACCACTCCCTAGGAAAATTGGTTCCACAAACTTGCTCTACGGAT
CIMBL96          GCTTCTTTCCTGACATACCACTCCCTAGGAAAATTGGTTCCACAAACTTGCTCTACGGAT
ZHENG32          GCTTCTTTCCTGACATACCACTCCCTAGGAAAATTGGTTCCACAAACTTGCTCTACGGAT
GEMS42           GCTTCTTTCCTGACATACCACTCCCTAGGAAAATTGGTTCCACAAACTTGCTCTACGGAT
                 ************************************************************

Ref-Unigene_441  AATAAGGCTGATGCGTATGATCGTATGATCTGTCCGATTGTCGGTCTGCTTGCCTACAAA
B73              AATAAGGCTGATGCGTATGATCGTATGATCTGTCCGATTGTCGGTCTGCTTGCCTACAAA
CIMBL96          AATAAGGCTGATGCGTATGATCGTATGATCTGTCCGATTGTCGGTCTGCTTGCCTACAAA
ZHENG32          AATAAGGCTGATGCGTATGATCGTATGATCTGTCCGATTGTCGGTCTGCTTGCCTACAAA
GEMS42           AATAAGGCTGATGCGTATGATCGTATGATCTGTCCGATTGTCGGTCTGCTTGCCTACAAA
                 ************************************************************

Ref-Unigene_441  GACCAGG-----------------------------------------------------
B73              GACCAGGTAGTGGTTCAACTGACTACACTTCAGTACTACTAGACAGAAATATGTATATGC
CIMBL96          GACCAGGTAGTGGTTCAACTGACTACACTTCAGTACTACTAGACAGAAATATGTATATGC
ZHENG32          GACCAGGTAGTGGTTCAACTGACTACACTTCAGTACTACTAGACAGAAATATGTATATGC
GEMS42           GACCAGGTAGTGGTTCAACTGACTACACTTCAGTACTACTAGACAGAAATATGTATATGC
                 *******

Ref-Unigene_441  ------------------------------------------------------------
B73              ATATCTATATTTCTATTTCTATTTCTCTTTTAAAAAGAACTATATGTACCTGTTCTTTAT
CIMBL96          ATATCTATATTTCTATTTCTATTTCTCTTTTAAAAAGAACTATATGTACCTGTTCTTTAT
```

```
ZHENG32          ATATCTATATTTCTATTTCTATTTCTCTTTTAAAAAGAACTATATGTACCTGTTCTTTAT
GEMS42           ATATCTATATTTCTATTTCTATTTCTCTTTTAAAAAGAACTATATGTACCTGTTCTTTAT

Ref-Unigene_441  -------------------------------------GAGAGAAGTGGTTTCAACTGAGA
B73              TGGTGTTATTTGTTATGAAAAATTTCTTATTTACAGGGAGAGAAGTGGTTTCAACTGAGA
CIMBL96          TGGTGTTATTTGTTATGAAAAATTTCTTATTTACAGGGAGAGAAGTGGTTTCAACTGAGA
ZHENG32          TGGTGTTATTTGTTATGAAAAATTTCTTATTTACAGGGAGAGAAGTGGTTTCAACTGAGA
GEMS42           TGGTGTTATTTGTTATGAAAAATTTCTTATTTACAGGGAGAGAAGTGGTTTCAACTGAGA
                                                      ***********************

Ref-Unigene_441  GAACAACAATTCAGGATCAAACCAAGGCCAAACGTTTCCTCGGGAACAGACCATGCTGAA
B73              GAACAACAACTCAGGATCAAACCAAGGCCAAACGTTTCCTCGGGAACAGACCATGCTGAA
CIMBL96          GAACAACAACTCAGGATCAAACCAAGGCCAAACGTTTCCTCGGGAACAGACCATGCTGAA
ZHENG32          GAACAACAACTCAGGATCAAACCAAGGCCAAACGTTTCCTCGGGAACAGACCATGCTGAA
GEMS42           GAACAACAATTCAGGATCAAACCAAGGCCAAACGTTTCCTCGGGAACAGACCATGCTGAA
                 ********* **************************************************

Ref-Unigene_441  CTCCTGAACAAGAGGCTGAGGGCACTGAGGCAATGTGCATCAATCATGTCGAAGGCAGTG
B73              CTCCTGAACAAGAGGCTGAGGGCACTGAGGCAATGTGCATCAATCATGTCGAAGGCAGTG
CIMBL96          CTCCTGAACAAGAGGCTGAGGGCACTGAGGCAATGTGCATCAATCATGTCGAAGGCAGTG
ZHENG32          CTCCTGAACAAGAGGCTGAGGGCACTGAGGCAATGTGCATCAATCATGTCGAAGGCAGTG
GEMS42           CTCCTGAACAAGAGGCTGAGGGCACTGAGGCAATGTGCATCAATCATGTCGAAGGCAGTG
                 ************************************************************

Ref-Unigene_441  GTGCCAAGGGCGCCATCTGGAGAAGCCATGAACTGTCATTCAGATTACGAGTTCTTTTTG
B73              GTGCCAAGGGCGCCATCTGGAGAAGCCATGAACTATCATTCAGATTACGAGTTCTTTTTG
CIMBL96          GTGCCAAGGGCGCCATCTGGAGAAGCCATGAACTATCATTCAGATTACGAGTTCTTTTTG
ZHENG32          GTGCCAAGGGCGCCATCTGGAGAAGCCATGAACTATCATTCAGATTACGAGTTCTTTTTG
GEMS42           GTGCCAAGGGCGCCATCTGGAGAAGCCATGAACTGTCATTCAGATTACGAGTTCTTTTTG
                 ********************************** *************************

Ref-Unigene_441  TCAAGGTCGTCTCTGCAGTAAGCATGCGCGTTATGTTATTGCATCATATCTACCGATGCG
B73              TCAAGGTCGTCTCTGCAGTAAGCATGCGCGTTATGT-ATTGCATCATATCTACCGATGCG
CIMBL96          TCAAGGTCGTCTCTGCAGTAAGCATGCGCGTTATGT-ATTGCATCATATCTACCGATGCG
ZHENG32          TCAAGGTCGTCTCTGCAGTAAGCATGCGCGTTATGT-ATTGCATCATATCTACCGATGCG
GEMS42           TCAAGGTCGTCTCTGCAGTAAGCATGCGCGTTATGT-ATTGCATCATATCTACCGATGCG
                 ************************************ ***********************

Ref-Unigene_441  CGCGGTGTTTCGAATGGGCTGATCTAGTCCATTCAGTACCTGCTCCAGCGTCTCCATACT
B73              CGCGGTGTTTCGAATGGGCTGATCTAGTCCATTCAGTACCTGCTCCAGCGTCTCCATACT
CIMBL96          CGCGGTGTTTCGAATGGGCTGATCTAGTCCATTCAGTACCTGCTCCAGCGTCTCCATACT
ZHENG32          CGCGGTGTTTCGAATGGGCTGATCTAGTCCATTCAGTACCTGCTCCAGCGTCTCCATACT
GEMS42           CGCGGTGTTTCGAATGGGCTGATCTAGTCCATTCAGTACCTGCTCCAGCGTCTCCATACT
                 ************************************************************

Ref-Unigene_441  AATATAATCTCTCCCCTTGCATGAGCATGACCAACTTAAGTTTCGCATGCATTGGGGCTG
B73              AATATAATCTCTCCCCTTGCATGAGCATGACCAACTTAAGTTTCGCATGCATTGGGGCTG
CIMBL96          AATATAATCTCTCCCCTTGCATGAGCATGACCAACTTAAGTTTCGCATGCATTGGGGCTG
ZHENG32          AATATAATCTCTCCCCTTGCATGAGCATGACCAACTTAAGTTTCGCATGCATTGGGGCTG
```

GEMS42 AATATAATCTCTCCCCTTGCATGAGCATGACCAACTTAAGTTTCGCATGCATTGGGGCTG
****

Ref-Unigene_441 ACGCGAGGCTGCTTCAAACAAGCTGTTGAGTTTGTGTTTTTGTTTCCCTTGGTACTAGTA
B73 ACGCGAGGCTGCTTCAAACAAGCTGTTGAGTTTGTGTTTTTGTTTCCCTTGGTACTAGTA
CIMBL96 ACGCGAGGCTGCTTCAAACAAGCTGTTGAGTTTGTGTTTTTGTTTCCCTTGGTACTAGTA
ZHENG32 ACGCGAGGCTGCTTCAAACAAGCTGTTGAGTTTGTGTTTTTGTTTCCCTTGGTACTAGTA
GEMS42 ACGCGAGGCTGCTTCAAACAAGCTGTTGAGTTTGTGTTTTTGTTTCCCTTGGTACTAGTA
****

Ref-Unigene_441 CAATACCCAGATGACATGGGGTTTAAGTTGGATGTTCTATATATGTTCTAAAGCCTTGAC
B73 CAATACCCAGATGACATGGGGTTTAAGTTGGATGTTCTATATATGTTCTAAAGCCTTGAC
CIMBL96 CAATACCCAGATGACATGGGGTTTAAGTTGGATGTTCTATATATGTTCTAAAGCCTTGAC
ZHENG32 CAATACCCAGATGACATGGGGTTTAAGTTGGATGTTCTATATATGTTCTAAAGCCTTGAC
GEMS42 CAATACCCAGATGACATGGGGTTTAAGTTGGATGTTCTATATATGTTCTAAAGCCTTGAC
****

Ref-Unigene_441 ATATATTGCTGTTGGCTTGTGGATCC
B73 ATATATTGCTGTTGGCTTGTGGATCC
CIMBL96 ATATATTGCTGTTGGCTTGTGGATCC
ZHENG32 ATATATTGCTGTTGGCTTGTGGATCC
GEMS42 ATATATTGCTGTTGGCTTGTGGATCC
****

Unigene_503 [455-778;1337]

Ref-Unigene_503 AGACGCACCATGGCCACTACCACTTCAAGGTTATGCCGTTCGGCCTCTCAAACGCCCCAG
303WX AGACGCACCATGGCCACTACCACTTCAAGGTTATGCCGTTCGGCCTCTCAAACGCCCCAG
GEMS1 AGACGCACCATGGCCACTACCACTTCAAGGTTATGCCGTTCGGCCTCTCAAACGCCCCAG
GEMS51 AGACGCACCATGGCCACTACCACTTCAAGGTTATGCCGTTCGGCCTCTCAAACGCCCCAG
CML422 AGACGCACCATGGCCACTACCACTTCAAGGTTATGCCGTTCGGCCTCTCAAACGCCCCAG
BY809 AGACGCACCATGGCCACTACCACTTCAAGGTTATGCCGTTCGGCCTCTCAAACGCCCCAG
****

Ref-Unigene_503 CAACATTTCAATGCATTATGAATGATATCTTGAGTCCCTTTCTGCGCAAGTTTGTCTTGG
303WX CAACATTTCAATGCATTATGAATGATATCTTGAGTCCCTTTCTGCGCAAGTTTGTCTTGG
GEMS1 CAACATTTCAATGCATTATGAATGATATCTTGAGTCCCTTTCTGCGCAAGTTTGTCTTGG
GEMS51 CAACATTTCAATGCATTATGAATGATATCTTGAGTCCCTTTCTGCGCAAGTTTGTCTTGG
CML422 CAACATTTCAATGCATTATGAATGATATCTTGAGTCCCTTTCTGCGCAAGTTTGTCTTGG
BY809 CAACATTTCAATGCATTATGAATGATATCTTGAGTCCCTTTCTGCGCAAGTTTGTCTTGG
****

Ref-Unigene_503 TGTTTATGGACGACATACTTGTTTACAGTCCCACCTTAGAGCTGCATGTCCAACACTTGC
303WX TGTTTATGGACGACATACTTGTTTACAGTCCCACCTTAGAGCTGCATGTCCAACACTTGC
GEMS1 TGTTTATGGACGACATACTTGTTTACAGTCCCACCTTAGAGCTGCATGTCCAACACTTGC
GEMS51 TGTTTATGGACGACATACTTGTTTACAGTCCCACCTTAGAGCTGCATGTCCAACACTTGC
CML422 TGTTTATGGACGACATACTTGTTTACAGTCCCACCTTAGAGCTGCATGTCCAACACTTGC
BY809 TGTTTATGGACGACATACTTGTTTACAGTCCCACCTTAGAGCTGCATGTCCAACACTTGC
****

Ref-Unigene_503 AACAAGTGTTCAAGCAGCTCAGGTTGCACCAGTTTTTTCTCAAGTTGTCAAAATGCCAGT
303WX AACAAGTGTTCAAGCAGCTCAGGTTGCACCAGTTTTTTCTCAAGTTGTCAAAATGCCAGT
GEMS1 AACAAGTGTTCAAGCAGCTCAGGTTGCACCAGTTTTTTCTCAAGTTGTCAAAATGCCAGT

GEMS51 AACAAGTGTTCAAGCAGCTCAGGTTGCACCAGTTTTTCTCAAGTTGTCAAAATGCCAGT
CML422 AACAAGTGTTCAAGCAGCTCAGGTTGCACCAGTTTTTCTCAAGTTGTCAAAATGCCAGT
BY809 AACAAGTGTTCAAGCAGCTCAGGTTGCACCAGTTTTTCTCAAGTTGTCAAAATGCCAGT
****

Ref-Unigene_503 TTGCTCAGCTGACAATTGAGTATTTAGGACATGTGATCTCAGCAGCTGGAGTTGCAACCG
303WX TTGCTCAGCTGACAATTGAGTATTTAGGACATGTGATCTCAGCAGCTGGAGTTGCAACCG
GEMS1 TTGCTCAGCTGACAATTGAGTATTTAGGACATGTGATCTCAGCAGCTGGAGTTGCAACCG
GEMS51 TTGCTCAGCTGACAATTGAGTATTTAGGACATGTGATCTCAGCAGCTGGAGTTGCAACCG
CML422 TTGCTCAGCTGACAATTGAGTATTTAGGACATGTGATCTCAGCAGCTGGAGTTGCAACCG
BY809 TTGCTCAGCTGACAATTGAGTATTTAGGACATGTGATCTCAGCAGCTGGAGTTGCAACCG
****

Ref-Unigene_503 ATCCAGCAAAGACAGAGGCTATGC
303WX ATCCAGCAAAGACAGAGGCTATGC
GEMS1 ATCCAGCAAAGACAGAGGCTATGC
GEMS51 ATCCAGCAAAGACAGAGGCTATGC
CML422 ATCCAGCAAAGACAGAGGCTATGC
BY809 ATCCAGCAAAGACAGAGGCTATGC
****

Unigene_741 [383-692;930]

Ref-Unigene_741 ATGATATACTTAGGCATGTGGTACATCTTGGGAGGCACACCTGCTCATGTAGAGAGTGGC
GEMS48 ATGATATACTTAGGCATGTGGTACATCTTGGGAGGCACACCTGCTCATGTAGAGAGTGGC
GEMS51 ATGATATACTTAGGCATGTGGTACATCTTGGGAGGCACACCTGCTCATGTAGAGAGTGGC
GEMS1 ATGATATACTTAGGCATGTGGTACATCTTGGGAGGCACACCTGCTCATGTAGAGAGTGGC
R15X1141 ATGATATACTTAGGCATGTGGTACATCTTGGGAGGCACACCTGCTCATGTAGAGAGTGGC
303WX ATGATATACTTAGGCATGTGGTACATCTTGGGAGGCACACCTGCTCATGTAGAGAGTGGC
****

Ref-Unigene_741 AGGTTTCTGGAAAACCATGTCCACATGCATTGACACTCATAATTACAACTAGAAATCCAA
GEMS48 AGGTTTCTGGAAAACCATGTCCACATGCATTGGCACTCATAATTACAACTAGAAATCCAA
GEMS51 AGGTTTCTGGAAAACCATGTCCACATGCATTGGCACTCATAATTACAACTAGAAATCCAA
GEMS1 AGGTTTCTGGAAAACCATGTCCACATGCATTGGCACTCATAATTACAACTAGAAATCCAA
R15X1141 AGGTTTCTGGAAAACCATGTCCACATGCATTGGCACTCATAATTACAACTAGAAATCCAA
303WX AGGTTTCTGGAAAACCATGTCCACATGCATTGGCACTCATAATTACAACTAGAAATCCAA
******************************** ***************************

Ref-Unigene_741 AATTGGGTGATTATTTGCATCCTTACTATTCAGTGTACCATTTTAGGCTAGCATATGCAG
GEMS48 AATTGGGTGATTATTTGCATCCTTACTATTCAGTGTACCATTTTAGGCTAGCATATGCAG
GEMS51 AATTGGGTGATTATTTGCATCCTTACTATTCAGTGTACCATTTTAGGCTAGCATATGCAG
GEMS1 AATTGGGTGATTATTTGCATCCTTACTATTCAGTGTACCATTTTAGGCTAGCATATGCAG
R15X1141 AATTGGGTGATTATTTGCATCCTTACTATTCAGTGTACCATTTTAGGCTAGCATATGCAG
303WX AATTGGGTGATTATTTGCATCCTTACTATTCAGTGTACCATTTTAGGCTAGCATATGCAG
****

Ref-Unigene_741 GTGTTATTCAACCATTAACAGATAAGTCTCAATGGCCGAAAGTCAACCTTGGCTTCAAGT
GEMS48 GTGTTATTCAACCATTAACAGATAAGTCTCAATGGCCGAAAGTCAACCTTGGCTTCAAGT
GEMS51 GTGTTATTCAACCATTAACAGATAAGTCTCAATGGCCGAAAGTCAACCTTGGCTTCAAGT
GEMS1 GTGTTATTCAACCATTAACAGATAAGTCTCAATGGCCGAAAGTCAACCTTGGCTTCAAGT
R15X1141 GTGTTATTCAACCATTAACAGATAAGTCTCAATGGCCGAAAGTCAACCTTGGCTTCAAGT
303WX GTGTTATTCAACCATTAACAGATAAGTCTCAATGGCCGAAAGTCAACCTTGGCTTCAAGT
****

Ref-Unigene_741 TATTGCCACCTCTGGTAAAAAGATCAGTAGGTAGACAATGGAAGAACAGGATAAAAGGAT
GEMS48 TATTGCCACCTCTGGTAAAAAGATCAGTAGGTAGACAATGGAAGAACAGGATAAAAGGAT
GEMS51 TATTGCCACCTCTGGTAAAAAGATCAGTAGGTAGACAATGGAAGAACAGGATAAAAGGAT
GEMS1 TATTGCCACCTCTGGTAAAAAGATCAGTAGGTAGACAATGGAAGAACAGGATAAAAGGAT
R15X1141 TATTGCCACCTCTGGTAAAAAGATCAGTAGGTAGACAATGGAAGAACAGGATAAAAGGAT
303WX TATTGCCACCTCTGGTAAAAAGATCAGTAGGTAGACAATGGAAGAACAGGATAAAAGGAT
****

Ref-Unigene_741 GCCTGGAGAA
GEMS48 GCCTGGAGAA
GEMS51 GCCTGGAGAA
GEMS1 GCCTGGAGAA
R15X1141 GCCTGGAGAA
303WX GCCTGGAGAA
****

Unigene_1536 [781-1084;1234]

Ref-Unigene_1536 AGGATGCATTATATACCTTGCTAAGCAATTATTACGGCGTAACGGAGATCGACCACCTTG
GEMS1 AGGATGCATTATATACCTTGCTAAGCAATTATTACGGCGTAACGGAGATCGACCACCTTG
R15X1141 AGGATGCATTATATACCTTGCTAAGCAATTATTACGGCGTAACGGAGATCGACCACCTTG
303WX AGGATGCATTATATACCTTGCTAAGCAATTATTACGGCGTAACGGAGATCGACCACCTTG
GEMS48 AGGATGCATTATATACCTTGCTAAGCAATTATTACGGCGTAACGGAGATCGACCACCTTG
GEMS51 AGGATGCATTATATACCTTGCTAAGCAATTATTACGGCGTAACGGAGATCGACCACCTTG
****

Ref-Unigene_1536 ACTCATTGGAACGTAGCCATATATCTGGCAGTTCTAGCATATATCTGATCAAAGCAAAGC
GEMS1 ACTCATTGGAACGTAGCCATATATCTGGCAGTTCTAGCATATATCTGATCAAAGCAAAGC
R15X1141 ACTCATTGGAACGTAGCCATATATCTGGCAGTTCTAGCATATATCTGATCAAAGCAAAGC
303WX ACTCATTGGAACGTAGCCATATATCTGGCAGTTCTAGCATATATCTGATCAAAGCAAAGC
GEMS48 ACTCATTGGAACGTAGCCATATATCTGGCAGTTCTAGCATATATCTGATCAAAGCAAAGC
GEMS51 ACTCATTGGAACGTAGCCATATATCTGGCAGTTCTAGCATATATCTGATCAAAGCAAAGC
****

Ref-Unigene_1536 CATATATCTGGCAGTTCTGGGGTGATCCGTCCGTGGAAAAGGCTTGGCCACTGACCTGCG
GEMS1 CATATATCTGGCAGTTCTGGGGTGATCCGTCCGTGGAAAAGGCTTGGCCACTGACCTGCG
R15X1141 CATATATCTGGCAGTTCTGGGGTGATCCGTCCGTGGAAAAGGCTTGGCCACTGACCTGCG
303WX CATATATCTGGCAGTTCTGGGGTGATCCGTCCGTGGAAAAGGCTTGGCCACTGACCTGCG
GEMS48 CATATATCTGGCAGTTCTGGGGTGATCCGTCCGCGGAGAAGGCTTGGCTACTGACCTGCG
GEMS51 CATATATCTGGCAGTTCTGGGGTGATCCGTCCGCGGAGAAGGCTTGGCCACTGACCTGCG
********************************* *** ********** ***********

Ref-Unigene_1536 CG----AGGACAAAGCAGTAGAAACATATCAGCCAATCAAAGCAAAGCCATATGAATTCA
GEMS1 CG----AGGACAAAGCAGTAGAAACATATCAGCCAATCAAAGCAAAGCCATATGAATTCA
R15X1141 CG----AGGACAAAGCAGTAGAAACATATCAGCCAATCAAAGCAAAGCCATATGAATTCA
303WX CG----AGGACAAAGCAGTAGAAACATATCAGCCAATCAAAGCAAAGCCATATGAATTCA
GEMS48 CGCGCGAGGACAAAGCAGTAGAAACATATCAGCCAATCAAAGCAAAGCCATATGAATTCA
GEMS51 CGCGCGAGGACAAAGCAGTAGAAACATATCAGCCAATCAAAGCAAAGCCATATGAATTCA
**   **

Ref-Unigene_1536 TGTTAAACATGTCCATACATATAGCAGTGAAAAGTATCAAATCACTGATTTCCTTTCTTG
GEMS1 TGTTAAACATGTCCATACATATAGCAGTGAAAAGTATCAAATCACTGATTTCCTTTCTTG

R15X1141 TGTTAAACATGTCCATACATATAGCAGTGAAAAGTATCAAATCACTGATTTCCTTTCTTG
303WX TGTTAAACATGTCCATACATATAGCAGTGAAAAGTATCAAATCACTGATTTCCTTTCTTG
GEMS48 TGTTAAACATGTCCATACATATAGCAGTGAAAAGTATCAAATCACTGATTTCCTTTCTTG
GEMS51 TGTTAAACATGTCCATACATATAGCAGTGAAAAGTATCAAATCACTGATTTCCTTTCTTG
****

Ref-Unigene_1536 GCCTTTGTAACTGTATATACTGCTTTCGTGGCCTTTGT
GEMS1 GCCTTTGTAACTGTATATACTGCTTTCGTGGCCTTTGT
R15X1141 GCCTTTGTAACTGTATATACTGCTTTCGGGGCCTTTGT
303WX GCCTTTGTAACTGTATATACTGCTTTCGGGGCCTTTGT
GEMS48 GCCTTTGTAACTGTACATACTGCTTTCGGGGCCTTTGT
GEMS51 GCCTTTGTAACTGTACATACTGCTTTCGGGGCCTTTGT
*************** ************* *********

Unigene_1931 [693-920;1087]

Ref-Unigene_1931 ACTTGATTGTGACTGCCTCGTACGTTCCTCGTGCAAAATCACTAAGG------------
CML422 ACTTGATTGTGACTGCCTCGTACGTTCCTCGTGCAAAATCACTAAGGTAATTCTTTTTT
B77 ACTTGATTGTGACTGCCTCGTACGTTCCTCGTGCAAAATCACTAAGGTAATTCTTTTTT
303WX ACTTGATTGTGACTGCCTCGTACGTTCCTCGTGCAAAATCACTAAGGTAATTCTTTTTT
GEMS48 ACTTGATTGTGACTGCCTCGTACGTTCCTCGTGCAAAATCACTAAGGTAATTCTTTTTT
GEMS1 ACTTGATTGTGACTGCCTCGTACGTTCCTCGTGCAAAATCACTAAGGTAATTCTTTTTT
****

Ref-Unigene_1931 --
CML422 TATTGCCTGTTTTTTTATGGTGAGTTAGTATATAGTTGTATCCGAGTGAGTTGGCTAGCA
B77 TATTGCCTGTTTTTTTATGGTGAGTTAGTATATAGTTGTATCCGAGTGAGTTGGCTAGCA
303WX TATTGCCTGTTTTTTTATGGTGAGTTAGTATATAGTTGTATCCGAGTGAGTTGGCTAGCA
GEMS48 TATTGCCTGTTTTTTTATGGTGAGTTAGTATATAGTTGTATCCGAGTGAGTTGGCTAGCA
GEMS1 -ATTGCCTGTTTTTTTATGGTGAGTTAGTATATAGTTGTATCCGAGTGAGTTGGCTAGCA

Ref-Unigene_1931 ---CTGGGATCGGA
CML422 ATACTTATTTGGAATAGAAATCACATTTGCTCCATGTTGCCTTGCTAGGCTGGGATCGGA
B77 ATACTTATTTGGAATAGAAATCACATTTGCTCCATGTTGCCTTGCTAGGCTGGGATCGGA
303WX ATACTTATTTGGAATAGAAATCACATTTGCTCCATGTTGCCTTGCTAGGCTGGGATCGGA
GEMS48 ATACTTATTTGGAATAGAAATCACATTTGCTCCATGTTGCCTTGCTAGGCTGGGATCGGA
GEMS1 ATACTTATTTGGAATAGAAATCACATTTGCTCCATGTTGCCTTGCTAGGCTGGGATCGGA
****

Ref-Unigene_1931 GGCCCCCTTGTTACTCTAGATGGGAATGTTCTCGGCATGAACTTCTATGATAAGAAAATA
CML422 GGCCCCCTTGTTACTCTAGATGGGAATGTTCTCGGCATGAACTTCTATGATAAGAAAATA
B77 GGCCCCCTTGTTACTCTAGATGGGAATGTTCTCGGCATGAACTTCTATGATAAGAAAATA
303WX GGCCCCCTTGTTACTCTAGATGGGAATGTTCTCGGCATGAACTTCTATGATAAGAAAATA
GEMS48 GGCCCCCTTGTTACTCTAGATGGGAATGTTCTCGGCATGAACTTCTATGATAAGAAAATA
GEMS1 GGCCCCCTTGTTACTCTAGATGGGAATGTTCTCGGCATGAACTTCTATGATAAGAAAATA
****

Ref-Unigene_1931 GGAACCCCTTTCCTGTCAATGCCGGACATTTTCTTGATTTTAAAATCGTCTAAG------
CML422 GGAACCCCTTTCCTGTCAATGCCGGACATTTTCTTGATTTTAAAATCGTCTAAGAGGTAT
B77 GGAACCCCTTTCCTGTCAATGCCGGACATTTTCTTGATTTTAAAATCGTCTAAGGGGTAT
303WX GGAACCCCTTTCCTGTCAATGCCGGACATTTTCTTGATTTTAAAATCGTCTAAGAGGTAT

GEMS48 GGAACCCCTTTCCTGTCAATGCCGGACATTTTCTTGATTTAAAATCGTCTAAGAGGTAT
GEMS1 GGAACCCCTTTCCTGTCAATGCCGGACATTTTCTTGATTTAAAATCGTCTAAGAGGTAT
****

Ref-Unigene_1931 --
CML422 TTTAAGTGCATATCACATGTTATTTGTCTGAACATTTTAAGAAGCTTGCCGAACCAGGTT
B77 TTTAAGTGCATATCACATGTTATTTGTCTGAACATTTTAAGAAGCTTGCCGAACCAGGTT
303WX TTTAAGTGCATATCACATGTTATTTGTCTGAACATTTTAAGAAGCTTGCCGAACCAGGTT
GEMS48 TTTAAGTGCATATCACATGTTATTTGTCTGAACATTTTAAGAAGCTTGCCGAACCAGGTT
GEMS1 TTTAAGTGCATATCACATGTTATTTGTCTGAACATTTTAAGAAGCTTGCCGAACCAGGTT

Ref-Unigene_1931 ----------------------------------AGTCACCCCTCTGCTGGGCCTTTCT
CML422 GAGTGAAGCATTAACTATTTAATGGCATGCTTGACAGTCACCCCTCTGCTGGGCCTTTCT
B77 GAGTGAAGCATTAACTATTTAATGGCATGCTTGACAGTCACCCCTCTGCTGGGCCTTTCT
303WX GAGTGAAGCATTAACTATTTAATGGCATGCTTGACAGTCACCCCTCTGCTGGGCCTTTCT
GEMS48 GAGTGAAGCATTAACTATTTAATGGCATGCTTGACAGTCACCCCTCTGCTGGGCCTTTCT
GEMS1 GAGTGAAGCATTAACTATTTAATGGCATGCTTGACAGTCACCCCTCTGCTGGGCCTTTCT
****

Ref-Unigene_1931 GGAAAATGGATGGGGATGACGCAGCTAGGTT
CML422 GGAAAATGGATGGGGATGACGCAGCTAGGTT
B77 GGAAAATGGATGGGGATGACGCAGCTAGGTT
303WX GGAAAATGGATGGGGATGACGCAGCTAGGTT
GEMS48 GGAAAATGGATGGGGATGACGCAGCTAGGTT
GEMS1 GGAAAATGGATGGGGATGACGCAGCTAGGTT
****

Unigene_678 [366-643; 278]

Ref-Unigene_678 TCCGAAGCGACTCTATGATCTAGACTGACTTCCTTAAGACATTTGAGGCGGTTGATTTG
B73 -----AGCGACTCTATGATCTAGACTGACTTCCTTAAGACATTTGAGGCGGTTGATTTG
****

Ref-Unigene_678 ATGTCACCGAGGCCAACTAGATCTTTGCAGATTAGCTTAAGTGAGATGAGGTACGGCAAA
B73 ATGTCACCGAGGCCAACTAGATCTTTGCAGATTAGCTTAAGTGAGATGAGGTACGGCAAA
****

Ref-Unigene_678 GCTCCTCCTTCAATTATTGGTAAGGTGGCACGTTCTAGGACAAAACATAGGTGTAGCAGC
B73 GCTCCTCCTTCAATTATTGGTAAGGTGGCACGTTCTAGGACAAAACATAGGTGTAGCAGC
****

Ref-Unigene_678 CCCAGGAATGCCTGACCTTCAATGATAAAATCTTCAAGGACATCTGCAATCAGCTTGAGA
B73 CCCAGGAATGCCTGACCTTCAATGATAAAATCTTCAAGGACATCTGCAATCAGCTTGAGA
****

Ref-Unigene_678 TGCTGCAAGCCTTTCAAGTTAGCGAGTGTTGCAAGGAGGCCCG
B73 TGCTGCAAGCCTTTCAAGTTAGCGAGTGTTGCAAGGAGGCCCG
****

Unigene_678 [928-1154; 227]

Ref-Unigene_678 TTCATGTAATTCATCATCTGTGGCAATCCTTGAGACCCATCTGTGATAAATCCTGAAAGT
B73 TTCATGTAATTCATCATCTGTGGCAATCCTTGAGACCCATCTGTGATAAATCCTGAAAGT

****

```
Ref-Unigene_678    GTCTCTAAGTTACTCTGTCCAGTTAAGAAAAACTTCTGCATGTCACTTGTTATCTTGATT
B73                GTCTCTAAGTTACTCTGTCCAGTTAAGAAAAACTTCTGCATGTCACTTGTTATCTTGATT
                   ************************************************************
Ref-Unigene_678    TTATCAGAAAATTGGAACTTCCCAAACAGATGGAGTAAACAGGGGAGCAGGAGGACCTCT
B73                TTATCAGAAAATTGGAACTTCCCAAACAGATGGAGTAAACAGGGGAGCAGGAGGACCTCT
                   ************************************************************
Ref-Unigene_678    ATAGGTAGTGTTTTCACCTTTGTTCTCCTCAAGTCAAGTGTCTCCAA
B73                ATAGGTAGTGTTTTCACCTTTGTTCTCCTCAAGTCAAGTGTCTCCAA
                   ***********************************************
```

Unigene_678 [1460-1766; 307]

```
Ref-Unigene_678    GCAGGGTAATGAAGTCATGGGAGGTGGACATTAACAAAATGAACTCGTGCATCATGCCAT
B73                GCAGGATAATGAAGTCATGGGAGGTGGACATTAACAAAATGAACTCGTGCATCATGCCAT
                   ************************************************************
Ref-Unigene_678    ATGTTTTGCATGTCTTTACCTGATCATCGTTACATATGCCGATGGGCTCAATAATATTCC
B73                ATGTTTTGCATGTCTTTACCTGATCATCGTTACATATGCCGATGGGCTCAATAATATTCC
                   ************************************************************

Ref-Unigene_678    GGTCTATGAGGGTGTTGAAGTTTTCACTAGATGAAGGCTGTGTTTGTACAAATCCCTCTG
B73                GGTCTATGAGGGTGTTGAAGTTTTCACTAGATGAAGGCTGTGTTTGTACAAATCCCTCTG
                   ************************************************************
Ref-Unigene_678    CTAACCATCGCCTCATCAGGCTCTTCCTCTTTATGGGATGATCACATGGAAACATACCAA
B73                CTAACCATCGCCTCATCAGGCTCTTCCTCTTTATGGGATGATCACATGGAAACATACCAA
                   ************************************************************
Ref-Unigene_678    AATACAAAAGGCAGGCTTTGGGAACATGGCTAGGAAGACTAGATAAGCTGTGGATAAGCA
B73                AATACAAAAGGCAGGCTTTGGGAACATGGCTAGGAAGACTAGATAAGCTGTGGATAAGCA
                   ************************************************************
Ref-Unigene_678    CCCTTCG
B73                CCCTTCG
                   *******
```

Unigene_705 [520-648; 129]

```
Ref-Unigene_705    TTGACTACAATGGTGAGGTGTTGTCGTGTACAGTGCACCATACTATATTTGATCTC
                   ATTA
CIMBL38            TTGACTACAATGGTGAGGTGTTGTCGTGTACAGTGCACCATACTATATTTGATCTCATTA
                   ************************************************************
Ref-Unigene_705    ATTACAATTCCAAGGAAGAGGAATTTATTGCTGGAATAGATTACTCTCAACCAATAACAG
CIMBL38            ATTACAATTCCAAGGAAGAGGAATTTATTGCTGGAATAGATTACTCTCAACCAATAACAG
                   ************************************************************
Ref-Unigene_705    GACTTGCTA
CIMBL38            GACTTGCTA
                   *********
```

Unigene_705 [967-1262; 299]

```
Ref-Unigene_705    GACTATCTGCAGTTCCATTGGATATTGTCCATCTTCCAAGTTTGTTGCATCTTAGTCTCC
CIMBL38            GACTATCTGCAGTTCCATTGGATATTGTCCATCTTCCAAGTTTGTTGCATCTTAGTCTCC
                   ************************************************************
Ref-Unigene_705    GAGCTGCAACTAAGTTACCAGATGGGATTGGCCACATCAAATCCCTAAGTACGCTATTGT
CIMBL38            GAGCTGCAACTAAGTTACCAGATGGGATTGGCCACATCAAATCCCTAAGTACGCTATTGT
```

****

Ref-Unigene_705 ATTTTGACCTCAGATGTAACTCTGAAGACAATATACGGAGCTTAGGACAGCTGACGAACC
CIMBL38 ATTTTGACCTCAGATGTAACTCTGAAGACAATATACGGAGCTTAGGACAGCTGACGAACC
****

Ref-Unigene_705 TTCGACATCTTCATCTAACCTGTTCTACAGTTCTCTCCAGTGACCACCTGAAGAGAAAGC
CIMBL38 TTCGACATCTTCATCTAACCTGTTCTACAGTTCTCTCCAGTGACCACCTGAAGAGAAAGC
****

Ref-Unigene_705 TGATACCTCTAGCCTTTTCTCTTGGGAAACTTGGCAATCTCAAATCTCTCACCCTG
CIMBL38 TGATACCTCTAGCCTTTTCTCTTGGGAAACTTGGCAATCTCAAATCTCTCACCCTG
****

Unigene_705 [1299-1744; 446]

Ref-Unigene_705 ATCTCGAGCGGTATCTCCTCTCCTTCCATCTTTCTTCAGAAACTTGAGTTGTTGCCACCA
CIMBL38 ATCTCGAGCGGTATCTCCTCTCCTTCCATCTTTCTTCAGAAACTTGAGTTGTTGCCACCA
****

Ref-Unigene_705 ATTTGCTTCTTTTCCAGACTGCCCGCTTGCTTTGGTGAACTGCACAAACTCCGCATTTTG
CIMBL38 ATTTGCTTCTTTTCCAGACTGCCCGCTTGCTTTGGTGAACTGCACAAACTCCGCATTTTG
****

Ref-Unigene_705 AAAATTGTGGTGAAAGAACTGCAGGGAAATGATATTAACAACATTGCTGGATTACCTTCC
CIMBL38 AAAATTGTGGTGAAAGAACTGCAGGGAAATGATATTAACAACATTGCTGGATTACCTTCC
****

Ref-Unigene_705 CTAGTGATTTTCTCACTGTATGTGCGGACAGCTCTGACTGGAACTGTGATCTTCAGCACC
CIMBL38 CTAGTGATTTTCTCACTGTATGTGCGGACAGCTCTGACTGGAACTGTGATCTTCAGCACC
****

Ref-Unigene_705 ATGTCATTCCCAGCTCTCAAGTACTTCAGATTCACATGTGGTGTGACGTGCTTGGCTTTT
CIMBL38 ATGTCATTCCCAGCTCTCAAGTACTTCAGATTCACATGTGGTGTGACGTGCTTGGCTTTT
****

Ref-Unigene_705 CAGGAAGGAGCCATGCACAGACTTCAAAGGCTCAAGCTTTGTTTCAATGCCCATGGAGGC
CIMBL38
CAGGAAGGAGCCATGCACAGACTTCAAAGGCTCAAGCTTTGTTTCAATGCCCATGGAGGC
****

Ref-Unigene_705 AAGAACCATAGCCGAGTGATTGACGGCATTGAGTACCTGTTAAACCTTCAGGAGGTTTCT
CIMBL38 AAGAACCATAGCCGAGTGATTGACGGCATTGAGTACCTGTTAAACCTTCAGGAGGTTTCT
****

Ref-Unigene_705 GGACAAATTGGGGTCCTCCCAGGTGG
CIMBL38 GGACAAATTGGGGTCCTCCCAGGTGG
****

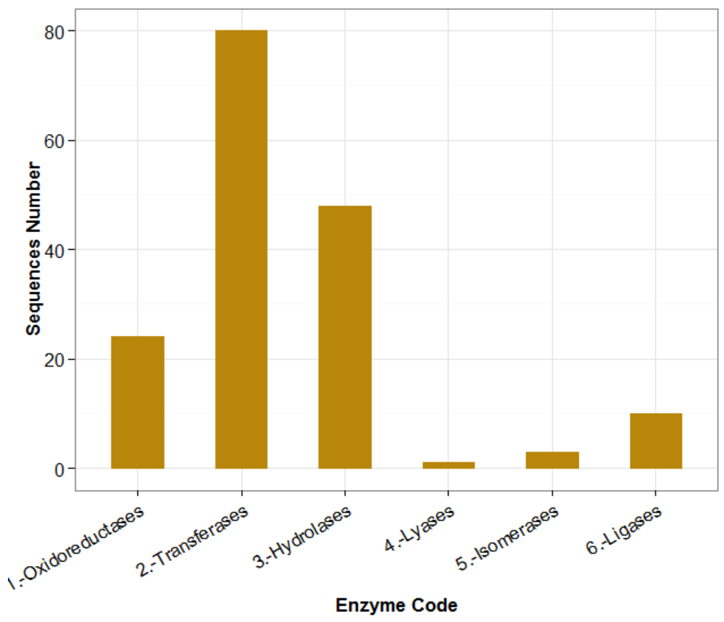

Supplementary Fig. S10. Distribution of six enzyme codes of annotated novel genes.

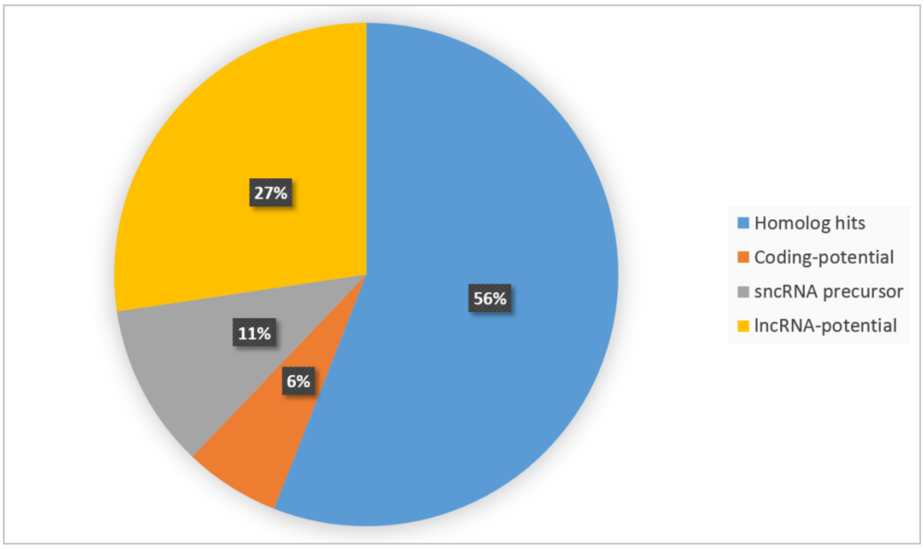

Supplementary Fig. S11. Pie chart of statistics on final annotation of novel genes

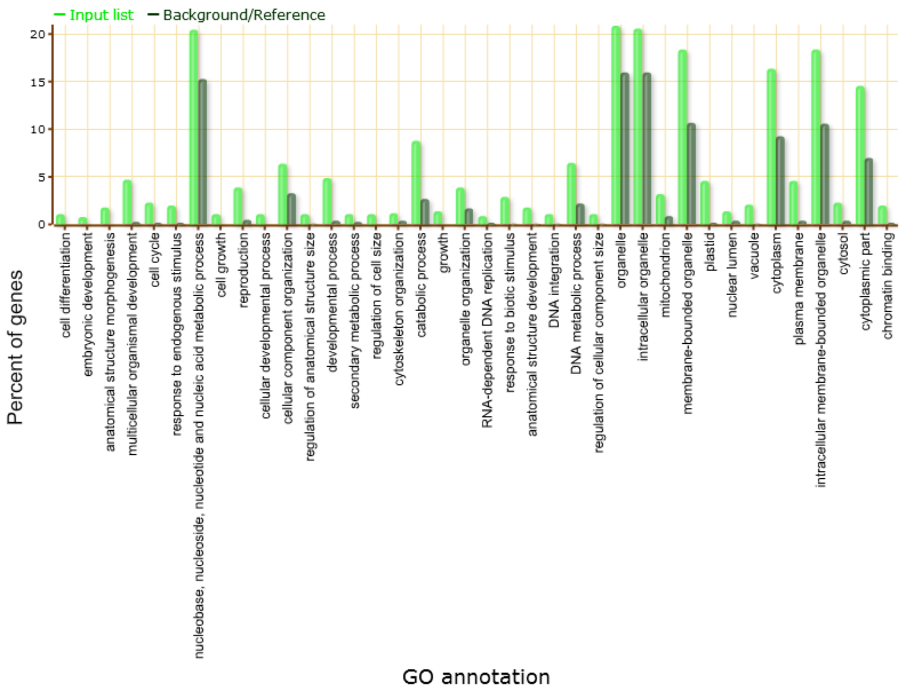

Supplementary Fig. S12. GO enrichment analysis of novel genes in comparison with the maize B73 reference.

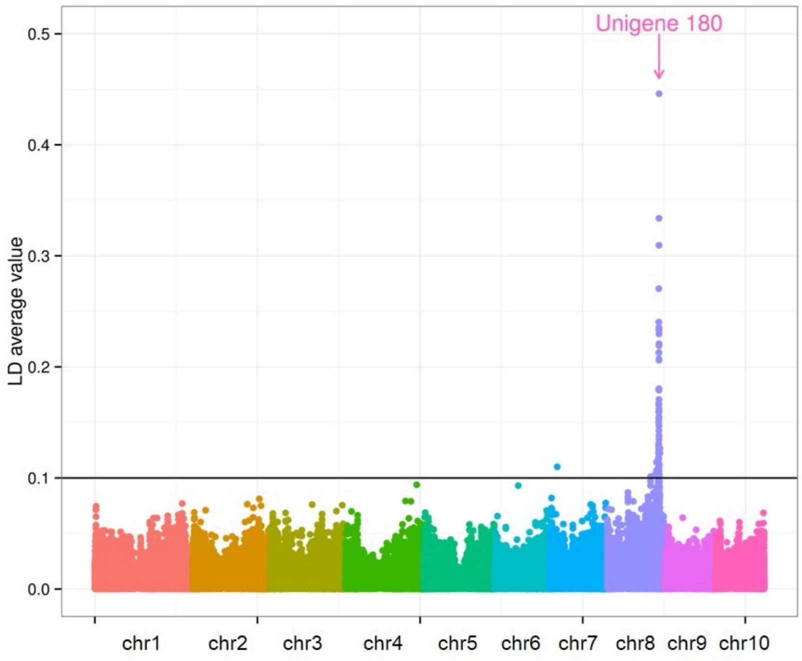

Supplementary Fig. S13. An example the use of linkage disequilibrium (LD) mapping of a novel gene, Unigene 180, to the reference genome

Different color represent different chromosomes. Y-axis is the average measure of LD, r^2 (pairwise, between the novel gene and markers along the 10 maize chromosomes.) The black horizontal line is the LD cutoff value.

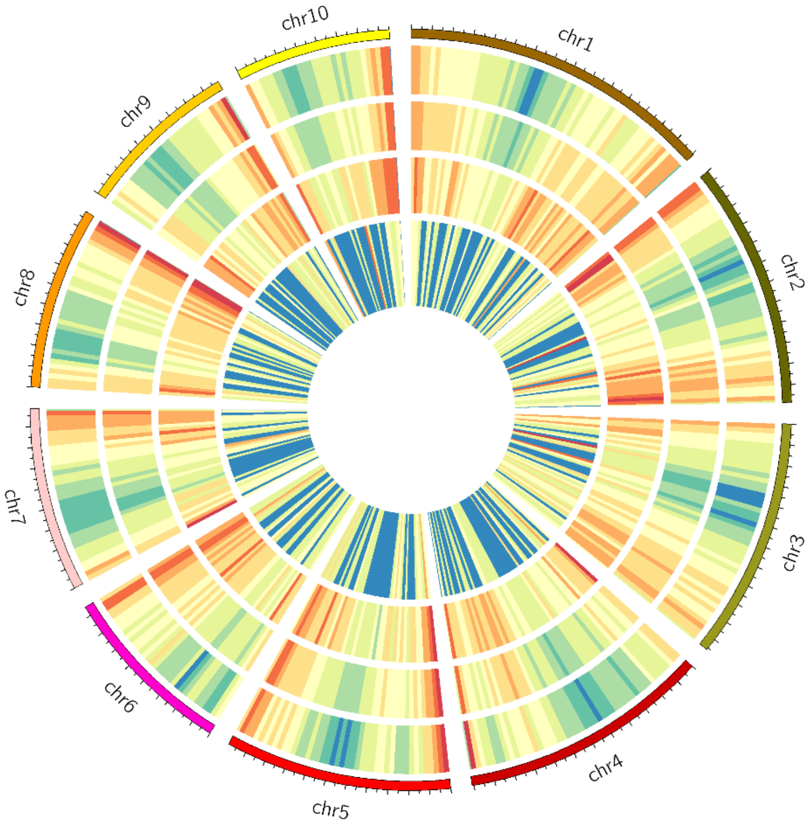

Supplementary Fig. S14. The chromosome distribution of different kinds of variations.
The heatmap represents the density of the different types of variations, from low (blue) to high (red). Variations from outside to inside: SNPs, common expression genes, ePAV candidates, and mapped novel sequences.

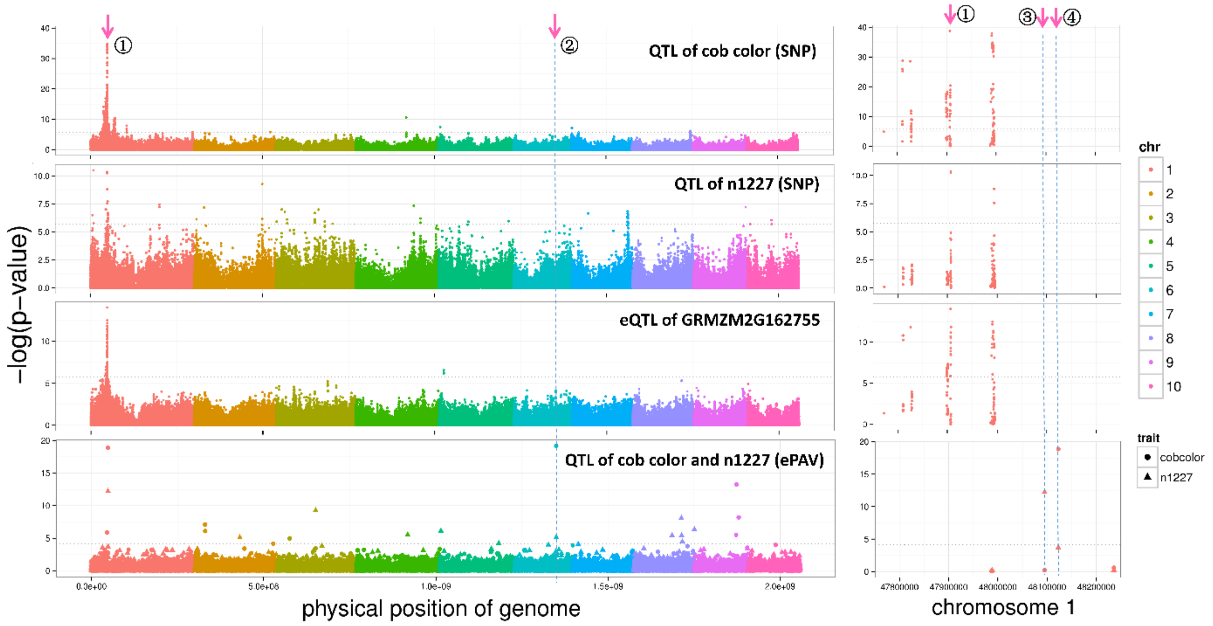

Supplementary Fig. S15. Exploring maize genetics using ePAV.

Genome-wide (a) and detailed view (b) of the significant region within chromosome 1. Manhattan plot of GWAS log(p) values. Panel 1 and panel 2: Use of SNP polymorphisms for the mapping the cob color and flavonoid metabolite concentration (n1227). Panel 3: eQTL mapping of GRMZM2G162755, anthocyanidin 3-O-glucosyltransferase, which is a candidate gene for cob color and several flavonoids. Panel 4: Use ePAV variation for mapping the cob color and flavonoid metabolite (n1227). ① is the most significant locus of panels 1, 2 and 3; ②, ③ and ④were three candidates for cob color and several flavonoid metabolites; GRMZM2G162755, GRMZM2G162755, GRMZM2G084799. Ten different colors represent 10 chromosomes. Circles and triangles represent different shapes represent the two traits in panel 4.

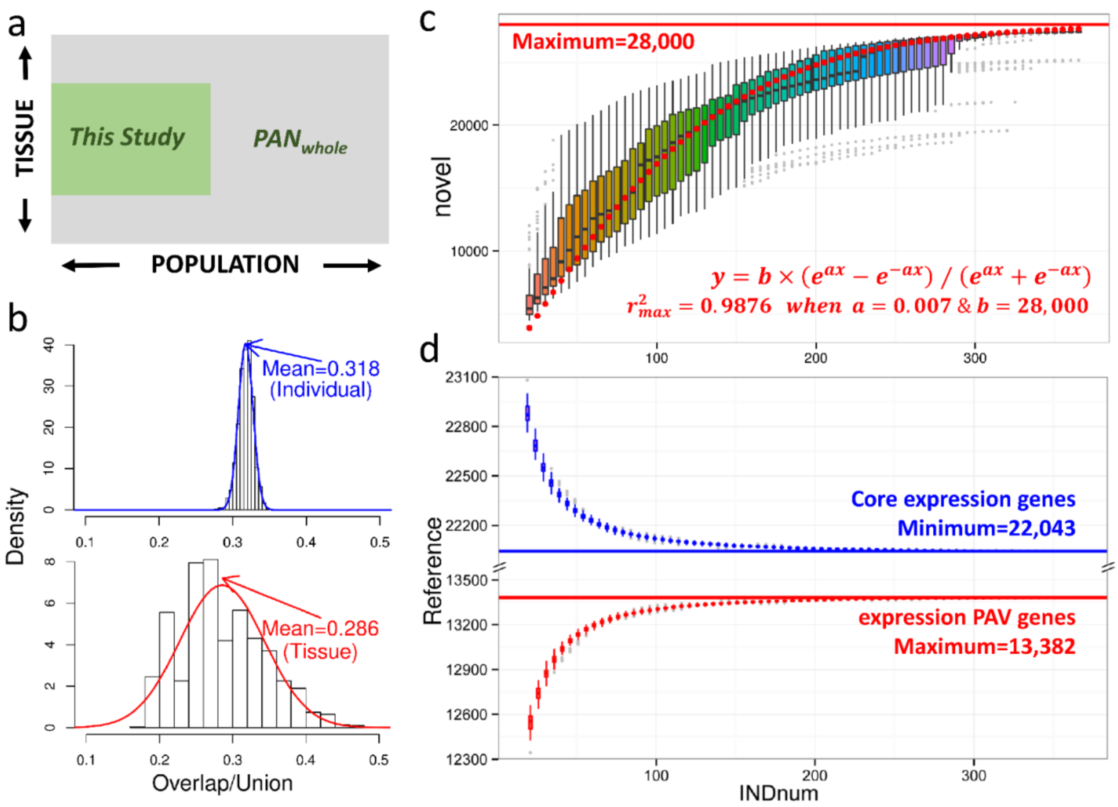

Supplementary Fig. S16. Estimation of maize pan-transcriptome size.

By using the identified novel sequences in this study based on the 368 individuals and kernel tissue, we try to estimate the possible pan-transcriptome size for the whole maize community, and to distinguish the reference genes as core genes or dispensable ones. **(a)** Tissues types and population size were the two key factors to influence the size of pan-transcriptome. Our study here covered a portion of maize whole pan-transcriptome. **(b)** The ratio of shared genes to total genes (while the less means more ratio of novel genes) was used to compare efficiency in discovering novel transcripts of individual (above) and tissue (below). **(c)** The simulation results between lines number and novel sequences number was shown. The increase of the novel sequences in different clustering runs decayed with the increase of lines number and expected to be a maximum constant value 28,000. **(d)** Core expression genes and ePAV genes number both reached almost invariable when considering more than 200 lines.

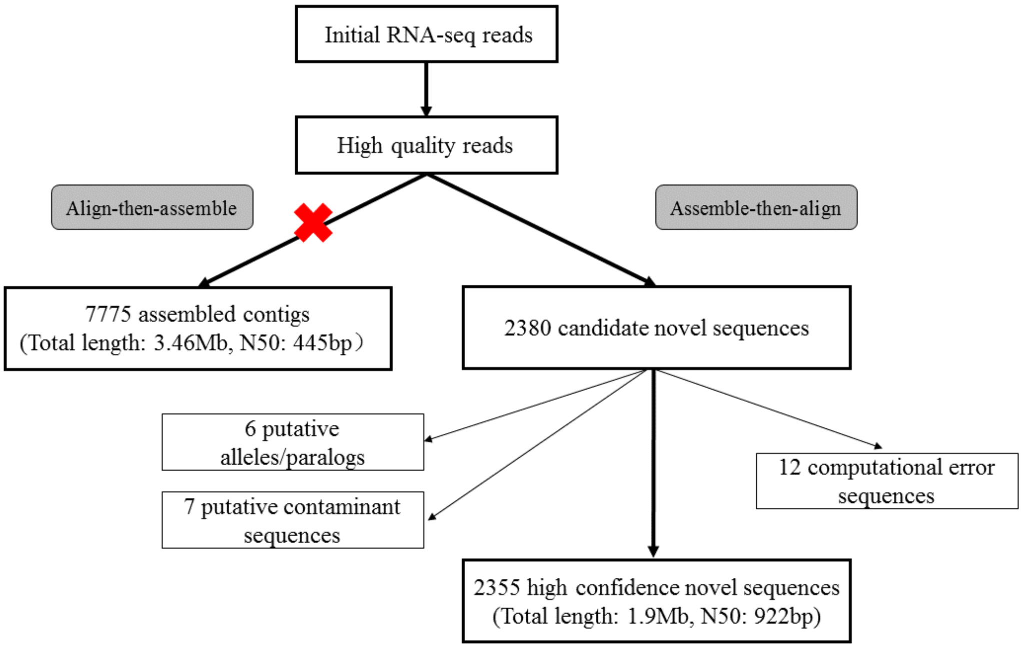

Supplementary Fig. S17. Procedure of *de novo* assembly.

Supplementary Table S1. Detail information of enriched GO terms in ePAV and non-ePAV gene sets.

GO term	Description	NO.in BG/Ref	NO. in non-ePAV	p-value	FDR	NO. in ePAV	pvalue	FDR
GO:0009987*	cellular process	18476	7282	1.20E-22	8.40E-19	2393	1.00E+00	1.00E+00
GO:0044237*	cellular metabolic process	14490	5679	9.50E-14	3.20E-10	1867	1.00E+00	1.00E+00
GO:0044238*	primary metabolic process	15885	6119	8.60E-11	1.90E-07	2168	8.30E-01	1.00E+00
GO:0051179*	localization	3887	1651	5.60E-10	9.60E-07	505	9.30E-01	1.00E+00
GO:0044260*	cellular macromolecule metabolic process	11296	4425	7.10E-10	9.70E-07	1488	9.90E-01	1.00E+00
GO:0051234*	establishment of localization	3841	1628	1.20E-09	1.10E-06	498	9.40E-01	1.00E+00
GO:0006810*	transport	3841	1628	1.20E-09	1.10E-06	498	9.40E-01	1.00E+00
GO:0044267*	cellular protein metabolic process	5860	2391	2.80E-09	2.30E-06	688	1.00E+00	1.00E+00
GO:0042592*	homeostatic process	2047	907	2.90E-08	2.10E-05	341	9.10E-04	5.40E-02
GO:0065008*	regulation of biological quality	2139	943	3.10E-08	2.10E-05	356	7.50E-04	4.60E-02
GO:0009058	biosynthetic process	7998	3160	6.80E-08	4.20E-05	1083	8.10E-01	1.00E+00
GO:0043412	macromolecule modification	3252	1364	1.80E-07	8.20E-05	458	3.90E-01	1.00E+00
GO:0006464	protein modification process	3113	1311	1.70E-07	8.20E-05	452	1.80E-01	1.00E+00
GO:0050896	response to stimulus	3421	1429	1.80E-07	8.20E-05	627	3.90E-11	1.00E-08
GO:0008152	metabolic process	20231	7591	1.70E-07	8.20E-05	2938	4.90E-04	3.50E-02
GO:0009628	response to abiotic stimulus	1856	819	2.30E-07	9.20E-05	314	6.20E-04	4.30E-02
GO:0043170	macromolecule metabolic process	12696	4872	2.20E-07	9.20E-05	1723	8.60E-01	1.00E+00
GO:0032501	multicellular organismal process	1983	868	2.70E-07	0.0001	339	0.0002	0.018
GO:0065007	biological regulation	6272	2501	3.30E-07	0.00012	1054	3.7E-10	7.3E-08
GO:0044249	cellular biosynthetic process	7619	3000	4.20E-07	0.00014	1030	0.82	1
GO:0009266	response to temperature stimulus	1765	776	7.30E-07	0.00023	298	0.00094	0.055
GO:0001659	temperature homeostasis	1758	770	1.20E-06	0.0003	298	0.00076	0.046
GO:0051641	cellular localization	817	392	1.20E-06	0.0003	56	1	1
GO:0050826	response to freezing	1758	770	1.20E-06	0.0003	298	0.00076	0.046
GO:0009409	response to cold	1758	770	1.20E-06	0.0003	298	0.00076	0.046
GO:0042309	homoiothermy	1758	770	1.20E-06	0.0003	298	0.00076	0.046
GO:0048871	multicellular organismal homeostasis	1758	770	1.20E-06	0.0003	298	0.00076	0.046
GO:0016070	RNA metabolic process	3121	1299	1.20E-06	0.0003	442	0.35	1
GO:0046907	intracellular transport	661	326	1.50E-06	0.00036	35	1	1
GO:0051649	establishment of localization in cell	791	377	3.00E-06	0.00069	56	1	1
GO:0043687	post-translational protein modification	2929	1217	3.30E-06	0.00073	442	0.048	1
GO:0019538	protein metabolic process	7175	2811	3.60E-06	0.00076	902	1	1
GO:0034613	cellular protein localization	562	278	7.30E-06	0.0014	31	1	1
GO:0006950	response to stress	3059	1260	7.40E-06	0.0014	571	2.7E-11	7.5E-09
GO:0070727	cellular macromolecule localization	562	278	7.30E-06	0.0014	31	1	1

GO:0006886	intracellular protein transport	554	273	1.10E-05	0.0021	31	1	1
GO:0008610	lipid biosynthetic process	461	230	2.80E-05	0.0052	52	0.94	1
GO:0032774	RNA biosynthetic process	2364	981	3.50E-05	0.0063	380	0.0037	0.19
GO:0006629	lipid metabolic process	1068	476	3.70E-05	0.0063	171	0.045	1
GO:0023052	signaling	1673	714	3.60E-05	0.0063	211	0.92	1
GO:0006351	transcription, DNA-dependent	2362	979	4.10E-05	0.0068	380	0.0036	0.19
GO:0051252	regulation of RNA metabolic process	2211	917	6.70E-05	0.011	374	0.0002	0.018
GO:0044281	small molecule metabolic process	2730	1114	7.60E-05	0.012	313	1	1
GO:0006796	phosphate metabolic process	2994	1214	7.70E-05	0.012	449	0.06	1
GO:0006355	regulation of transcription, DNA-dependent	2208	915	7.40E-05	0.012	374	0.00018	0.017
GO:0006793	phosphorus metabolic process	2995	1214	8.00E-05	0.012	449	0.06	1
GO:0044255	cellular lipid metabolic process	474	231	8.20E-05	0.012	57	0.86	1
GO:0044262	cellular carbohydrate metabolic process	959	425	0.00013	0.019	101	1	1
GO:0044283	small molecule biosynthetic process	708	323	0.00018	0.025	67	1	1
GO:0006811	ion transport	1096	475	0.00026	0.035	163	0.21	1
GO:0006468	protein amino acid phosphorylation	2387	971	0.0003	0.039	401	0.00019	0.018
GO:0008104	protein localization	890	392	0.00034	0.045	52	1	1
GO:0033036	macromolecule localization	997	434	0.00035	0.045	72	1	1
GO:0016310	phosphorylation	2743	1104	0.00036	0.045	427	0.013	0.55
GO:0030001	metal ion transport	616	282	0.00037	0.046	97	0.13	1
GO:0001883	purine nucleoside binding	5398	2248	5.10E-11	6.30E-08	761	3.40E-01	1.00E+00
GO:0001882	nucleoside binding	5400	2249	4.90E-11	6.30E-08	761	3.40E-01	1.00E+00
GO:0030554	adenyl nucleotide binding	5398	2248	5.10E-11	6.30E-08	761	3.40E-01	1.00E+00
GO:0017111	nucleoside-triphosphatase activity	1822	852	4.20E-11	6.30E-08	167	1.00E+00	1.00E+00
GO:0000166	nucleotide binding	6408	2627	8.50E-11	8.50E-08	844	9.40E-01	1.00E+00
GO:0016818	hydrolase activity, acting on acid anhydrides, in phosphorus-containing anhydrides	1958	902	1.20E-10	9.00E-08	183	1.00E+00	1.00E+00
GO:0016462	pyrophosphatase activity	1907	881	1.30E-10	9.00E-08	181	1.00E+00	1.00E+00
GO:0016740	transferase activity	5727	2364	1.60E-10	1.00E-07	875	2.40E-03	1.10E-01
GO:0032559	adenyl ribonucleotide binding	5137	2138	2.00E-10	1.10E-07	702	6.70E-01	1.00E+00
GO:0016817	hydrolase activity, acting on acid anhydrides	1980	907	2.40E-10	1.20E-07	184	1.00E+00	1.00E+00
GO:0005524	ATP binding	5133	2134	2.80E-10	1.30E-07	702	6.60E-01	1.00E+00
GO:0017076	purine nucleotide binding	6079	2489	4.50E-10	1.90E-07	815	8.50E-01	1.00E+00
GO:0032555	purine ribonucleotide binding	5800	2372	1.80E-09	6.40E-07	754	9.70E-01	1.00E+00
GO:0032553	ribonucleotide binding	5800	2372	1.80E-09	6.40E-07	754	9.70E-01	1.00E+00
GO:0003824	catalytic activity	17617	6692	8.60E-09	2.80E-06	2564	1.30E-03	6.50E-02

GO:0005215	transporter activity	2411	1047	5.00E-08	1.50E-05	322	7.60E-01	1.00E+00
GO:0016757	transferase activity, transferring glycosyl groups	747	369	2.90E-07	8.60E-05	152	1.80E-05	1.50E-03
GO:0022892	substrate-specific transporter activity	1632	726	4.70E-07	0.00013	223	0.6	1
GO:0005515	protein binding	4766	1929	6.60E-07	0.00017	566	1	1
GO:0050825	ice binding	1758	770	1.20E-06	0.00029	298	0.00076	0.042
GO:0050824	water binding	1758	770	1.20E-06	0.00029	298	0.00076	0.042
GO:0016773	phosphotransferase activity, alcohol group as acceptor	2858	1193	2.20E-06	0.00048	457	0.0022	0.1
GO:0016887	ATPase activity	785	376	2.20E-06	0.00048	75	1	1
GO:0004871	signal transducer activity	1377	614	2.90E-06	0.00057	201	0.26	1
GO:0060089	molecular transducer activity	1377	614	2.90E-06	0.00057	201	0.26	1
GO:0015075	ion transmembrane transporter activity	1258	565	3.80E-06	0.00072	176	0.47	1
GO:0022857	transmembrane transporter activity	1672	727	5.10E-06	0.00094	234	0.46	1
GO:0016787	hydrolase activity	6078	2396	7.00E-06	0.0012	826	0.74	1
GO:0022891	substrate-specific transmembrane transporter activity	1472	645	8.50E-06	0.0015	214	0.27	1
GO:0004674	protein serine/threonine kinase activity	2248	944	1.40E-05	0.0023	387	0.000045	0.0033
GO:0016301	kinase activity	3006	1230	2.20E-05	0.0036	475	0.0037	0.17
GO:0008324	cation transmembrane transporter activity	996	448	3.30E-05	0.0051	138	0.52	1
GO:0016758	transferase activity, transferring hexosyl groups	542	263	3.50E-05	0.0053	130	6E-08	0.00001
GO:0004672	protein kinase activity	2423	994	0.00011	0.016	404	0.0003	0.018
GO:0042623	ATPase activity, coupled	579	273	0.00011	0.016	56	1	1
GO:0022804	active transmembrane transporter activity	825	370	0.00018	0.025	125	0.19	1
GO:0005623	cell	13914	5581	9.40E-20	6.50E-17	1793	1.00E+00	1.00E+00
GO:0044464	cell part	13914	5581	9.40E-20	6.50E-17	1793	1.00E+00	1.00E+00
GO:0005622	intracellular	9403	3769	7.70E-12	3.60E-09	1103	1.00E+00	1.00E+00
GO:0016020	membrane	5307	2222	1.60E-11	5.50E-09	765	1.50E-01	1.00E+00
GO:0031224	intrinsic to membrane	2183	979	1.30E-09	3.70E-07	324	1.30E-01	1.00E+00
GO:0016021	integral to membrane	2156	967	1.60E-09	3.80E-07	319	1.40E-01	1.00E+00
GO:0044425	membrane part	2684	1174	2.20E-09	4.30E-07	383	3.10E-01	1.00E+00
GO:0044424	intracellular part	7930	3162	4.60E-09	8.00E-07	928	1.00E+00	1.00E+00
GO:0005737	cytoplasm	3593	1497	1.40E-07	2.10E-05	328	1.00E+00	1.00E+00
GO:0043231	intracellular membrane-bounded organelle	4093	1646	1.40E-05	0.0016	517	0.99	1
GO:0043229	intracellular organelle	6224	2443	1.30E-05	0.0016	756	1	1
GO:0043227	membrane-bounded organelle	4127	1658	1.50E-05	0.0016	525	0.98	1
GO:0043226	organelle	6224	2443	1.30E-05	0.0016	756	1	1
GO:0044444	cytoplasmic part	2678	1107	1.80E-05	0.0017	258	1	1
GO:0005739	mitochondrion	354	179	0.00012	0.01	21	1	1
GO:0031090	organelle membrane	456	222	0.00012	0.01	32	1	1

GO:0044429	mitochondrial part	309	159	0.00015	0.012	18	1	1
GO:0031967	organelle envelope	310	159	0.00017	0.013	16	1	1
GO:0031975	envelope	359	179	0.00021	0.015	26	1	1
GO:0031966	mitochondrial membrane	241	128	0.00024	0.016	13	1	1
GO:0005740	mitochondrial envelope	280	143	0.00039	0.026	16	1	1
GO:0010556#	regulation of macromolecule biosynthetic process	3393	1295	2.00E-02	8.70E-01	656	1.90E-15	1.90E-12
GO:0009889#	regulation of biosynthetic process	3393	1295	2.00E-02	8.70E-01	656	1.90E-15	1.90E-12
GO:0031326#	regulation of cellular biosynthetic process	3393	1295	2.00E-02	8.70E-01	656	1.90E-15	1.90E-12
GO:0031323#	regulation of cellular metabolic process	3439	1313	1.90E-02	8.40E-01	663	2.10E-15	1.90E-12
GO:0010468#	regulation of gene expression	3419	1306	1.90E-02	8.30E-01	658	3.70E-15	2.70E-12
GO:0045449#	regulation of transcription	3352	1279	2.20E-02	8.90E-01	646	5.50E-15	3.30E-12
GO:0019219#	regulation of nucleobase, nucleoside, nucleotide and nucleic acid metabolic process	3370	1287	2.00E-02	8.60E-01	646	1.40E-14	6.10E-12
GO:0051171#	regulation of nitrogen compound metabolic process	3370	1287	2.00E-02	8.60E-01	646	1.40E-14	6.10E-12
GO:0080090#	regulation of primary metabolic process	3595	1372	1.70E-02	7.90E-01	666	1.60E-12	6.20E-10
GO:0019222#	regulation of metabolic process	3667	1402	1.40E-02	7.30E-01	675	3.10E-12	1.10E-09
GO:0060255	regulation of macromolecule metabolic process	3632	1388	1.50E-02	7.30E-01	668	4.60E-12	1.50E-09
GO:0006350	transcription	3730	1396	6.80E-02	1.00E+00	678	2.00E-11	5.90E-09
GO:0006950	response to stress	3059	1260	7.40E-06	1.40E-03	571	2.70E-11	7.50E-09
GO:0050896	response to stimulus	3421	1429	1.80E-07	8.20E-05	627	3.90E-11	1.00E-08
GO:0055114	oxidation reduction	2743	937	9.00E-01	1.00E+00	514	1.90E-10	4.40E-08
GO:0006979	response to oxidative stress	315	73	1.00E+00	1.00E+00	96	2.10E-10	4.60E-08
GO:0042221	response to chemical stimulus	626	215	7.10E-01	1.00E+00	156	3.20E-10	6.60E-08
GO:0065007	biological regulation	6272	2501	3.30E-07	1.20E-04	1054	3.70E-10	7.30E-08
GO:0050794	regulation of cellular process	4274	1667	8.50E-04	9.70E-02	747	9.20E-10	1.60E-07
GO:0012501	programmed cell death	168	67	2.50E-01	1.00E+00	62	9.50E-10	1.60E-07
GO:0006915	apoptosis	168	67	2.50E-01	1.00E+00	62	9.50E-10	1.60E-07
GO:0008219	cell death	201	77	3.30E-01	1.00E+00	69	1.40E-09	2.10E-07
GO:0016265	death	201	77	3.30E-01	1.00E+00	69	1.40E-09	2.10E-07
GO:0071554	cell wall organization or biogenesis	270	69	1.00E+00	1.00E+00	80	1.80E-08	2.60E-06
GO:0006952	defense response	608	259	1.00E-02	6.10E-01	143	3.70E-08	5.30E-06
GO:0050789	regulation of biological process	4504	1756	6.50E-04	7.90E-02	761	8.90E-08	1.20E-05
GO:0070882	cellular cell wall organization or biogenesis	37	9	8.90E-01	1.00E+00	20	6.70E-06	0.00088
GO:0071555	cell wall organization	172	35	1.00E+00	1.00E+00	50	1.20E-05	0.0015
GO:0009856	pollination	56	20	5.50E-01	1.00E+00	24	1.80E-05	0.0021

GO:0048544	recognition of pollen	56	20	5.50E-01	1.00E+00	24	1.80E-05	0.0021
GO:0009875	pollen-pistil interaction	56	20	5.50E-01	1.00E+00	24	1.80E-05	0.0021
GO:0042546	cell wall biogenesis	26	5	9.40E-01	1.00E+00	15	5.30E-05	0.0059
GO:0006030	chitin metabolic process	43	12	8.20E-01	1.00E+00	19	9.60E-05	0.01
GO:0006032	chitin catabolic process	43	12	8.20E-01	1.00E+00	19	9.60E-05	0.01
GO:0006026	aminoglycan catabolic process	44	13	0.77	1	19	0.00012	0.012
GO:0008037	cell recognition	70	24	0.61	1	25	0.00013	0.013
GO:0006355	regulation of transcription, DNA-dependent	2208	915	0.000074	0.012	374	0.00018	0.017
GO:0006468	protein amino acid phosphorylation	2387	971	0.0003	0.039	401	0.00019	0.018
GO:0000003	reproduction	181	48	0.98	1	47	0.0002	0.018
GO:0032501	multicellular organismal process	1983	868	0.00000027	0.0001	339	0.0002	0.018
GO:0051252	regulation of RNA metabolic process	2211	917	0.000067	0.011	374	0.0002	0.018
GO:0009698	phenylpropanoid metabolic process	18	0			11	0.00037	0.027
GO:0006022	aminoglycan metabolic process	49	15	0.74	1	19	0.00036	0.027
GO:0046274	lignin catabolic process	18	0			11	0.00037	0.027
GO:0044092	negative regulation of molecular function	66	27	0.31	1	23	0.00033	0.027
GO:0022414	reproductive process	85	28	0.68	1	27	0.00036	0.027
GO:0009808	lignin metabolic process	18	0			11	0.00037	0.027
GO:0043086	negative regulation of catalytic activity	66	27	0.31	1	23	0.00033	0.027
GO:0046271	phenylpropanoid catabolic process	18	0			11	0.00037	0.027
GO:0008152	metabolic process	20231	7591	0.00000017	0.000082	2938	0.00049	0.035
GO:0042219	cellular amino acid derivative catabolic process	19	0			11	0.00051	0.036
GO:0009628	response to abiotic stimulus	1856	819	0.00000023	0.000092	314	0.00062	0.043
GO:0000272	polysaccharide catabolic process	75	22	0.83	1	24	0.00067	0.045
GO:0001659	temperature homeostasis	1758	770	0.0000012	0.0003	298	0.00076	0.046
GO:0050826	response to freezing	1758	770	0.0000012	0.0003	298	0.00076	0.046
GO:0009409	response to cold	1758	770	0.0000012	0.0003	298	0.00076	0.046
GO:0042309	homoiothermy	1758	770	0.0000012	0.0003	298	0.00076	0.046
GO:0048871	multicellular organismal homeostasis	1758	770	0.0000012	0.0003	298	0.00076	0.046
GO:0065008	regulation of biological quality	2139	943	0.000000031	0.000021	356	0.00075	0.046
GO:0020037	heme binding	876	249	1.00E+00	1.00E+00	256	2.70E-23	5.80E-20
GO:0046906	tetrapyrrole binding	880	249	1.00E+00	1.00E+00	256	4.60E-23	5.80E-20
GO:0004497	monooxygenase activity	553	178	9.00E-01	1.00E+00	181	5.90E-21	5.00E-18
GO:0009055	electron carrier activity	914	317	7.00E-01	1.00E+00	254	1.10E-20	6.90E-18
GO:0005506	iron ion binding	1187	384	9.60E-01	1.00E+00	306	1.50E-20	7.50E-18

GO:0030528	transcription regulator activity	2233	818	2.80E-01	1.00E+00	446	1.90E-12	7.90E-10
GO:0003700	transcription factor activity	1390	527	1.30E-01	1.00E+00	302	3.90E-12	1.40E-09
GO:0016491	oxidoreductase activity	3266	1155	6.50E-01	1.00E+00	608	8.40E-12	2.70E-09
GO:0016684	oxidoreductase activity, acting on peroxide as acceptor	329	78	1.00E+00	1.00E+00	102	2.60E-11	6.60E-09
GO:0004601	peroxidase activity	329	78	1.00E+00	1.00E+00	102	2.60E-11	6.60E-09
GO:0005507	copper ion binding	194	58	9.00E-01	1.00E+00	72	3.60E-11	8.40E-09
GO:0016209	antioxidant activity	374	99	1.00E+00	1.00E+00	108	2.20E-10	4.60E-08
GO:0004857	enzyme inhibitor activity	236	66	9.70E-01	1.00E+00	72	3.40E-08	6.70E-06
GO:0030599	pectinesterase activity	151	27	1.00E+00	1.00E+00	53	5.80E-08	1.00E-05
GO:0016758	transferase activity, transferring hexosyl groups	542	263	3.50E-05	5.30E-03	130	6.00E-08	1.00E-05
GO:0043565	sequence-specific DNA binding	985	384	7.90E-02	1.00E+00	203	3.70E-07	5.90E-05
GO:0046872	metal ion binding	6652	2209	1.00E+00	1.00E+00	1072	4.60E-07	6.90E-05
GO:0043167	ion binding	6665	2215	1.00E+00	1.00E+00	1073	5.40E-07	7.20E-05
GO:0043169	cation binding	6664	2215	1.00E+00	1.00E+00	1073	5.20E-07	7.20E-05
GO:0008234	cysteine-type peptidase activity	332	74	1.00E+00	1.00E+00	86	7.30E-07	9.30E-05
GO:0030145	manganese ion binding	90	21	9.70E-01	1.00E+00	35	1.50E-06	0.00018
GO:0004091	carboxylesterase activity	280	81	9.60E-01	1.00E+00	74	2.30E-06	0.00026
GO:0004499	flavin-containing monooxygenase activity	73	22	7.90E-01	1.00E+00	30	3.40E-06	0.00038
GO:0008417	fucosyltransferase activity	20	0			15	5.60E-06	0.00055
GO:0008107	galactoside 2-alpha-L-fucosyltransferase activity	20	0			15	5.60E-06	0.00055
GO:0031127	alpha(1,2)-fucosyltransferase activity	20	0			15	5.60E-06	0.00055
GO:0005529	sugar binding	150	60	2.50E-01	1.00E+00	46	8.30E-06	0.00078
GO:0005509	calcium ion binding	534	202	2.60E-01	1.00E+00	117	1.10E-05	0.00097
GO:0016709	oxidoreductase activity, acting on paired donors, with incorporation or reduction of molecular oxygen, NADH or NADPH as one donor, and incorporation of one atom of oxygen	79	26	6.80E-01	1.00E+00	30	1.20E-05	0.001
GO:0016757	transferase activity, transferring glycosyl groups	747	369	2.90E-07	8.60E-05	152	1.80E-05	0.0015
GO:0045735	nutrient reservoir activity	146	65	8.30E-02	1.00E+00	44	1.80E-05	0.0015
GO:0016747	transferase activity, transferring acyl groups other than amino-acyl groups	357	148	7.30E-02	1.00E+00	84	2.20E-05	0.0017
GO:0016682	oxidoreductase activity, acting on diphenols and related substances as donors,	25	5	9.30E-01	1.00E+00	15	3.80E-05	0.0029

	oxygen as acceptor							
GO:0004674	protein serine/threonine kinase activity	2248	944	1.40E-05	2.30E-03	387	4.50E-05	0.0033
GO:0030246	carbohydrate binding	271	106	2.30E-01	1.00E+00	66	6.60E-05	0.0048
GO:0004568	chitinase activity	43	12	8.20E-01	1.00E+00	19	9.60E-05	0.0068
GO:0016798	hydrolase activity, acting on glycosyl bonds	821	298	0.43	1	159	0.0001	0.0071
GO:0004197	cysteine-type endopeptidase activity	164	58	0.56	1	44	0.00018	0.012
GO:0008171	O-methyltransferase activity	64	17	0.89	1	23	0.00023	0.015
GO:0050660	FAD binding	265	111	0.091	1	62	0.00027	0.017
GO:0004672	protein kinase activity	2423	994	0.00011	0.016	404	0.0003	0.018
GO:0004553	hydrolase activity, hydrolyzing O-glycosyl compounds	767	282	0.36	1	146	0.00037	0.022
GO:0008471	laccase activity	18	0			11	0.00037	0.022
GO:0016746	transferase activity, transferring acyl groups	437	182	0.047	1	90	0.0006	0.035
GO:0050825	ice binding	1758	770	0.0000012	0.00029	298	0.00076	0.042
GO:0050824	water binding	1758	770	0.0000012	0.00029	298	0.00076	0.042
GO:0050661	NADP or NADPH binding	121	51	0.18	1	33	0.00084	0.045
GO:0005576	extracellular region	583	174	9.90E-01	1.00E+00	166	9.80E-15	6.90E-12
GO:0048046	apoplast	126	31	9.80E-01	1.00E+00	54	1.60E-10	5.70E-08
GO:0030312	external encapsulating structure	219	64	9.30E-01	1.00E+00	58	2.50E-05	0.0059
GO:0005618	cell wall	172	45	9.80E-01	1.00E+00	48	4.10E-05	0.0072

*Top 10 enriched GO terms in non-ePAV set.

#Top 10 enriched GO terms in ePAV set.

Supplementary Table S2.ePAV validation by genomic PCR of the previous identified 2M region (on chr6) absence in MO17.

Probe Name	IDP5870	IDP7015	IDP1993	IDP5958	IDP4398	IDP5013	IDP5899	IDP4178	IDP2010	IDP7930	IDP7839	IDP6876	IDP8422
Region (Mb)	12.711-713	13.114-115	13.188-189	14.128-129	14.303-304	14.484-485	8.136-14.765	14.761-765	14.929-931	14.968-972	14.985-986	8.136-14.765	15.025-027
150	Y	Y	Y	Y	Y	Y	Y	Y	Y	Y	Y	Y	Y
177	Y	Y	Y	Y	Y	Y	Y	Y	Y	Y	Y	Y	Y
238	Y	Y	Y	Y	Y	Y	Y	Y	Y	Y	Y	Y	Y
1462	Y	Y	Y	Y	Y	Y	Y	Y	Y	Y	Y	Y	Y
5213	Y	Y	Y	Y	Y	Y	Y	Y	Y	Y	Y	Y	Y
5237	Y	Y	Y	Y	Y	Y	Y	Y	Y	Y	Y	Y	Y
7381	Y	Y	Y	Y	Y	Y	Y	Y	Y	Y	Y	Y	Y
8902	Y	Y	Y	Y	Y	Y	Y	Y	Y	Y	Y	Y	Y
9642	Y	Y	Y	Y	Y	Y	Y	Y	Y	Y	Y	Y	Y
81162	Y	Y	Y	Y	Y	Y	Y	Y	Y	Y	Y	Y	Y
526018	Y	Y	Y	Y	Y	Y	Y	Y	Y	Y	Y	Y	Y
05W002	Y	Y	Y	Y	Y	Y	Y	Y	Y	Y	Y	Y	Y
05WN230	Y	Y	Y	Y	Y	Y	Y	Y	Y	Y	Y	Y	Y
18-599	Y	Y	Y	Y	Y	Y	Y	Y	Y	Y	Y	Y	Y
303WX	Y	Y	Y	Y	Y	Y	Y	Y	Y	Y	Y	Y	Y
4F1	N	N	N	N	N	N	N	N	N	N	N	N	N
7884-4HT	Y	Y	Y	Y	Y	Y	Y	Y	Y	Y	Y	Y	Y
835A	Y	Y	Y	Y	Y	Y	Y	Y	Y	Y	Y	Y	Y
975-12	Y	Y	Y	Y	Y	Y	Y	Y	Y	Y	Y	N	Y
B11	Y	Y	Y	Y	Y	Y	Y	Y	Y	Y	Y	Y	Y
B110	Y	Y	Y	Y	Y	Y	Y	Y	Y	Y	Y	Y	Y
B114	Y	Y	Y	Y	Y	Y	Y	Y	Y	Y	Y	Y	Y
B73	Y	Y	Y	Y	Y	Y	Y	Y	Y	Y	Y	Y	Y
B77	Y	Y	Y	Y	Y	Y	Y	Y	Y	Y	Y	Y	Y
BS16	Y	Y	Y	Y	Y	Y	Y	Y	Y	Y	Y	Y	Y
BY4839	Y	Y	Y	Y	Y	Y	Y	Y	Y	Y	Y	Y	Y
BY4944	Y	Y	Y	Y	Y	Y	Y	Y	Y	Y	Y	Y	Y

BY4960	Y	Y	Y	Y	Y	Y	Y	Y	Y	Y	Y	Y	Y
BY804	Y	Y	Y	Y	Y	Y	Y	Y	Y	Y	Y	Y	Y
BY807	Y	Y	Y	Y	Y	Y	Y	Y	Y	Y	Y	Y	Y
BY809	Y	Y	Y	Y	Y	Y	Y	Y	Y	Y	Y	Y	Y
BY813	Y	Y	Y	Y	Y	Y	Y	Y	Y	Y	Y	Y	Y
BY815	Y	Y	Y	Y	Y	Y	Y	Y	Y	Y	Y	Y	Y
BY855	Y	Y	Y	Y	Y	Y	Y	Y	Y	Y	Y	Y	Y
CA47	Y	Y	Y	Y	Y	Y	Y	Y	Y	Y	Y	Y	Y
CHANG3	N	N	N	N	N	N	N	N	N	N	N	N	N
CHENG698	Y	Y	Y	Y	Y	Y	Y	Y	Y	Y	Y	N	Y
CI7	Y	Y	Y	N	Y	Y	Y	Y	Y	Y	Y	Y	Y
CIMBL10	Y	Y	Y	Y	Y	Y	N	Y	Y	Y	Y	Y	Y
CIMBL101	Y	Y	Y	Y	Y	Y	Y	Y	Y	Y	Y	Y	Y
CIMBL102	Y	Y	Y	Y	Y	Y	Y	Y	Y	Y	Y	Y	Y
CIMBL105	Y	Y	Y	Y	Y	Y	N	Y	Y	Y	Y	Y	Y
CIMBL106	Y	Y	Y	N	Y	Y	Y	Y	Y	Y	Y	Y	Y
CIMBL108	Y	Y	Y	N	Y	Y	N	Y	Y	Y	Y	Y	Y
CIMBL11	Y	Y	Y	Y	Y	Y	Y	N	Y	Y	Y	Y	Y
CIMBL111	Y	Y	Y	Y	Y	Y	Y	Y	Y	Y	Y	Y	Y
CIMBL113	Y	Y	Y	N	Y	Y	Y	Y	Y	Y	Y	Y	Y
CIMBL114	Y	Y	Y	Y	Y	Y	Y	Y	Y	Y	Y	Y	Y
CIMBL115	Y	Y	Y	Y	Y	Y	Y	Y	Y	Y	Y	Y	Y
CIMBL116	Y	Y	Y	Y	Y	Y	Y	Y	Y	Y	Y	Y	Y
CIMBL12	Y	Y	Y	Y	Y	Y	Y	N	Y	Y	Y	Y	Y
CIMBL120	Y	Y	Y	Y	Y	Y	Y	Y	Y	Y	Y	Y	Y
CIMBL121	Y	Y	Y	Y	Y	Y	Y	Y	Y	Y	Y	Y	Y
CIMBL122	Y	Y	Y	Y	Y	Y	Y	Y	Y	Y	Y	Y	Y
CIMBL123	Y	Y	Y	Y	Y	Y	Y	Y	Y	Y	Y	Y	Y
CIMBL124	Y	Y	Y	Y	Y	Y	Y	Y	Y	Y	Y	Y	Y
CIMBL127	Y	Y	Y	Y	Y	Y	Y	Y	Y	Y	Y	Y	Y

CIMBL129	Y	Y	Y	Y	Y	Y	Y	Y	Y	Y	Y	Y	Y
CIMBL13	Y	Y	Y	Y	Y	Y	Y	Y	Y	Y	Y	Y	Y
CIMBL133	Y	Y	Y	Y	Y	Y	Y	Y	Y	Y	Y	Y	Y
CIMBL142	Y	Y	Y	Y	Y	Y	N	Y	Y	Y	Y	Y	Y
CIMBL147	Y	Y	Y	Y	Y	Y	Y	Y	Y	Y	Y	Y	Y
CIMBL15	Y	Y	Y	Y	Y	Y	Y	Y	Y	Y	Y	Y	Y
CIMBL157	Y	Y	Y	Y	Y	Y	Y	Y	Y	Y	Y	Y	Y
CIMBL16	Y	Y	Y	Y	Y	Y	Y	Y	Y	Y	Y	Y	Y
CIMBL17	Y	Y	Y	Y	Y	Y	Y	Y	Y	Y	Y	Y	Y
CIMBL18	Y	Y	Y	Y	Y	Y	Y	Y	Y	Y	Y	Y	Y
CIMBL19	Y	Y	Y	Y	Y	Y	Y	Y	Y	Y	Y	Y	Y
CIMBL2	N	N	N	N	N	N	N	N	N	N	N	N	N
CIMBL21	Y	Y	Y	Y	Y	Y	Y	Y	Y	Y	Y	Y	Y
CIMBL22	Y	Y	Y	Y	Y	Y	Y	Y	N	Y	Y	Y	Y
CIMBL25	Y	Y	Y	Y	Y	Y	Y	Y	Y	Y	Y	Y	Y
CIMBL27	Y	Y	Y	Y	Y	Y	Y	Y	Y	Y	Y	Y	Y
CIMBL28	Y	Y	Y	Y	Y	Y	Y	Y	Y	Y	Y	Y	Y
CIMBL29	Y	Y	Y	Y	Y	Y	Y	Y	Y	Y	Y	Y	Y
CIMBL32	Y	Y	Y	Y	Y	Y	Y	Y	Y	Y	Y	Y	Y
CIMBL38	Y	Y	Y	Y	Y	Y	Y	Y	Y	Y	Y	Y	Y
CIMBL4	Y	Y	Y	Y	Y	Y	Y	Y	Y	Y	Y	Y	Y
CIMBL40	Y	Y	Y	Y	Y	Y	Y	Y	Y	Y	Y	Y	Y
CIMBL42	Y	Y	Y	Y	Y	Y	Y	Y	Y	Y	Y	Y	Y
CIMBL43	Y	Y	Y	Y	Y	Y	Y	Y	N	Y	Y	Y	Y
CIMBL48	Y	Y	Y	Y	Y	Y	Y	Y	Y	Y	Y	Y	Y
CIMBL49	Y	Y	Y	Y	Y	Y	Y	Y	Y	Y	Y	Y	Y
CIMBL5	Y	Y	Y	Y	Y	Y	Y	Y	Y	Y	Y	Y	Y
CIMBL50	Y	Y	Y	Y	Y	Y	Y	Y	Y	Y	Y	Y	Y
CIMBL51	Y	Y	Y	Y	Y	Y	Y	Y	Y	Y	Y	Y	Y
CIMBL52	Y	Y	Y	Y	Y	Y	Y	Y	Y	Y	Y	Y	Y

CIMBL54	Y	Y	Y	Y	Y	Y	Y	Y	Y	Y	Y	Y	Y
CIMBL55	Y	Y	Y	Y	Y	Y	Y	Y	Y	Y	Y	Y	Y
CIMBL56	Y	Y	Y	Y	Y	Y	Y	Y	Y	Y	Y	Y	Y
CIMBL58	Y	Y	Y	Y	Y	Y	Y	Y	Y	Y	Y	Y	Y
CIMBL62	Y	Y	Y	Y	Y	Y	Y	Y	Y	Y	Y	Y	Y
CIMBL63	Y	Y	Y	Y	Y	Y	Y	Y	Y	Y	Y	Y	Y
CIMBL66	Y	Y	Y	Y	Y	Y	N	Y	Y	Y	Y	Y	Y
CIMBL68	Y	Y	Y	Y	Y	Y	N	Y	Y	Y	Y	Y	Y
CIMBL69	Y	Y	Y	Y	Y	Y	N	Y	Y	Y	Y	Y	Y
CIMBL7	Y	Y	Y	Y	Y	Y	N	Y	Y	Y	Y	Y	Y
CIMBL70	Y	Y	Y	Y	Y	Y	Y	Y	Y	Y	Y	Y	Y
CIMBL71	Y	Y	Y	Y	Y	Y	Y	N	Y	Y	Y	Y	Y
CIMBL74	Y	Y	Y	Y	Y	Y	Y	Y	Y	Y	Y	Y	Y
CIMBL75	Y	Y	Y	Y	Y	Y	Y	Y	Y	Y	Y	Y	Y
CIMBL81	Y	Y	Y	Y	Y	Y	Y	Y	Y	Y	Y	Y	Y
CIMBL82	Y	Y	Y	Y	Y	Y	Y	Y	Y	Y	Y	Y	Y
CIMBL83	Y	Y	Y	Y	Y	Y	Y	N	Y	Y	Y	Y	Y
CIMBL84	Y	Y	Y	Y	Y	Y	Y	Y	Y	Y	Y	Y	Y
CIMBL87	Y	Y	Y	Y	Y	Y	Y	Y	Y	Y	Y	Y	Y
CIMBL88	Y	Y	Y	Y	Y	Y	Y	Y	Y	Y	Y	Y	Y
CIMBL9	Y	Y	Y	Y	Y	Y	N	Y	Y	Y	Y	Y	Y
CIMBL90	Y	Y	Y	Y	Y	Y	Y	Y	Y	Y	Y	Y	Y
CIMBL92	Y	Y	Y	Y	Y	Y	Y	Y	Y	Y	Y	Y	Y
CIMBL93	Y	Y	Y	Y	Y	Y	Y	Y	Y	Y	Y	Y	Y
CIMBL94	Y	Y	Y	Y	Y	Y	Y	Y	Y	Y	Y	Y	Y
CIMBL95	Y	Y	Y	Y	Y	Y	Y	N	Y	Y	Y	Y	Y
CIMBL96	Y	Y	Y	Y	Y	Y	Y	Y	Y	Y	Y	Y	Y
CIMBL98	Y	Y	Y	Y	Y	Y	Y	Y	Y	Y	Y	Y	Y
CML114	Y	Y	Y	Y	Y	Y	N	Y	Y	Y	Y	Y	Y
CML115	Y	Y	Y	Y	Y	Y	Y	Y	Y	Y	Y	Y	Y

CML116	Y	Y	Y	Y	Y	Y	Y	Y	Y	Y	Y	Y	Y
CML118	Y	Y	Y	Y	Y	Y	Y	Y	Y	Y	Y	Y	Y
CML121	Y	Y	Y	N	Y	Y	Y	Y	Y	Y	Y	Y	Y
CML122	Y	Y	Y	Y	Y	Y	Y	Y	Y	Y	Y	Y	Y
CML130	Y	Y	Y	Y	Y	Y	Y	Y	Y	Y	Y	Y	Y
CML134	Y	Y	Y	Y	Y	Y	Y	Y	Y	Y	Y	Y	Y
CML139	Y	Y	Y	Y	Y	Y	Y	Y	Y	Y	Y	Y	Y
CML162	Y	Y	Y	Y	Y	Y	Y	Y	N	Y	Y	Y	Y
CML163	Y	Y	Y	N	Y	Y	N	Y	Y	Y	Y	Y	Y
CML165	Y	Y	Y	Y	Y	Y	N	Y	Y	Y	Y	Y	Y
CML169	Y	Y	Y	Y	Y	Y	Y	Y	Y	Y	Y	Y	Y
CML170	Y	Y	Y	Y	Y	Y	Y	Y	Y	Y	Y	Y	Y
CML171	Y	Y	Y	Y	Y	Y	Y	Y	Y	Y	Y	Y	Y
CML172	Y	Y	Y	Y	Y	Y	Y	Y	Y	Y	Y	Y	Y
CML189	Y	Y	Y	Y	Y	Y	Y	Y	Y	Y	Y	Y	Y
CML191	Y	Y	Y	N	Y	Y	Y	Y	Y	Y	Y	Y	Y
CML20	Y	Y	Y	Y	Y	Y	Y	Y	Y	Y	Y	Y	Y
CML290	Y	Y	Y	Y	Y	Y	N	Y	Y	Y	Y	Y	Y
CML298	Y	Y	Y	Y	Y	Y	Y	Y	Y	Y	Y	Y	Y
CML304	Y	Y	Y	Y	Y	Y	Y	Y	Y	Y	Y	Y	Y
CML31	Y	Y	Y	Y	Y	Y	Y	Y	Y	Y	Y	Y	Y
CML32	Y	Y	Y	Y	Y	Y	Y	Y	Y	Y	Y	Y	Y
CML323	Y	Y	Y	Y	Y	Y	Y	Y	Y	Y	Y	Y	Y
CML324	Y	Y	Y	Y	Y	Y	Y	Y	Y	Y	Y	Y	Y
CML327	Y	Y	Y	Y	Y	Y	Y	Y	Y	Y	Y	Y	Y
CML338	Y	Y	Y	N	Y	Y	Y	Y	Y	Y	Y	Y	Y
CML361	Y	Y	Y	Y	Y	Y	N	Y	Y	Y	Y	Y	Y
CML411	Y	Y	Y	Y	Y	Y	Y	Y	Y	Y	Y	Y	Y
CML415	Y	Y	Y	Y	Y	Y	Y	Y	Y	Y	Y	Y	Y
CML422	Y	Y	Y	N	Y	Y	Y	Y	Y	Y	Y	Y	Y

CML423	Y	Y	Y	Y	Y	Y	Y	Y	Y	Y	Y	Y	Y
CML426	Y	Y	Y	Y	Y	Y	Y	Y	N	Y	Y	Y	Y
CML431	Y	Y	Y	Y	Y	Y	N	Y	Y	Y	Y	Y	Y
CML432	Y	Y	Y	Y	Y	Y	Y	Y	Y	Y	Y	Y	Y
CML433	Y	Y	Y	Y	Y	Y	Y	Y	N	Y	Y	Y	Y
CML454	Y	Y	Y	Y	Y	Y	Y	Y	Y	Y	Y	Y	Y
CML470	Y	Y	Y	Y	Y	Y	Y	Y	N	Y	Y	Y	Y
CML479	Y	Y	Y	Y	Y	Y	N	Y	Y	Y	Y	Y	Y
CML480	Y	Y	Y	Y	Y	Y	N	Y	Y	Y	Y	Y	Y
CML486	Y	Y	Y	Y	Y	Y	Y	Y	Y	Y	Y	Y	Y
CML493	Y	Y	Y	N	Y	Y	Y	Y	Y	Y	Y	Y	Y
CML496	Y	Y	Y	Y	Y	Y	N	Y	Y	Y	Y	Y	Y
CML50	Y	Y	Y	Y	Y	Y	Y	Y	Y	Y	Y	Y	Y
CML69	Y	Y	Y	Y	Y	Y	Y	Y	Y	Y	Y	Y	Y
D863F	Y	Y	Y	Y	Y	Y	Y	Y	Y	Y	Y	Y	Y
DAN340	Y	Y	Y	Y	Y	Y	Y	Y	Y	Y	Y	Y	Y
DAN360	Y	Y	Y	Y	Y	Y	Y	Y	Y	Y	Y	Y	Y
DAN599	Y	Y	Y	Y	Y	Y	Y	Y	Y	Y	Y	Y	Y
DH3732	Y	Y	Y	Y	Y	Y	Y	Y	Y	Y	Y	Y	Y
DONG237	N	N	N	N	N	N	N	N	N	N	N	N	N
DONG46	Y	Y	Y	Y	Y	Y	Y	Y	Y	Y	Y	Y	Y
EN25	Y	Y	Y	Y	Y	Y	Y	Y	Y	Y	Y	Y	Y
ES40	Y	Y	Y	Y	Y	Y	Y	Y	Y	Y	Y	Y	Y
FCD0602	Y	Y	Y	Y	Y	Y	Y	Y	Y	Y	Y	Y	Y
GEMS13	Y	Y	Y	Y	Y	Y	N	Y	Y	Y	Y	Y	Y
GEMS14	N	Y	Y	Y	Y	Y	N	Y	Y	Y	Y	Y	Y
GEMS17	Y	Y	Y	Y	Y	Y	Y	Y	Y	Y	Y	Y	Y
GEMS18	Y	Y	Y	Y	Y	Y	Y	Y	Y	Y	Y	Y	Y
GEMS19	Y	Y	Y	Y	Y	Y	Y	Y	Y	Y	Y	Y	Y
GEMS20	Y	Y	Y	Y	Y	Y	Y	Y	Y	Y	Y	Y	Y

GEMS21	Y	Y	Y	Y	Y	Y	Y	Y	Y	Y	Y	Y	Y
GEMS23	Y	Y	Y	Y	Y	Y	Y	Y	Y	Y	Y	Y	Y
GEMS29	N	Y	Y	Y	Y	Y	N	Y	Y	Y	Y	Y	Y
GEMS31	Y	Y	Y	Y	Y	Y	N	Y	Y	Y	Y	Y	Y
GEMS32	Y	Y	Y	Y	Y	Y	N	Y	Y	Y	Y	Y	Y
GEMS33	Y	Y	Y	Y	Y	Y	N	Y	Y	Y	Y	Y	Y
GEMS35	Y	Y	Y	Y	Y	Y	Y	Y	Y	Y	Y	Y	Y
GEMS36	Y	Y	Y	Y	Y	Y	Y	Y	Y	Y	Y	Y	Y
GEMS37	Y	Y	Y	Y	Y	Y	Y	Y	Y	Y	Y	Y	Y
GEMS39	Y	Y	Y	Y	Y	Y	Y	Y	Y	Y	Y	Y	Y
GEMS40	Y	Y	Y	Y	Y	Y	Y	N	Y	Y	Y	Y	Y
GEMS41	N	N	N	N	N	N	N	N	N	N	N	N	N
GEMS42	Y	Y	Y	Y	Y	Y	N	Y	Y	Y	Y	Y	Y
GEMS46	Y	Y	Y	Y	Y	Y	Y	Y	Y	Y	Y	Y	Y
GEMS48	Y	Y	Y	Y	Y	Y	N	Y	Y	Y	Y	Y	Y
GEMS49	Y	Y	Y	Y	Y	Y	Y	Y	Y	Y	Y	Y	Y
GEMS50	Y	Y	Y	Y	Y	Y	Y	Y	Y	Y	Y	Y	Y
GEMS51	Y	Y	Y	Y	Y	Y	Y	Y	Y	Y	Y	Y	Y
GEMS55	N	Y	Y	Y	Y	Y	N	Y	Y	Y	Y	Y	Y
GEMS58	N	N	N	N	N	N	N	N	N	N	N	N	N
GEMS59	N	N	N	N	N	N	N	N	N	N	N	N	N
GEMS6	Y	Y	Y	Y	Y	Y	N	Y	Y	Y	Y	Y	Y
GEMS60	Y	Y	Y	Y	Y	Y	Y	Y	Y	Y	Y	Y	Y
GEMS61	Y	Y	Y	Y	Y	Y	Y	Y	Y	Y	Y	Y	Y
GEMS62	Y	Y	Y	Y	Y	Y	Y	Y	Y	Y	Y	Y	Y
GEMS63	Y	Y	Y	Y	Y	Y	Y	Y	Y	Y	Y	Y	Y
GEMS65	Y	Y	Y	Y	Y	Y	Y	Y	Y	Y	Y	Y	Y
GEMS66	Y	Y	Y	Y	Y	Y	N	Y	Y	Y	Y	Y	Y
GEMS9	Y	Y	Y	Y	Y	Y	N	Y	Y	Y	Y	Y	Y
GY1007	N	N	N	N	N	N	N	N	N	N	N	N	N

GY1032	N	N	N	N	N	N	N	N	N	N	N	N	N
GY386	Y	Y	Y	Y	Y	Y	Y	Y	Y	Y	Y	Y	Y
GY462	Y	Y	Y	Y	Y	Y	Y	Y	Y	Y	Y	Y	Y
GY798	Y	Y	Y	Y	Y	Y	Y	Y	Y	Y	Y	Y	Y
GY923	Y	Y	Y	Y	Y	Y	Y	Y	Y	Y	Y	Y	Y
HYS	Y	Y	Y	Y	Y	Y	Y	Y	Y	Y	Y	Y	Y
HZS	Y	Y	Y	Y	Y	Y	Y	Y	Y	Y	Y	Y	Y
IRF314	Y	Y	Y	Y	Y	Y	Y	Y	Y	Y	Y	Y	Y
J4112	Y	Y	Y	Y	Y	Y	Y	Y	Y	Y	Y	Y	Y
JH59	Y	Y	Y	Y	Y	Y	N	Y	Y	Y	Y	Y	Y
JH96C	Y	Y	Y	Y	Y	Y	N	Y	Y	Y	Y	Y	Y
JI842	N	N	N	N	N	N	N	N	N	N	N	N	N
JI846	N	N	N	N	N	N	N	N	N	N	N	N	N
JI853	Y	Y	Y	Y	Y	Y	Y	Y	Y	Y	Y	Y	Y
JIAO51	Y	Y	Y	Y	Y	Y	Y	Y	Y	Y	Y	Y	Y
JY01	Y	Y	Y	Y	Y	Y	Y	Y	Y	Y	Y	Y	Y
K12	Y	Y	Y	Y	Y	Y	Y	Y	Y	Y	Y	Y	Y
K22	Y	Y	Y	Y	Y	Y	Y	Y	Y	Y	Y	Y	Y
LG001	Y	Y	Y	Y	Y	Y	N	Y	Y	Y	Y	Y	Y
LIAO138	Y	Y	Y	Y	Y	Y	Y	Y	Y	Y	Y	Y	Y
LIAO159	Y	Y	Y	Y	Y	Y	Y	Y	Y	Y	Y	Y	Y
LIAO5114	Y	Y	Y	Y	Y	Y	Y	Y	Y	Y	Y	Y	Y
LIAO5262	Y	Y	Y	Y	Y	Y	Y	Y	Y	Y	Y	Y	Y
LIAO5263	Y	Y	Y	Y	Y	Y	Y	Y	Y	Y	Y	Y	Y
LV28	Y	Y	Y	N	Y	Y	Y	Y	Y	Y	Y	Y	Y
LX9801	Y	Y	Y	Y	Y	Y	Y	Y	Y	Y	Y	Y	Y
LXN	Y	Y	Y	Y	Y	Y	Y	Y	Y	Y	Y	N	Y
LY042	Y	Y	Y	Y	Y	Y	Y	Y	Y	Y	Y	Y	Y
M153	Y	Y	Y	Y	Y	Y	Y	Y	Y	Y	Y	Y	Y
M97	Y	Y	Y	Y	Y	Y	Y	Y	Y	Y	Y	Y	Y

MO17	N	N	N	N	N	N	N	N	N	N	N	N	N
NAN21-3	Y	Y	Y	Y	Y	Y	Y	Y	Y	Y	Y	Y	Y
P178	Y	Y	Y	Y	Y	Y	Y	Y	Y	Y	Y	Y	Y
Q1261	Y	Y	Y	Y	Y	Y	Y	Y	Y	Y	Y	Y	Y
QI205	Y	Y	Y	Y	Y	Y	Y	Y	N	Y	Y	Y	Y
R15	Y	Y	Y	Y	Y	Y	N	Y	Y	Y	Y	Y	Y
R15X1141	Y	Y	Y	Y	Y	Y	Y	Y	Y	Y	Y	Y	Y
RY713	Y	Y	Y	Y	Y	Y	Y	Y	Y	Y	Y	Y	Y
S37	Y	Y	Y	Y	Y	Y	Y	Y	Y	Y	Y	Y	Y
SC55	Y	Y	Y	Y	Y	Y	Y	Y	Y	Y	Y	Y	Y
SI273	Y	Y	Y	Y	Y	Y	Y	Y	Y	Y	Y	Y	Y
SI446	Y	Y	Y	Y	Y	Y	Y	Y	Y	Y	Y	Y	Y
SY1035	Y	Y	Y	Y	Y	Y	Y	Y	Y	Y	Y	Y	Y
SY1052	Y	Y	Y	Y	Y	Y	Y	Y	Y	Y	Y	Y	Y
SY1128	N	Y	N	N	N	N	N	Y	N	Y	N	N	N
TIAN77	N	N	N	N	N	N	N	N	N	N	N	N	N
TIE7922	Y	Y	Y	Y	Y	Y	Y	Y	Y	Y	Y	Y	Y
TY1	Y	Y	Y	Y	Y	Y	Y	Y	Y	Y	Y	Y	Y
TY11	Y	Y	Y	Y	Y	Y	Y	Y	Y	Y	Y	Y	Y
TY2	Y	Y	Y	Y	Y	Y	Y	Y	Y	Y	Y	Y	Y
TY3	Y	Y	Y	Y	Y	Y	Y	Y	Y	Y	Y	Y	Y
TY4	Y	Y	Y	Y	Y	Y	Y	Y	Y	Y	Y	Y	Y
TY5	Y	Y	Y	Y	Y	Y	Y	Y	Y	Y	Y	Y	Y
TY6	Y	Y	Y	Y	Y	Y	Y	Y	Y	Y	Y	Y	Y
U8112	Y	Y	Y	Y	Y	Y	Y	Y	Y	Y	Y	Y	Y
WH413	Y	Y	Y	Y	Y	Y	Y	Y	Y	Y	Y	Y	Y
WU109	Y	Y	Y	Y	Y	Y	Y	Y	Y	Y	Y	Y	Y
XI502	Y	Y	Y	Y	Y	Y	Y	Y	Y	Y	Y	Y	Y
XUN971	Y	Y	Y	Y	Y	Y	Y	Y	Y	Y	Y	Y	Y
XZ698	Y	Y	Y	Y	Y	Y	Y	Y	Y	Y	Y	Y	Y

YE515	Y	Y	Y	Y	Y	Y	Y	Y	Y	Y	Y	Y	Y
YE52106	N	N	N	N	N	N	N	N	N	N	N	N	N
YE8001	Y	Y	Y	Y	Y	Y	Y	Y	Y	Y	Y	Y	Y
Z2018F	Y	Y	Y	Y	Y	Y	Y	Y	Y	Y	Y	N	Y
ZAC546	N	N	N	N	N	N	N	N	N	N	N	N	N
ZB648	Y	Y	Y	Y	Y	Y	Y	Y	Y	Y	Y	Y	Y
ZH68	Y	Y	Y	Y	Y	Y	Y	Y	Y	Y	Y	Y	Y
ZHENG28	Y	Y	Y	Y	Y	Y	Y	Y	Y	Y	Y	Y	Y
ZHENG29	Y	Y	Y	Y	Y	Y	Y	Y	Y	Y	Y	Y	Y
ZHENG30	Y	Y	Y	Y	Y	Y	Y	Y	Y	Y	Y	Y	Y
ZHENG32	Y	Y	Y	Y	Y	Y	Y	Y	Y	Y	Y	Y	Y
ZHENG35	Y	Y	Y	Y	Y	Y	Y	Y	Y	Y	Y	Y	Y
ZHENG653	Y	Y	Y	Y	Y	Y	Y	Y	Y	Y	Y	Y	Y
ZHI41	Y	Y	Y	Y	Y	Y	Y	Y	Y	Y	Y	Y	Y
ZHONG69	Y	Y	Y	Y	Y	Y	Y	Y	Y	Y	Y	Y	Y
ZONG31	Y	Y	Y	Y	Y	Y	Y	Y	Y	Y	Y	Y	Y
ZZ01	Y	Y	Y	Y	Y	Y	Y	Y	Y	Y	Y	Y	Y
ZZ03	Y	Y	Y	Y	Y	Y	Y	Y	Y	Y	Y	Y	Y

Supplementary Table S3. Premiers and materials used in genomic PCR validation for both ePAV candidates and novel genes.

ePAV/Novel candidates	Primer_Name	5' Primer	3' Primer	Size[a]	Lines in confirmation[b]
GRMZM2G334162	162-3	TGCAGGGAAAACATGGCTCT	GGAATCCAGCCATTGGGTACA	675	maizePAN1_96
GRMZM2G083285	285-4	CACGGGTGGAGTTCAGTGAC	CATTCCAGCTCCTCTACGCA	508	maizePAN1_96
GRMZM2G382273	273-1	GGGGCTGAGATGCAAGGTAA	GGCCCTCCAATTTGTCGGTA	609	maizePAN1_96
AC202110.3-FG004	110.4-4	TTCTGTGGTGCAGGCTGTAA	CAGAGGTGCTGCGATGTAGA	555	maizePAN1_96
AC190526.3_FG004	526.4-2	CAGAGTCTTATCTCCGCTCTCT	CTCAGGTGTGGTGTCTCTGT	691	maizePAN1_96
GRMZM2G038388	388-1	TGCTGAAGGTGAGCTACGAC	TGCAAGTGGAGAAGAGCACA	664	maizePAN2_96
GRMZM5G855005	005-2	GCCGTTCGAGTGCTAGTCT	GCGGAAGGTGTTGACCTGTT	387	maizePAN2_96
GRMZM2G088124	124-3	CGCGGTTCACACCGTAGAAA	CAAACTCACCGCTACCACCT	475	maizePAN2_96
GRMZM5G856011	011-2	TTTACGGTGGTTGTGAGGGG	CGCTGCTGGTCTCTCTACTG	669	maizePAN2_96
AC217293.3_FG007	293.7-7	TCTCGGGTTGTTATAGTACATGGG	GGACAGTGACATCAGACCAACT	609	maizePAN2_96
Unigene_31	31-3	GTGATGGCGAACTGATCTGC	ACTCCTGTCCTCTCAAAGCG	337	maizePAN2_96
Unigene_253	253-2	AGCTCCTGCTGCTTGACATT	TCATTGAAGGGCCTGAGACG	487	maizePAN2_96
Unigene_283	283-6	CACAGTCATACTCCCCTGCC	GACCCAGGAGGTGACATGGA	271	maizePAN2_96
Unigene_361	361-1	CTGCATCTGCCTCTGGTACG	GGAAGCGAACATGGAGACCA	286	maizePAN2_96
Unigene_391	391-1	GGTCCAATACGTTCAAGTGCC	TGCTCGGCGAATGGTATGG	304	maizePAN2_96
Unigene_441	441-1#	TCTAGGTTCTGTGTTGCCTGG	CCGAGGAAACGTTTGGCCT	256	maizePAN2_96
Unigene_503	503-3	TCAAGTCCGAATGGCACCAA	GGCCAATTCTGCATAGCCTC	378	maizePAN2_96
Unigene_741	741-3	AAGGGAATAGGGAGACAGCAC	CTTCCACCTTTCTCCAGGCAT	364	maizePAN2_96
Unigene_1536	1536N-4	TTCAGGAAGAACCGAACGGG	ACAAAGGCCACGAAAGCAGT	351	maizePAN2_96
Unigene_1931	1931-4	AGGTGCGTTAATGGCTACGA	GGCCACCTGTTTAACCTAGC	288	maizePAN2_96
Unigene_441	441-3#	GAGCTGCCAAGCTTCTTTCCT	GCTGGAGCAGGTACTGAATGG	449	
Unigene_441	441-8#	CCTGAACAAGAGGCTGAGGG	CTTGTTTGAAGCAGCCTCGC	320	
Unigene_441	441-10#	GGGCTGATCTAGTCCATTCAG	TAGGGATCCACAAGCCAACAG	254	
Unigene_678	678-1	GTTTGGATGCTTCTCGGCAG	AAAATTGACATCGGGCCTCCT	337	AMP*
Unigene_678	678-5	AAGATCGGTGAAGTTGGTGCT	AACTGAAGCTCTTGGAGACACT	299	
Unigene_678	678-8	GAGAGAAAGCCGACGCACAT	TGGAGAGAATGCGAAGGGTG	365	
Unigene_705	705-1	AGAGGAGATTGCAGAGGGCTA	GACGGGCCTTTGTAGCAAGT	206	

Unigene_705	705-3	CTACCAGCCAAGATGCAAGG	TCAGGAGTCAGGGTGAGAGA	359	
Unigene_705	705-5	ACCCTGACTCCTGATGCCTT	TGAAGTCTGTGCATGGCTCC	368	AMP*
Unigene_705	705-8	GAACTGCACAAACTCCGCATT	TGGATTCATCACCACCTGGG	361	

[#]Primers used to ampligy Unigene_441 full length on genome level.

[*]represents the whole 368 lines used.

[a]Expected Fragment Size.

[b]The detail maizePAN1_96 and maizePAN2_96 of lines used in confirmation are:

maizePAN1_96	1	2	3	4	5	6	7	8	9	10	11	12
A	CIMBL152	CIMBL32	CML162	CIMBL25	CIMBL11	CIMBL89	CIMBL106	CML470	CIMBL28	P178	CML69	CIMBL75
B	CIMBL79	CML169	CIMBL144	CIMBL86	CML479	CIMBL15	CML130	B11	CIMBL121	CIMBL114	CML191	CIMBL54
C	CIMBL147	CIMBL18	CIMBL27	CML496	CIMBL55	CIMBL93	CIMBL105	CML122	CIMBL83	CML32	CML361	CIMBL84
D	647	CIMBL17	CIMBL5	CIMBL119	CIMBL77	CML121	CIMBL4	CIMBL116	CIMBL111	CML493	CIMBL10	CIMBL29
E	CIMBL91	CML433	R15X1141	TY3	GEMS33	L3180	GEMS51	SW92E114	05W002	GEMS5	7327	JH96C
F	GEMS25	GEMS13	18-599	DH3732	81162	GEMS18	GEMS4	GEMS58	ZH68	303WX	526018	GEMS48
G	M153	GEMS19	GEMS39	JH59	EN25	S22	GEMS16	B113	GEMS1	MO113	GEMS9	GEMS35
H	BZN	GEMS62	LK11	150	9642	TY4	CIMBL70	CIMBL157	GEMS23	TY11	MO17	B73

maizePAN2_96	1	2	3	4	5	6	7	8	9	10	11	12
A	CIMBL152	CIMBL32	CML162	CIMBL25	CIMBL11	CIMBL89	CIMBL106	CML470	CIMBL28	P178	CML69	CIMBL75
B	CML431	CML169	CIMBL144	CIMBL86	CML479	CIMBL15	CML130	B11	CIMBL121	CIMBL114	CML191	CIMBL54
C	CIMBL147	CIMBL18	CIMBL27	CML496	CIMBL55	CIMBL93	CIMBL105	CML122	CIMBL83	CML32	CML361	CIMBL84
D	647	CIMBL17	CIMBL5	CIMBL119	CIMBL77	CML121	CIMBL4	CIMBL116	CIMBL111	CML493	CIMBL10	CIMBL29
E	CIMBL91	CML433	R15X1141	TY3	GEMS33	L3180	GEMS51	SW92E114	05W002	GEMS5	7327	JH96C
F	GEMS25	GEMS13	18-599	DH3732	81162	GEMS18	GEMS4	CML480	ZH68	303WX	526018	GEMS48
G	M153	GEMS19	GEMS39	JH59	EN25	S22	GEMS16	B113	GEMS1	MO113	GEMS9	GEMS35
H	BZN	GEMS62	LK11	150	9642	TY4	CIMBL70	CIMBL157	GEMS23	TY11	MO17	B73

Supplementary Table S4. The sequencing and assembly data for 367 inbred lines.

Name of inbred line	# of sequencing reads	# of clear reads	# of de novo assembly transcripts	# of assembled transcripts N50	# of transcripts mapped to genome	# of transcripts unmapped to genome	# of unmapped transcipts N50
Total	27,177,099,764						
150	71,154,356	67,989,403	58,648	1,458	57,683	965	634
177	74,067,886	67,372,669	119,222	776	60,060	59,162	475
238	89,003,114	81,151,891	53,074	1,170	52,069	1,005	568
268	73,957,894	64,244,562	53,040	700	52,019	1,021	435
647	64,760,376	59,929,223	51,415	960	50,448	967	470
1462	63,492,942	60,084,895	54,349	1,337	53,335	1,014	652
4019	80,321,408	70,665,684	48,901	719	47,817	1,084	480
5213	67,129,036	63,880,819	52,317	1,284	51,451	866	630
5237	64,094,426	60,562,023	63,209	1,466	62,001	1,208	638
7327	88,916,144	83,107,018	57,685	838	56,149	1,536	403
7381	80,162,282	73,757,895	57,704	1,029	56,600	1,104	568
8902	71,313,114	63,243,450	46,859	668	46,071	788	432
9642	66,160,368	62,460,607	58,788	1,198	57,802	986	571
81162	71,544,200	68,172,982	53,186	1,353	52,279	907	715
526018	65,217,478	61,444,748	68,665	1,169	67,392	1,273	597
05W002	79,109,902	73,933,974	57,569	1,474	56,528	1,041	708
05WN230	88,806,332	81,720,138	62,838	984	61,683	1,155	560
07KS4	62,934,524	57,656,456	53,218	1,030	52,187	1,031	534
18-599	77,498,858	72,520,033	66,256	1,282	64,939	1,317	640
303WX	77,409,438	70,158,707	54,055	1,106	52,936	1,119	594
4F1	60,066,608	56,704,979	57,068	1,342	55,895	1,173	576
7884-4HT	61,320,948	58,228,710	49,160	1,253	48,215	945	563
835A	85,324,074	79,606,203	69,423	1,196	67,863	1,560	553
835B	59,433,186	56,255,075	51,126	1,115	50,220	906	573
975-12	86,206,228	79,761,352	62,735	1,137	61,540	1,195	557
B11	75,616,014	71,633,981	58,652	1,333	57,781	871	656
B110	56,781,566	53,252,269	52,655	976	51,831	824	553
B111	87,302,688	78,483,320	59,769	975	58,787	982	523
B113	83,703,142	75,439,290	49,595	984	48,652	943	527
B114	72,515,472	67,838,676	54,374	1,414	53,434	940	638
B151	51,442,962	48,416,575	61,363	995	60,131	1,232	512
B73	77,976,204	72,371,474	54,865	1,161	54,201	664	638
B77	94,153,204	88,626,133	67,607	1,242	66,309	1,298	566
BS16	138,757,038	56,967,107	47,007	747	46,027	980	495
BY4839	61,297,094	57,991,221	53,695	1,095	52,705	990	587
BY4944	64,470,292	59,439,611	53,472	1,029	52,488	984	593
BY4960	58,492,050	54,608,160	55,267	903	54,097	1,170	486
BY804	73,413,826	69,100,474	62,220	1,068	60,935	1,285	519
BY807	74,571,402	69,827,174	57,577	860	56,407	1,170	482
BY809	82,799,884	78,107,434	66,085	1,376	64,816	1,269	606
BY813	96,799,706	89,369,644	58,494	1,209	57,384	1,110	548
BY815	78,730,578	73,082,380	56,520	1,189	55,462	1,058	593
BY855	86,605,974	80,646,588	63,051	1,118	61,798	1,253	557

BZN	71,106,736	65,149,379	64,617	983	63,252	1,365	506
CA47	75,623,018	65,580,768	72,346	1,087	70,912	1,434	625
CHANG3	84,594,970	77,190,360	68,699	825	55,032	13,667	445
CHENG698	94,531,988	88,593,628	61,107	1,316	59,996	1,111	648
CHUAN48-2	78,646,998	71,642,805	71,190	1,061	69,909	1,281	567
CI7	68,570,256	64,781,614	52,040	1,070	51,050	990	569
CIMBL1	81,781,976	73,733,660	63,505	1,017	62,324	1,181	500
CIMBL10	83,657,654	77,609,745	59,550	887	58,508	1,042	523
CIMBL100	83,002,884	77,663,407	65,208	838	62,650	2,558	326
CIMBL101	56,269,598	52,183,086	54,401	1,100	53,342	1,059	582
CIMBL102	65,601,926	58,330,216	51,646	1,062	50,723	923	505
CIMBL105	68,790,740	63,788,667	52,637	1,168	51,497	1,140	568
CIMBL106	59,234,902	54,664,337	38,937	700	38,149	788	455
CIMBL108	70,846,506	66,265,496	44,032	872	43,093	939	495
CIMBL109	67,194,254	63,390,159	59,478	1,138	58,081	1,397	520
CIMBL11	70,801,026	65,288,292	56,989	1,264	55,885	1,104	653
CIMBL111	69,306,566	65,700,925	52,728	1,041	51,520	1,208	475
CIMBL113	77,237,934	72,863,542	57,817	957	56,238	1,579	477
CIMBL114	78,505,232	72,597,624	63,504	1,004	62,239	1,265	545
CIMBL115	66,335,744	61,425,996	46,097	839	45,185	912	505
CIMBL116	86,763,078	80,068,392	55,347	1,099	54,289	1,058	639
CIMBL119	74,578,262	69,898,832	55,650	1,028	54,504	1,146	521
CIMBL12	81,424,368	76,277,620	59,078	1,059	57,865	1,213	553
CIMBL120	76,400,272	71,231,297	43,567	749	42,646	921	481
CIMBL121	75,319,522	69,977,611	52,645	1,088	51,696	949	591
CIMBL122	77,304,790	72,701,832	54,002	1,040	52,941	1,061	532
CIMBL123	85,808,086	81,535,253	55,464	979	54,231	1,233	528
CIMBL124	66,488,202	62,429,815	61,106	1,081	59,934	1,172	567
CIMBL125	74,971,470	70,463,099	50,617	1,284	49,724	893	657
CIMBL127	82,539,924	76,835,558	53,735	983	52,622	1,113	537
CIMBL129	85,483,480	80,059,897	65,547	1,177	63,711	1,836	442
CIMBL13	70,116,224	63,881,421	64,733	946	62,590	2,143	370
CIMBL133	72,575,606	67,211,238	54,157	1,025	52,956	1,201	497
CIMBL137	65,645,804	62,135,054	60,994	948	59,806	1,188	520
CIMBL139	70,602,974	66,159,040	57,058	1,214	55,899	1,159	672
CIMBL140	72,812,798	68,850,395	58,930	1,117	57,806	1,124	613
CIMBL141	94,734,388	88,473,000	59,679	1,146	58,517	1,162	597
CIMBL142	81,176,016	76,238,068	57,911	1,316	56,957	954	703
CIMBL143	85,711,218	79,276,627	51,417	939	50,360	1,057	485
CIMBL144	75,799,284	67,596,073	47,060	991	46,278	782	502
CIMBL145	78,562,014	73,689,646	61,575	1,023	58,223	3,352	384
CIMBL147	82,246,854	75,639,070	50,915	612	48,558	2,357	301
CIMBL149	79,155,540	72,608,569	47,441	916	46,441	1,000	555
CIMBL15	78,292,472	72,334,438	59,427	1,101	57,998	1,429	529
CIMBL150	83,989,458	78,890,504	63,509	1,169	62,140	1,369	596
CIMBL151	79,311,998	73,250,698	49,546	905	48,571	975	526
CIMBL152	91,592,274	85,452,246	74,278	1,175	72,081	2,197	433
CIMBL153	72,974,574	66,532,270	73,742	1,033	72,339	1,403	556
CIMBL156	69,935,542	64,763,614	53,119	1,178	52,093	1,026	600

CIMBL157	61,226,278	57,989,312	61,786	1,208	60,484	1,302	572
CIMBL16	71,902,030	67,600,048	58,168	950	57,042	1,126	552
CIMBL17	62,208,856	58,634,601	49,161	1,303	48,292	869	662
CIMBL18	82,588,720	76,203,704	64,062	1,077	62,786	1,276	526
CIMBL19	85,301,328	77,183,725	56,985	907	55,926	1,059	535
CIMBL2	74,489,826	69,122,178	59,540	942	58,444	1,096	527
CIMBL21	84,961,538	78,692,549	62,375	1,289	61,144	1,231	579
CIMBL22	64,463,718	61,219,844	53,554	1,259	52,516	1,038	566
CIMBL23	76,600,428	72,304,113	65,778	1,166	63,362	2,416	369
CIMBL25	72,013,576	67,697,277	55,734	1,227	54,659	1,075	581
CIMBL27	76,767,826	71,435,167	58,761	897	57,583	1,178	508
CIMBL28	67,831,960	63,560,719	52,007	1,140	51,080	927	558
CIMBL29	71,142,850	67,271,408	58,553	1,177	57,256	1,297	532
CIMBL3	92,832,218	85,429,106	61,926	1,221	60,685	1,241	617
CIMBL32	67,018,568	63,350,276	52,789	1,285	51,835	954	649
CIMBL38	77,458,856	71,345,784	60,217	1,191	59,095	1,122	583
CIMBL4	82,143,628	73,406,642	51,775	1,078	50,778	997	519
CIMBL40	87,088,430	80,623,135	57,606	974	56,544	1,062	582
CIMBL42	60,508,242	53,570,782	62,960	1,075	52,558	10,402	418
CIMBL43	62,495,466	58,961,142	65,150	1,200	63,907	1,243	547
CIMBL46	84,044,956	77,511,318	64,229	1,103	62,905	1,324	575
CIMBL47	81,388,644	73,184,427	67,914	1,211	66,586	1,328	653
CIMBL48	76,974,704	71,478,763	62,073	1,138	60,868	1,205	618
CIMBL49	82,121,576	76,234,946	69,392	1,244	68,066	1,326	614
CIMBL5	73,633,666	69,135,755	51,921	1,165	50,829	1,092	575
CIMBL50	77,854,504	66,236,100	53,119	840	51,994	1,125	463
CIMBL51	76,382,534	70,937,011	58,786	1,203	57,752	1,034	606
CIMBL52	71,044,952	66,628,624	57,445	1,038	56,305	1,140	533
CIMBL53	70,320,516	64,633,408	51,882	811	50,711	1,171	497
CIMBL54	70,736,550	63,309,158	62,982	874	61,883	1,099	504
CIMBL55	70,846,022	67,111,762	54,480	1,454	53,440	1,040	623
CIMBL56	63,313,368	59,818,396	52,783	1,013	51,861	922	526
CIMBL58	76,576,534	70,776,385	64,044	1,110	62,810	1,234	613
CIMBL59	64,864,332	57,076,858	47,729	1,058	46,861	868	576
CIMBL6	65,919,562	61,178,256	64,676	1,253	63,384	1,292	587
CIMBL60	80,691,250	74,471,191	51,905	1,246	50,921	984	601
CIMBL62	151,036,694	68,969,704	48,492	807	47,426	1,066	478
CIMBL63	57,398,524	54,419,512	55,184	881	53,873	1,311	467
CIMBL66	62,349,674	59,423,133	55,757	1,377	54,694	1,063	576
CIMBL68	74,750,938	69,258,585	61,624	1,197	60,507	1,117	613
CIMBL69	75,748,016	68,818,355	68,758	1,205	67,471	1,287	581
CIMBL7	67,299,416	62,864,364	56,905	1,241	55,832	1,073	642
CIMBL70	89,705,876	81,699,436	57,239	795	56,035	1,204	507
CIMBL71	84,878,844	78,777,669	59,097	1,038	57,893	1,204	527
CIMBL74	73,968,062	68,224,806	63,079	1,082	61,934	1,145	592
CIMBL75	73,266,508	65,846,482	61,888	1,173	60,699	1,189	629
CIMBL77	81,640,000	74,622,865	61,155	1,272	59,956	1,199	630
CIMBL79	95,023,540	89,004,070	61,062	997	59,570	1,492	485
CIMBL81	89,199,488	82,939,988	58,657	1,073	57,447	1,210	524

CIMBL82	70,645,634	66,526,936	58,116	1,253	57,019	1,097	641
CIMBL83	65,924,326	60,260,858	53,144	1,094	52,178	966	571
CIMBL84	62,702,028	58,509,305	31,351	568	30,628	723	396
CIMBL86	87,388,562	82,325,501	61,040	1,096	59,368	1,672	462
CIMBL87	86,567,242	79,515,790	58,124	973	56,877	1,247	494
CIMBL88	67,194,618	62,079,794	62,023	1,070	60,834	1,189	598
CIMBL89	49,112,550	47,058,925	44,039	1,225	43,275	764	568
CIMBL9	73,933,906	69,550,261	60,197	984	59,155	1,042	541
CIMBL90	79,412,152	74,121,723	53,399	920	52,239	1,160	501
CIMBL91	127,404,302	103,180,422	71,536	1,310	70,014	1,522	675
CIMBL92	89,725,846	81,494,409	62,880	593	54,262	8,618	352
CIMBL93	72,180,048	42,999,835	44,630	861	43,811	819	460
CIMBL94	61,781,248	57,671,715	54,889	1,088	53,921	968	594
CIMBL95	47,078,074	44,946,948	46,954	1,267	46,027	927	684
CIMBL96	58,302,142	55,870,630	55,632	1,002	54,590	1,042	495
CIMBL98	83,916,782	80,077,837	47,815	1,047	46,704	1,111	471
CIMBL99	87,422,846	81,866,608	62,408	1,225	60,992	1,416	514
CML114	153,675,148	67,756,330	49,239	777	48,453	786	477
CML115	66,191,528	62,126,459	57,683	1,361	56,542	1,141	634
CML116	83,926,408	77,772,535	61,127	1,100	59,877	1,250	558
CML118	83,620,078	78,884,504	53,034	1,047	51,965	1,069	543
CML121	70,850,408	66,261,560	52,129	1,167	50,748	1,381	501
CML122	86,206,464	77,914,850	73,595	1,035	72,119	1,476	555
CML130	75,897,528	70,294,096	65,276	1,110	61,305	3,971	402
CML134	71,262,648	63,930,840	58,002	1,082	56,969	1,033	557
CML139	51,636,804	48,528,347	46,816	1,009	45,949	867	602
CML162	82,623,300	76,274,032	71,224	1,290	69,810	1,414	702
CML163	63,403,862	58,875,122	60,322	1,067	58,985	1,337	536
CML165	63,003,298	59,307,384	68,777	1,135	66,812	1,965	416
CML169	79,771,374	74,466,537	52,057	1,006	51,084	973	538
CML170	48,376,850	45,390,975	54,183	933	52,924	1,259	443
CML171	88,103,128	81,450,597	70,588	985	69,162	1,426	517
CML172	45,072,948	41,088,900	53,201	891	52,246	955	533
CML189	78,398,932	73,627,450	62,474	1,017	61,273	1,201	543
CML191	74,162,592	68,206,740	58,345	1,317	57,107	1,238	610
CML192	77,939,220	73,082,273	66,042	1,126	64,712	1,330	597
CML20	69,509,562	65,675,398	51,970	1,204	50,883	1,087	569
CML290	81,105,294	75,116,957	57,328	1,032	56,284	1,044	514
CML298	63,707,130	59,684,068	51,626	1,021	50,573	1,053	579
CML304	85,391,348	78,307,197	69,818	1,294	68,416	1,402	688
CML31	65,553,390	61,869,506	59,602	1,126	58,453	1,149	602
CML32	62,812,034	59,210,625	53,494	1,109	52,418	1,076	519
CML323	74,607,130	69,070,318	61,725	1,385	59,618	2,107	552
CML324	63,705,620	60,261,927	61,052	1,231	60,080	972	578
CML325	81,379,364	74,715,227	60,852	1,283	59,655	1,197	529
CML327	81,344,260	75,914,850	67,116	1,176	65,777	1,339	592
CML338	71,206,518	67,184,734	58,916	1,081	57,754	1,162	573
CML360	90,127,596	82,763,466	60,946	959	59,770	1,176	517
CML361	85,258,100	78,847,829	62,863	863	61,590	1,273	485

CML411	71,024,390	65,934,105	58,916	1,026	57,769	1,147	573
CML415	75,127,228	68,651,833	56,969	1,134	55,957	1,012	617
CML422	80,111,118	73,945,704	56,316	1,304	55,241	1,075	654
CML423	52,419,802	49,674,378	52,189	1,085	51,050	1,139	533
CML426	66,324,782	62,890,005	54,420	1,328	53,427	993	595
CML431	68,997,148	64,567,339	61,858	1,244	60,656	1,202	678
CML432	70,643,408	66,124,309	52,742	1,203	51,710	1,032	638
CML433	74,139,036	67,412,436	62,376	954	61,163	1,213	511
CML454	63,445,532	60,248,452	61,729	1,273	60,404	1,325	603
CML470	62,788,600	58,983,940	55,063	1,062	54,127	936	534
CML479	86,261,610	79,658,545	54,368	972	53,192	1,176	526
CML480	94,754,972	88,130,176	54,909	968	53,521	1,388	483
CML486	83,072,180	76,991,368	59,995	1,048	58,832	1,163	588
CML493	80,956,390	74,599,522	66,295	1,233	64,992	1,303	603
CML496	68,086,602	64,302,643	63,074	1,058	61,451	1,623	476
CML50	82,941,384	78,135,395	NA	NA	NA	NA	NA
CML69	71,330,730	65,823,269	63,882	1,020	62,669	1,213	578
D863F	90,576,918	81,947,209	69,570	1,175	68,143	1,427	582
DAN3130	69,539,608	66,076,875	55,255	1,402	54,282	973	622
DAN340	60,176,460	57,221,045	51,809	1,390	50,849	960	800
DAN360	71,355,216	66,637,841	56,455	1,072	55,468	987	540
DAN4245	85,023,006	78,847,144	60,022	966	58,756	1,266	529
DAN599	60,442,712	56,391,544	56,390	1,066	55,312	1,078	577
DH3732	75,104,830	70,175,087	59,040	1,215	57,779	1,261	596
DONG237	75,720,692	69,707,693	59,272	1,185	57,875	1,397	587
DONG46	54,776,498	52,042,860	50,473	1,275	49,406	1,067	665
EN25	70,587,634	64,491,258	55,853	1,048	54,790	1,063	524
ES40	78,987,648	73,032,223	51,505	803	50,406	1,099	517
FCD0602	53,232,510	49,924,201	53,773	866	52,877	896	503
GEMS1	61,442,690	57,910,795	48,505	1,301	47,660	845	640
GEMS10	87,237,886	80,394,774	68,361	1,116	67,335	1,026	611
GEMS11	95,039,280	86,314,082	63,482	875	62,367	1,115	543
GEMS13	48,251,550	45,947,136	56,331	1,039	55,408	923	609
GEMS14	66,544,040	63,242,199	60,473	1,128	59,184	1,289	547
GEMS15	85,053,166	76,248,303	61,427	1,082	60,427	1,000	616
GEMS16	70,721,864	64,713,334	56,399	1,153	55,460	939	564
GEMS17	82,016,332	76,485,893	61,516	1,073	60,554	962	590
GEMS18	71,711,852	63,747,825	52,856	996	52,039	817	553
GEMS19	73,349,664	66,928,277	52,434	927	51,434	1,000	539
GEMS2	83,849,608	74,854,358	53,254	1,219	52,364	890	602
GEMS20	72,232,554	66,448,072	44,870	857	44,099	771	575
GEMS21	68,724,702	62,617,389	54,767	927	53,957	810	523
GEMS23	75,738,180	70,343,900	76,773	932	75,366	1,407	530
GEMS25	53,338,318	51,237,903	50,774	1,321	49,885	889	591
GEMS28	99,254,032	88,773,613	67,504	924	66,185	1,319	483
GEMS29	60,538,610	53,354,473	56,234	1,266	55,339	895	600
GEMS3	61,879,808	57,563,531	51,422	1,062	50,515	907	533
GEMS30	85,424,012	79,313,137	62,355	1,070	61,207	1,148	646
GEMS31	79,752,266	71,590,792	79,856	1,206	78,519	1,337	577

GEMS32	57,230,614	53,855,948	54,901	893	53,796	1,105	542
GEMS33	68,982,250	65,123,644	57,263	1,087	56,193	1,070	604
GEMS35	61,219,932	57,165,655	56,867	1,052	55,822	1,045	583
GEMS36	56,424,328	50,235,738	60,450	1,219	59,380	1,070	593
GEMS37	63,096,962	59,584,637	62,976	1,198	61,712	1,264	601
GEMS39	85,587,498	77,197,934	64,745	1,160	63,504	1,241	555
GEMS4	60,387,378	55,424,835	52,989	904	52,119	870	561
GEMS40	82,131,804	75,666,913	47,778	853	46,720	1,058	467
GEMS41	81,553,616	69,777,990	58,525	1,201	57,395	1,130	645
GEMS42	70,728,418	66,382,656	56,625	1,109	55,642	983	569
GEMS44	64,274,782	60,584,618	53,287	1,401	52,358	929	601
GEMS46	80,785,764	76,014,799	62,414	1,496	61,177	1,237	701
GEMS48	46,923,244	44,990,024	48,497	1,308	47,442	1,055	618
GEMS49	71,311,580	64,819,401	62,862	1,224	61,884	978	645
GEMS5	90,759,726	84,035,864	58,312	1,009	57,311	1,001	574
GEMS50	74,567,740	68,103,764	68,141	1,099	67,134	1,007	620
GEMS51	57,426,794	53,148,131	56,501	949	55,671	830	646
GEMS54	77,541,908	73,635,744	57,811	1,102	56,839	972	565
GEMS55	63,775,062	56,931,343	59,839	890	58,780	1,059	568
GEMS56	73,508,056	65,138,896	46,421	791	45,443	978	519
GEMS58	72,076,596	67,152,952	59,084	916	57,975	1,109	505
GEMS59	60,215,176	53,040,998	58,263	987	57,214	1,049	577
GEMS6	76,427,110	68,535,046	49,025	988	48,252	773	580
GEMS60	70,316,150	65,160,238	52,540	998	51,608	932	539
GEMS61	79,599,176	71,787,452	50,816	1,193	50,071	745	601
GEMS62	49,022,492	47,276,661	41,840	1,091	41,231	609	637
GEMS63	62,356,760	57,782,311	54,090	896	53,295	795	583
GEMS64	82,014,086	74,148,880	55,595	916	54,774	821	559
GEMS65	80,901,830	74,163,013	65,877	926	64,874	1,003	519
GEMS66	71,414,776	68,028,558	57,594	1,437	56,730	864	656
GEMS9	85,114,194	78,764,819	61,148	1,228	60,169	979	623
GY1007	69,757,540	65,543,748	58,630	1,457	57,548	1,082	640
GY1032	66,160,726	61,483,513	53,370	1,066	52,418	952	565
GY386	70,840,166	65,342,405	49,829	1,109	48,904	925	565
GY462	71,203,036	66,939,019	59,866	1,036	58,506	1,360	515
GY798	85,735,534	76,792,320	54,288	1,284	53,246	1,042	695
GY923	75,931,462	70,966,001	59,820	1,206	58,091	1,729	528
HTH-17	61,376,256	57,908,475	49,203	1,346	48,174	1,029	597
HUA83-2	76,659,774	71,786,139	55,520	1,205	54,506	1,014	580
HYS	70,587,046	65,634,910	60,003	900	58,901	1,102	525
HZS	65,724,444	61,892,708	54,171	1,366	52,995	1,176	578
IRF314	83,093,092	77,078,569	59,138	968	57,992	1,146	548
J4112	59,846,744	56,720,505	56,263	824	54,711	1,552	417
JH59	75,830,982	68,826,965	50,089	780	49,024	1,065	420
JH96C	57,247,842	54,131,265	55,224	976	54,268	956	550
JI63	93,716,334	85,673,167	54,230	1,183	53,268	962	564
JI842	63,449,058	60,030,497	57,718	913	56,628	1,090	506
JI846	52,970,996	50,530,371	47,557	1,274	46,718	839	582
JI853	75,463,542	69,164,101	63,958	1,130	62,785	1,173	614

JIAO51	71,076,212	66,848,724	54,404	976	53,203	1,201	531
JY01	75,685,284	66,647,375	48,922	889	47,959	963	514
K10	67,016,706	61,071,272	50,251	1,021	49,333	918	544
K12	59,399,130	54,740,941	50,533	1,107	49,508	1,025	577
K14	82,660,326	75,988,338	69,399	1,192	68,139	1,260	603
K22	71,064,624	65,539,147	60,499	732	57,965	2,534	314
L3180	79,891,070	72,182,536	52,007	1,066	51,125	882	603
LG001	69,917,304	63,741,587	60,928	1,214	59,855	1,073	543
LIAO138	50,209,982	47,395,249	49,372	1,320	48,576	796	618
LIAO159	64,083,682	55,562,502	54,609	1,142	53,690	919	647
LIAO5114	89,587,918	82,744,699	60,846	1,303	59,777	1,069	612
LIAO5262	70,767,734	66,119,909	70,262	1,024	68,911	1,351	581
LIAO5263	71,943,110	64,434,849	52,422	603	51,429	993	411
LK11	57,309,170	54,213,711	50,014	878	49,155	859	483
LV28	69,783,502	63,030,267	45,328	1,005	44,514	814	569
LX9801	71,153,244	63,822,535	59,478	978	57,953	1,525	459
LXN	65,128,044	61,721,184	49,574	947	48,542	1,032	512
LY042	84,811,004	77,588,248	61,586	1,293	60,487	1,099	778
M153	83,135,918	75,482,623	64,069	864	62,896	1,173	493
M97	63,495,564	57,299,121	52,871	893	51,959	912	502
MO113	68,202,840	64,153,098	51,870	1,312	50,930	940	623
MO17	81,825,344	75,127,410	58,531	1,331	57,426	1,105	648
NAN21-3	67,952,414	61,867,709	58,993	854	58,028	965	524
P178	75,152,032	70,754,234	58,227	674	56,792	1,435	441
Q1261	78,847,176	73,296,447	56,514	1,058	55,548	966	591
QI205	67,734,344	63,725,082	74,261	1,142	72,737	1,524	590
R15	73,781,518	69,219,740	58,728	848	56,567	2,161	342
R15X1141	76,411,356	65,238,511	54,646	772	53,703	943	481
RY713	75,519,112	68,945,828	54,677	1,095	53,607	1,070	607
RY729	72,664,318	68,459,834	60,707	1,242	59,598	1,109	641
S22	85,812,736	78,149,132	56,363	989	55,243	1,120	507
S37	97,936,806	90,875,119	65,564	1,038	64,221	1,343	546
SC55	66,259,118	62,420,218	56,709	1,152	55,495	1,214	537
SHEN5003	64,191,390	60,474,237	50,893	1,023	49,640	1,253	559
SI273	76,725,670	68,761,181	58,093	790	57,082	1,011	491
SI434	63,879,968	57,365,999	52,768	886	51,057	1,711	418
SI446	52,957,188	49,692,646	44,929	1,218	43,913	1,016	572
SW92E114	74,899,754	69,180,301	57,936	1,126	56,847	1,089	592
SY1032	80,958,672	76,148,372	55,447	1,269	54,083	1,364	546
SY1035	60,104,616	57,319,555	55,770	1,113	54,742	1,028	577
SY1039	63,603,936	58,810,505	43,568	716	42,599	969	473
SY1052	67,688,420	60,297,851	56,915	948	55,999	916	489
SY1128	69,089,498	63,310,315	49,867	1,137	48,996	871	600
SY3073	80,842,392	73,754,285	56,505	1,098	55,482	1,023	527
TIAN77	78,509,692	72,596,439	75,317	1,009	73,688	1,629	498
TIE7922	70,271,860	63,371,338	64,878	1,003	63,823	1,055	582
TY1	81,178,174	73,688,039	63,787	1,240	62,574	1,213	686
TY11	77,982,234	71,082,874	65,643	976	64,450	1,193	542
TY2	81,719,152	76,693,220	56,518	1,155	55,374	1,144	554

TY3	76,708,566	69,051,745	64,582	788	63,375	1,207	510
TY4	70,056,352	63,605,037	49,609	967	48,679	930	538
TY5	54,496,776	50,562,085	56,149	846	55,094	1,055	527
TY6	72,044,962	65,019,210	64,604	1,113	63,456	1,148	618
U8112	79,813,672	74,603,240	58,640	1,189	57,748	892	609
W138	61,404,974	57,561,343	48,900	1,293	47,954	946	616
WH413	85,534,676	78,532,179	72,608	1,196	71,103	1,505	604
WU109	81,166,232	73,441,981	65,877	930	64,021	1,856	424
XI502	54,411,522	52,263,779	51,761	1,315	50,781	980	603
XUN971	65,005,566	61,088,183	49,399	1,037	48,593	806	606
XZ698	61,758,894	24,484,828	39,499	1,025	38,774	725	577
YE478	64,910,354	60,056,438	55,665	1,088	54,685	980	606
YE515	80,071,908	73,152,411	76,834	1,072	75,270	1,564	615
YE52106	72,490,376	65,204,072	54,781	1,017	53,685	1,096	595
YE8001	86,524,576	74,476,476	53,209	939	52,278	931	485
YU374	75,066,746	70,665,653	54,420	1,208	53,418	1,002	608
Z2018F	82,027,876	76,807,236	66,866	1,092	65,565	1,301	618
ZAC546	70,445,950	62,358,930	58,373	793	57,191	1,182	472
ZB648	73,757,236	69,132,986	66,611	1,466	64,430	2,181	527
ZH68	61,431,892	58,371,743	56,956	1,397	56,028	928	737
ZHENG28	61,114,722	57,684,315	57,279	1,253	56,243	1,036	581
ZHENG29	75,555,780	70,239,216	69,283	1,286	68,133	1,150	668
ZHENG30	68,736,856	62,018,101	66,419	1,219	65,233	1,186	593
ZHENG32	60,177,092	52,771,915	52,432	1,058	51,736	696	608
ZHENG35	57,466,676	55,142,730	52,331	1,337	51,485	846	625
ZHENG653	83,425,248	77,753,920	63,305	1,041	62,077	1,228	552
ZHI41	49,339,866	45,787,953	49,850	840	48,959	891	485
ZHONG69	72,684,680	66,521,985	58,256	959	57,035	1,221	531
ZONG31	77,523,890	67,358,223	52,657	1,030	51,687	970	624
ZZ01	69,822,442	64,355,580	58,154	1,007	57,057	1,097	494
ZZ03	64,645,334	57,044,478	32,005	544	31,227	778	379

Supplementary Table S5. GO enrichment of novel genes compared to reference genome.

GO term	Ontology	Description	Number in input list	Number in BG/Ref	p-value	FDR
GO:0032502	P	developmental process	32	143	2.70E-26	9.80E-24
GO:0007275	P	multicellular organismal development	31	138	1.40E-25	2.50E-23
GO:0009607	P	response to biotic stimulus	19	50	3.40E-21	4.20E-19
GO:0009653	P	anatomical structure morphogenesis	12	14	4.10E-20	3.80E-18
GO:0000003	P	reproduction	26	207	3.10E-15	2.30E-13
GO:0009056	P	catabolic process	58	1063	3.60E-14	2.20E-12
GO:0007049	P	cell cycle	15	70	6.70E-13	3.50E-11
GO:0040007	P	growth	9	16	1.00E-12	4.70E-11
GO:0048856	P	anatomical structure development	12	54	8.30E-11	3.40E-09
GO:0016049	P	cell growth	7	12	2.60E-10	8.70E-09
GO:0008361	P	regulation of cell size	7	12	2.60E-10	8.70E-09
GO:0030154	P	cell differentiation	7	14	1.10E-09	3.10E-08
GO:0006259	P	DNA metabolic process	43	871	1.00E-09	3.10E-08
GO:0009719	P	response to endogenous stimulus	13	85	1.90E-09	4.90E-08
GO:0048869	P	cellular developmental process	7	15	2.00E-09	4.90E-08
GO:0009790	P	embryonic development	5	8	6.80E-08	1.60E-06
GO:0090066	P	regulation of anatomical structure size	7	34	1.30E-06	2.60E-05
GO:0032535	P	regulation of cellular component size	7	34	1.30E-06	2.60E-05
GO:0015074	P	DNA integration	7	56	4.00E-05	0.00077
GO:0016043	P	cellular component organization	42	1332	0.00012	0.0021
GO:0006996	P	organelle organization	26	683	0.00013	0.0022
GO:0006139	P	nucleobase, nucleoside, nucleotide and nucleic acid metabolic process	135	6114	0.0016	0.025
GO:0019748	P	secondary metabolic process	7	101	0.0015	0.025
GO:0006278	P	RNA-dependent DNA replication	6	77	0.0018	0.028
GO:0007010	P	cytoskeleton organization	8	145	0.0031	0.046
GO:0003682	F	chromatin binding	13	63	3.80E-11	6.20E-09
GO:0009536	C	plastid	30	99	4.50E-29	6.60E-27
GO:0005886	C	plasma membrane	30	179	6.30E-21	4.60E-19
GO:0005773	C	vacuole	14	52	1.30E-13	6.20E-12
GO:0044444	C	cytoplasmic part	96	2774	2.90E-10	1.00E-08
GO:0043231	C	intracellular membrane-bounded organelle	121	4214	7.60E-08	2.20E-06
GO:0043227	C	membrane-bounded organelle	121	4248	1.10E-07	2.70E-06
GO:0005737	C	cytoplasm	108	3701	1.50E-07	3.10E-06
GO:0005829	C	cytosol	15	175	3.20E-07	5.90E-06
GO:0005739	C	mitochondrion	21	375	2.20E-06	3.50E-05
GO:0031981	C	nuclear lumen	9	159	0.0015	0.021
GO:0043226	C	organelle	138	6362	0.0025	0.033
GO:0043229	C	intracellular organelle	136	6360	0.0041	0.05

Supplementary Table S6. Novel gene LD mapping. The predicted location of novel genes based on LD mapping to the reference genome.

Novel genes	LDmax_SNP*	chr	position	r^2
Unigene_1026	PZE-103026200	1	518354	1
Unigene_1093	PZE-104051018	1	4768251	1
Unigene_1119	PZE-102121540	1	7302509	1
Unigene_272	chr4.S_67945227	1	9435531	1
Unigene_300	PZE-110043178	1	10089472	1
Unigene_304	PZE-101130019	1	10998864	1
Unigene_333	PZE-109036626	1	14625059	1
Unigene_541	chr3.S_191368421	1	19262211	1
Unigene_611	chr5.S_190605873	1	32869478	1
Unigene_743	SYN2764	1	35848782	1
Unigene_819	chr3.S_183262547	1	36221209	1
Unigene_824	chr7.S_13616012	1	47810543	1
Unigene_832	PZE-102135971	1	53422302	1
Unigene_857	PZE-109054108	1	72842586	1
Unigene_920	PZE-102129186	1	76881782	1
Unigene_930	PZE-102129186	1	77149519	1
Unigene_939	PZE-102129186	1	77149519	1
Unigene_159	PZE-108028368	1	86166647	0.9862964
Unigene_385	chr8.S_72932205	1	86166647	0.9726101
Unigene_352	chr3.S_225587565	1	92431586	0.9626835
Unigene_294	chr9.S_151999544	1	98297572	0.9596002
Unigene_219	chr3.S_191997692	1	119214747	0.954023
Unigene_270	PZE-108052790	1	119378679	0.9433706
Unigene_894	PZE-102126983	1	143532953	0.9342544
Unigene_881	PZE-103051373	1	145099792	0.9084467
Unigene_524	SYN10709	1	148453754	0.8782654
Unigene_519	PZE-110033485	1	148457968	0.8571429
Unigene_577	PZE-102121540	1	157135183	0.8541667
Unigene_542	chr4.S_28921826	1	166296457	0.8386111
Unigene_317	chr4.S_32810595	1	169463687	0.8320346
Unigene_387	PZE-104085342	1	174174983	0.8311133
Unigene_641	PZE-106010721	1	176758066	0.7893557
Unigene_654	PZE-101188882	1	177032959	0.7847619
Unigene_780	SYN15878	1	183776243	0.7817631
Unigene_502	PZE-102129186	1	184246349	0.7777778
Unigene_570	chr8.S_170434394	1	188459255	0.7723708
Unigene_656	PZE-109029438	1	193524090	0.7720723
Unigene_373	PZE-106094290	1	194663220	0.7619048
Unigene_141	PZE-110069821	1	213153061	0.7575758
Unigene_601	PZE-103051373	1	215444010	0.755102
Unigene_513	PZE-102175131	1	219064219	0.7187882
Unigene_471	PZE-101117838	1	220375892	0.7142857
Unigene_625	PZE-109061894	1	233584720	0.7071429
Unigene_1064	PZE-108032382	1	234274891	0.7002384
Unigene_740	PZE-103072192	1	234274891	0.6944444
Unigene_662	SYN31236	1	234274891	0.6932407

Unigene_624	PZE-103001421	1	234274891	0.6923999
Unigene_660	PZE-102121540	1	234274891	0.6861111
Unigene_918	SYN6397	1	234274891	0.6857143
Unigene_696	SYN33290	1	234274891	0.6759589
Unigene_108	PZE-104051018	1	234274891	0.6746032
Unigene_563	PZE-104018476	1	234274891	0.6733695
Unigene_612	chr1.S_219064219	1	248461517	0.6700577
Unigene_1369	PUT-163a-93281192-4841	1	252257234	0.6666667
Unigene_424	chr5.S_132171182	1	255253531	0.6612245
Unigene_21	PZE-106021425	1	258602379	0.6553646
Unigene_109	chr1.S_296165974	1	269288137	0.6364853
Unigene_736	PZE-101188882	1	269299215	0.630102
Unigene_336	chr5.S_61235211	1	272937172	0.6238311
Unigene_1002	chr6.S_87408682	1	280581424	0.6209205
Unigene_522	PZE-104018476	1	280706296	0.6205357
Unigene_347	chr4.S_11466905	1	280706296	0.6153846
Unigene_275	PZE-101244646	1	280706296	0.6143809
Unigene_776	PZE-101188882	1	288394210	0.6136364
Unigene_777	PZE-104017281	1	288394832	0.6136364
Unigene_722	PZE-105065508	1	290687056	0.612985
Unigene_291	chr8.S_151186202	1	290857647	0.6112139
Unigene_377	chr7.S_134972234	1	292524761	0.6090753
Unigene_223	PZE-105109096	1	294634137	0.6067323
Unigene_399	chr2.S_210529765	1	295536570	0.6060388
Unigene_621	chr2.S_10664464	1	296165974	0.6045455
Unigene_738	PZE-103102545	1	296985852	0.6005952
Unigene_1127	PZE-103069120	2	273615	0.6
Unigene_1293	PZE-103001421	2	3389929	0.6
Unigene_1049	PZE-110055234	2	9014897	0.595
Unigene_145	chr3.S_8271283	2	10664464	0.5945064
Unigene_1101	PZE-104063838	2	12102605	0.5853193
Unigene_218	PZE-102121540	2	19265678	0.5833681
Unigene_628	chr2.S_186790092	2	22614457	0.5819738
Unigene_547	PZE-103051373	2	22951381	0.5734286
Unigene_62	PZE-105051636	2	26883750	0.566675
Unigene_130	PZE-102092530	2	28666434	0.5661376
Unigene_249	PZE-103069120	2	28666441	0.5634921
Unigene_1210	PZE-102092530	2	31151790	0.5625
Unigene_340	SYN11547	2	36835546	0.5625
Unigene_209	chr8.S_6661546	2	39762577	0.562023
Unigene_980	PZE-103036506	2	41395901	0.5601765
Unigene_461	chr8.S_78832554	2	48823221	0.5575319
Unigene_384	PZE-105163307	2	57108018	0.5573557
Unigene_692	PZE-103026200	2	61470572	0.5565634
Unigene_724	SYN4382	2	69944926	0.5565634
Unigene_549	PZE-102092530	2	99381840	0.5555556
Unigene_721	PZE-101188882	2	102755663	0.5555556
Unigene_971	SYN7625	2	102755663	0.5555556

Unigene_714	PZE-101051879	2	102755663	0.5544751
Unigene_587	PZE-103102545	2	102755663	0.5511938
Unigene_363	SYN37343	2	102755663	0.5493081
Unigene_878	PZE-102048632	2	102755663	0.5478458
Unigene_491	chr3.S_186747886	2	102755663	0.547689
Unigene_364	PZE-102063029	2	102755663	0.5476437
Unigene_821	SYN23045	2	102755663	0.5443097
Unigene_817	PZE-103072192	2	102755663	0.5428338
Unigene_565	PZE-102092530	2	102755663	0.5420754
Unigene_1025	PZE-101149898	2	102755663	0.5412698
Unigene_786	PZE-103001421	2	102755663	0.5396907
Unigene_536	chr5.S_74919020	2	102755663	0.539556
Unigene_949	PZE-103066554	2	102755663	0.5384615
Unigene_535	PZE-102092530	2	102755663	0.5361111
Unigene_496	PZE-109036028	2	102755663	0.5333333
Unigene_566	PZE-102187794	2	102755663	0.5301058
Unigene_837	PZE-102129186	2	102755663	0.5275161
Unigene_593	PZE-107074954	2	102755663	0.5267806
Unigene_914	SYN6353	2	102755663	0.5238095
Unigene_978	PZE-108030912	2	102755663	0.5228402
Unigene_652	PZE-102129186	2	102755663	0.52
Unigene_884	SYN2399	2	102755663	0.52
Unigene_1161	chr1.S_53422302	2	102755663	0.5173856
Unigene_239	PZE-106020174	2	121239237	0.5156396
Unigene_645	PZE-102092530	2	127444944	0.5145377
Unigene_580	PZE-103091301	2	130401148	0.5132308
Unigene_212	chr5.S_216738630	2	144680258	0.5097002
Unigene_648	PZE-101171899	2	144680258	0.5075466
Unigene_794	SYN6397	2	144680258	0.5070034
Unigene_709	PZE-104045519	2	145778654	0.506944
Unigene_647	chr3.S_168482460	2	146787068	0.5037016
Unigene_316	PZE-110073459	2	149335106	0.5029001
Unigene_765	SYN16332	2	152210101	0.5022222
Unigene_488	PZE-107031895	2	156339094	0.5
Unigene_862	PZE-102092530	2	158610701	0.5
Unigene_68	SYN6229	2	164191793	0.4995268
Unigene_250	chr9.S_8417637	2	167327320	0.4966168
Unigene_229	PZE-102076145	2	167327320	0.4938432
Unigene_248	chr2.S_22614457	2	167327320	0.4935131
Unigene_390	SYN17067	2	167327320	0.4928767
Unigene_615	SYN10319	2	167327320	0.4906122
Unigene_1061	PZE-105000145	2	167327320	0.4903061
Unigene_762	chr8.S_17303969	2	167327320	0.4897595
Unigene_315	PZE-101188882	2	167327320	0.4890467
Unigene_256	PZE-101143143	2	167327320	0.4888383
Unigene_793	PZE-102092530	2	167327320	0.4853395
Unigene_1434	PZE-108091013	2	167327320	0.4848485
Unigene_178	chr2.S_61470572	2	176638484	0.4819849
Unigene_153	PZE-103054685	2	177183870	0.4796975

Unigene_875	PZE-110042839	2	179117618	0.4778265
Unigene_1107	PZE-103026200	2	179117618	0.4761905
Unigene_449	PZE-102111568	2	179117618	0.4761905
Unigene_417	PZE-103055059	2	179117618	0.4760906
Unigene_405	chr10.S_124299289	2	179117618	0.4724085
Unigene_808	SYN24264	2	179117618	0.4718261
Unigene_29	PZE-108069434	2	179117618	0.4701747
Unigene_383	PZE-101094490	2	179117618	0.4700008
Unigene_428	SYN24468	2	179117618	0.467096
Unigene_726	chr7.S_171700452	2	179117618	0.4652521
Unigene_649	PZE-107070842	2	179117618	0.4642857
Unigene_711	PZE-103051373	2	179117618	0.464
Unigene_739	PUT-163a-89249846-4643	2	179117618	0.4632022
Unigene_810	chr4.S_239409883	2	179117618	0.458579
Unigene_847	PZE-102101002	2	179117618	0.4582973
Unigene_718	SYN12604	2	183930527	0.4580766
Unigene_265	chr4.S_84084057	2	185584843	0.456587
Unigene_560	SYN11547	2	185584843	0.4539505
Unigene_745	PZE-102061433	2	185584843	0.4515625
Unigene_733	PZE-101086807	2	185584843	0.45
Unigene_974	PZE-106017241	2	186790092	0.45
Unigene_619	SYN13897	2	188152622	0.4470286
Unigene_180	chr8.S_158895597	2	192575599	0.4460297
Unigene_354	chr3.S_189466771	2	193734967	0.4421299
Unigene_366	PZE-100001219	2	200090164	0.4418085
Unigene_797	PZE-103051373	2	201050906	0.4406857
Unigene_478	PZE-107023698	2	205949375	0.4398118
Unigene_202	chr10.S_18674222	2	209508559	0.4392565
Unigene_809	PZE-104069761	2	210529765	0.438961
Unigene_634	chr4.S_143083152	2	212884412	0.4327275
Unigene_359	PZE-108015015	2	217637431	0.4317641
Unigene_1075	PZE-107053869	2	221130024	0.4285714
Unigene_904	PZE-103055189	2	226679380	0.4285714
Unigene_492	chr3.S_175705643	2	226679380	0.4279263
Unigene_296	chr9.S_96775630	2	226679380	0.4255339
Unigene_1023	chr4.S_154261101	2	231908887	0.4239151
Unigene_422	chr8.S_140749569	2	231944265	0.4233666
Unigene_844	PZE-108030912	3	1554135	0.4225
Unigene_799	chr1.S_295536570	3	1554135	0.4223781
Unigene_572	SYN4994	3	1614290	0.4218501
Unigene_553	chr9.S_131375711	3	1614290	0.4211026
Unigene_520	PZE-102153463	3	1614290	0.4202391
Unigene_596	PZE-103051373	3	1614290	0.4201389
Unigene_444	PZE-105095752	3	2291828	0.4194083
Unigene_742	PZE-103142007	3	5336296	0.4193891
Unigene_1012	SYN30519	3	8271283	0.417091
Unigene_427	PZE-102092530	3	12414263	0.4166667
Unigene_578	PZE-102092530	3	13464342	0.4166667

Unigene_916	PZE-107067415	3	15040324	0.416324
Unigene_297	PZE-103051373	3	19246348	0.4161429
Unigene_314	PZE-108064817	3	19250132	0.4142809
Unigene_870	PZE-103026200	3	19250132	0.4137428
Unigene_744	PZE-108083973	3	19250132	0.4134279
Unigene_556	PZE-109067808	3	19250132	0.4129724
Unigene_455	chr3.S_41184350	3	19250132	0.4127681
Unigene_157	PZE-102121540	3	19250132	0.4118241
Unigene_351	SYN24468	3	19250132	0.4105038
Unigene_350	SYN28693	3	19250132	0.4101017
Unigene_451	PZE-102184087	3	19250132	0.4093716
Unigene_533	SYN7850	3	20190023	0.4087983
Unigene_323	SYN38969	3	22376124	0.4080722
Unigene_483	PZE-103187403	3	25136211	0.4064695
Unigene_433	PZE-102121540	3	30040876	0.405754
Unigene_661	PZE-103102545	3	41184350	0.4048821
Unigene_1024	PZE-106009031	3	45037281	0.4043057
Unigene_369	SYN9021	3	45037281	0.40365
Unigene_785	PZE-104051267	3	56901363	0.4013658
Unigene_924	PZE-103051373	3	56901363	0.4
Unigene_1174	chr2.S_209508559	3	56901363	0.3993182
Unigene_1123	SYN22622	3	56901363	0.3991422
Unigene_1045	PZE-106041967	3	56901363	0.3988817
Unigene_267	PZE-103066587	3	56901363	0.3974039
Unigene_418	chr10.S_4991331	3	56901363	0.3973967
Unigene_242	SYN15586	3	56901363	0.3965167
Unigene_683	chr5.S_163412915	3	56901363	0.3951727
Unigene_876	chr1.S_157135183	3	56901363	0.3912698
Unigene_710	PZE-103051373	3	56901363	0.390625
Unigene_685	PZE-102103390	3	56901363	0.3902185
Unigene_124	PZE-108091013	3	56901363	0.3895875
Unigene_675	SYN320	3	56901363	0.3885159
Unigene_540	SYN15586	3	56901363	0.388141
Unigene_327	PZE-105065508	3	56901363	0.3875
Unigene_843	PZE-108015441	3	56901363	0.3869103
Unigene_1126	chr5.S_8331089	3	56901363	0.3867277
Unigene_420	chr1.S_7302509	3	56901363	0.386608
Unigene_763	PZE-102104828	3	56901363	0.3862765
Unigene_298	chr1.S_76881782	3	56901363	0.3856977
Unigene_504	PZE-102092530	3	56901363	0.3845238
Unigene_343	PZE-102092530	3	56901363	0.3839229
Unigene_356	chr1.S_294634137	3	56901363	0.3838219
Unigene_694	PZE-104028451	3	56901363	0.3834586
Unigene_604	PZE-102135971	3	56901363	0.3829042
Unigene_698	PZE-104065771	3	56901363	0.3824094
Unigene_962	PZE-106041967	3	56901363	0.3820106
Unigene_63	chr9.S_120803982	3	56901363	0.3810996
Unigene_517	chr7.S_172472359	3	56901363	0.380738
Unigene_372	SYN15163	3	56901363	0.3788312

Unigene_499	PZE-102092530	3	58297700	0.3777778
Unigene_116	PZE-103032411	3	64579310	0.3724825
Unigene_630	chr1.S_269288137	3	65660811	0.3722909
Unigene_938	chr2.S_164191793	3	65662918	0.3710651
Unigene_1008	SYN19282	3	65926400	0.3703704
Unigene_481	PZE-107046163	3	66863296	0.3702305
Unigene_357	chr8.S_159674303	3	86041823	0.3689997
Unigene_97	PZE-107020509	3	86041823	0.3672508
Unigene_631	PZE-107047532	3	86041823	0.3669811
Unigene_143	SYN15878	3	86041823	0.3650391
Unigene_295	chr5.S_60657192	3	86041823	0.3648524
Unigene_361	PZE-103069120	3	86041823	0.3636364
Unigene_602	PZE-101206240	3	86041823	0.3624709
Unigene_74	chr1.S_183776243	3	86041823	0.3618132
Unigene_301	PZE-103102545	3	86041823	0.3616756
Unigene_607	PZE-105134617	3	86041823	0.3580674
Unigene_787	SYN13776	3	86041823	0.357773
Unigene_545	PZE-103051373	3	86041823	0.3575827
Unigene_368	PZE-101188882	3	86041823	0.3565605
Unigene_885	PZE-104018476	3	91005175	0.3565525
Unigene_430	PZE-102129186	3	91201940	0.3559524
Unigene_432	PZE-102129186	3	91910150	0.3550183
Unigene_36	chr8.S_6396539	3	99728156	0.3522907
Unigene_72	PZE-103087010	3	118961656	0.3502298
Unigene_761	chr8.S_165594803	3	118961656	0.3500679
Unigene_227	SYN24203	3	118961656	0.3495214
Unigene_537	PZE-103026200	3	118961656	0.3492307
Unigene_659	SYN12756	3	118961656	0.3487473
Unigene_913	chr5.S_71845163	3	122065637	0.3486838
Unigene_646	PZE-107022606	3	130000397	0.3480692
Unigene_164	PZE-103051373	3	130000397	0.3476934
Unigene_510	PZE-101136552	3	132703789	0.3459914
Unigene_917	SYN33029	3	138386278	0.3458333
Unigene_826	PZE-102121540	3	140857168	0.3453285
Unigene_381	PZE-110015612	3	144092738	0.3443851
Unigene_353	SYN16880	3	145272582	0.3438271
Unigene_506	chr4.S_18913586	3	145272582	0.3430368
Unigene_146	chr1.S_518354	3	146966814	0.3424341
Unigene_322	SYN34421	3	147199630	0.3401531
Unigene_618	SYN27300	3	147400304	0.3393225
Unigene_262	PZE-102135971	3	150588960	0.3388824
Unigene_521	PZE-109054414	3	155736726	0.3386668
Unigene_292	SYN24468	3	163077496	0.3386254
Unigene_408	SYN21717	3	163077496	0.33858
Unigene_1043	chr3.S_189472718	3	163077496	0.3380347
Unigene_470	PZE-102129186	3	163077496	0.3373852
Unigene_888	chr1.S_47810543	3	163077496	0.337311
Unigene_407	PZE-106041967	3	166691808	0.3364089
Unigene_746	SYN4382	3	168482460	0.3361092

Unigene_953	PZE-103083488	3	170112689	0.3359079
Unigene_681	SYN24468	3	173373439	0.3348412
Unigene_441	chr6.S_6807071	3	175705643	0.3346396
Unigene_445	PZE-103051373	3	177019165	0.3345225
Unigene_609	PZE-101188882	3	183262547	0.3343995
Unigene_834	PZE-103051373	3	186747886	0.3322222
Unigene_597	PZE-110041853	3	187444844	0.3319498
Unigene_448	chr2.S_158610701	3	189466771	0.330087
Unigene_271	PZE-103026200	3	189472718	0.328427
Unigene_866	chr9.S_134721246	3	191368421	0.3274279
Unigene_416	PZE-102092530	3	191997692	0.3260281
Unigene_73	PZE-108091013	3	196066316	0.32593
Unigene_497	PZE-103187403	3	196066316	0.322279
Unigene_31	PZE-109028012	3	196439095	0.3212942
Unigene_402	SYN6353	3	197019720	0.3212332
Unigene_171	PZE-110046499	3	197382561	0.3209396
Unigene_160	SYN32838	3	199243934	0.3198475
Unigene_598	PZE-103026200	3	200292283	0.3195238
Unigene_9	PZE-108044930	3	214200967	0.31927
Unigene_489	SYN24468	3	214280205	0.3192258
Unigene_106	chr6.S_1537453	3	217666097	0.3186795
Unigene_574	chr2.S_273615	3	223025711	0.3148108
Unigene_107	PZE-106015544	3	225587565	0.3139182
Unigene_564	chr1.S_119378679	3	229420316	0.3137253
Unigene_639	PZE-103069120	3	229637279	0.3136815
Unigene_341	PZE-103069120	3	232023437	0.3129274
Unigene_773	PZE-105093503	3	232023437	0.3109201
Unigene_935	PZE-107042706	4	30789	0.3103594
Unigene_309	PZE-103051373	4	4206268	0.3102501
Unigene_635	SYN11547	4	11466905	0.309248
Unigene_376	SYN11442	4	17150000	0.3091695
Unigene_955	chr5.S_203626294	4	17230867	0.3085426
Unigene_849	PZE-103072192	4	17230867	0.3082194
Unigene_559	PZE-103069120	4	17230867	0.3075712
Unigene_585	SYN21932	4	18371260	0.3074268
Unigene_686	PZE-107017955	4	18371260	0.3069262
Unigene_328	PZE-102083124	4	18371260	0.30625
Unigene_277	chr9.S_112941123	4	18371260	0.3054716
Unigene_163	PZE-103051373	4	18913586	0.3052888
Unigene_321	PZE-103141567	4	19604866	0.3050655
Unigene_434	PZE-103140256	4	21321109	0.3048558
Unigene_568	chr8.S_164437822	4	28921826	0.3035889
Unigene_527	PZE-102070673	4	32810595	0.3001721
Unigene_670	chr3.S_170112689	4	34267429	0.3
Unigene_627	PZE-102184087	4	45263706	0.2997845
Unigene_579	PZE-102145591	4	60357510	0.299369
Unigene_172	PZE-103027518	4	66773174	0.2992886
Unigene_603	PZE-102178513	4	67678951	0.2990255
Unigene_362	PZE-108073602	4	67945227	0.2989971

Unigene_457	chr3.S_187444844	4	80075419	0.2970034
Unigene_484	PZE-110043053	4	80075419	0.2967971
Unigene_173	PZE-110005768	4	80075419	0.2967032
Unigene_411	chr10.S_147354775	4	80901571	0.2961762
Unigene_412	PZE-101118313	4	80901571	0.2933742
Unigene_897	SYN37730	4	84084057	0.293012
Unigene_194	chr9.S_122827993	4	125816267	0.2922942
Unigene_734	PZE-102133503	4	130280780	0.2904642
Unigene_684	SYN24468	4	139474735	0.2901989
Unigene_365	chr5.S_2266351	4	143083152	0.2901753
Unigene_472	chr5.S_5198719	4	147915438	0.2901316
Unigene_10	chr8.S_57385616	4	154261101	0.2880995
Unigene_468	PZE-104018476	4	154261107	0.2866959
Unigene_867	chr1.S_290857647	4	159567215	0.2865797
Unigene_335	PZE-104075359	4	159567215	0.2865594
Unigene_253	PZE-107027367	4	159658242	0.286435
Unigene_582	chr3.S_5336296	4	178468003	0.2858529
Unigene_633	PZE-108036611	4	186596937	0.2853607
Unigene_203	chr1.S_19262211	4	192011682	0.2847748
Unigene_268	PZE-108135189	4	201365689	0.2838701
Unigene_161	PZE-103051373	4	218940992	0.2837304
Unigene_94	chr1.S_148457968	4	229539832	0.2830873
Unigene_538	PZE-110005768	4	238280592	0.282861
Unigene_281	PUT-163a-71422816-3214	4	239409883	0.2825015
Unigene_287	chr3.S_177019165	5	434525	0.2815567
Unigene_232	PZE-103078681	5	900450	0.2810088
Unigene_663	chr3.S_214280205	5	2266351	0.2809127
Unigene_429	PZE-109052401	5	5198719	0.2806366
Unigene_186	chr8.S_70994333	5	6329116	0.2804133
Unigene_379	PZE-103055069	5	6358033	0.2800679
Unigene_378	SYN10319	5	6913100	0.2792101
Unigene_358	chr7.S_164075793	5	7976971	0.2788839
Unigene_658	PZE-102092530	5	8331089	0.2777778
Unigene_653	PZE-101188454	5	8459893	0.276891
Unigene_456	PZE-107053869	5	11692339	0.2764912
Unigene_193	chr1.S_10998864	5	16322837	0.2764497
Unigene_252	PZE-107052204	5	16694261	0.2757173
Unigene_123	PZE-106043706	5	19895817	0.2746157
Unigene_435	chr5.S_23259479	5	22181198	0.2740441
Unigene_401	PZE-107052204	5	23259479	0.2732131
Unigene_22	PZE-102121540	5	24974805	0.2707516
Unigene_247	PZE-104017088	5	28116352	0.2704569
Unigene_514	SYN31995	5	46110053	0.2704168
Unigene_396	PZE-106094290	5	59311040	0.2689394
Unigene_150	PZE-110005768	5	60657192	0.2676307
Unigene_105	PZE-103026200	5	60799597	0.267039
Unigene_421	PZE-107052204	5	61235211	0.2667298
Unigene_181	SYN11547	5	67045207	0.2657731

Unigene_485	PZE-102129186	5	67045207	0.2650104
Unigene_233	PZE-103026172	5	67045207	0.264513
Unigene_345	PZE-103051373	5	67045207	0.2628061
Unigene_104	chr8.S_122410102	5	67045207	0.2591743
Unigene_729	SYN6397	5	71845163	0.2583942
Unigene_475	PZE-103069120	5	74919020	0.2581573
Unigene_443	PZE-110093225	5	76218943	0.2574647
Unigene_25	PZE-109067522	5	132171182	0.253822
Unigene_78	PZE-104017281	5	135456643	0.2537372
Unigene_196	chr8.S_164936982	5	140781215	0.2525091
Unigene_590	SYN7361	5	146729558	0.2513253
Unigene_215	PZE-101131640	5	147539836	0.2495007
Unigene_375	PZE-105147871	5	163412915	0.2494212
Unigene_438	chr6.S_152182424	5	166332322	0.2477846
Unigene_474	PZE-107045902	5	176115887	0.2464645
Unigene_437	chr8.S_171217312	5	190605873	0.2443899
Unigene_269	PZE-104019185	5	195305530	0.2443303
Unigene_561	PZE-105065508	5	200441907	0.2424242
Unigene_544	SYN2848	5	203626294	0.2421616
Unigene_243	PZE-110093110	5	204089470	0.2412659
Unigene_312	PZE-102092530	5	204181508	0.241066
Unigene_610	SYN487	5	208253030	0.2408175
Unigene_813	chr3.S_217666097	5	210105452	0.2404063
Unigene_77	SYN2435	5	211699086	0.2393808
Unigene_338	PZE-105061749	5	211699086	0.2392766
Unigene_480	PZE-103140256	5	211699086	0.2381075
Unigene_264	chr2.S_9014897	5	216738630	0.2363935
Unigene_371	PZE-106003021	6	1537453	0.2362267
Unigene_183	PZE-103164358	6	3987639	0.2313448
Unigene_191	PZE-104044882	6	6807071	0.2304458
Unigene_149	chr3.S_200292283	6	14580546	0.2300229
Unigene_307	chr6.S_96338585	6	18991506	0.2296069
Unigene_410	PZE-101084367	6	25823165	0.2295303
Unigene_285	PZE-105041910	6	29127386	0.2289706
Unigene_282	PZE-102111568	6	31871597	0.2288583
Unigene_241	PZE-110093110	6	33792437	0.2277127
Unigene_302	chr4.S_192011682	6	37081828	0.2274074
Unigene_404	PZE-102129186	6	40622939	0.2266484
Unigene_507	chr5.S_6913100	6	48548810	0.2265098
Unigene_26	PZE-107053869	6	63699585	0.2237429
Unigene_439	PZE-103069120	6	67258537	0.2235811
Unigene_426	PZE-102140115	6	87408682	0.2234776
Unigene_299	PZE-104051267	6	91617959	0.2233899
Unigene_557	PZE-110005768	6	91617959	0.2227478
Unigene_111	chr10.S_82208405	6	91617959	0.2225312
Unigene_403	chr2.S_3389929	6	93443995	0.2210987
Unigene_567	chr10.S_139858512	6	93443995	0.2195545
Unigene_290	PZE-103051373	6	93443995	0.2191743
Unigene_54	PZE-108038204	6	96338585	0.2179077

Unigene_129	PZE-101134574	6	98073154	0.2177994
Unigene_165	chr5.S_11692339	6	112906956	0.217508
Unigene_274	PZE-107053869	6	113108685	0.2172472
Unigene_318	chr6.S_144724241	6	117014189	0.2169085
Unigene_617	chr4.S_45263706	6	138251479	0.2166211
Unigene_245	SYN28781	6	144724241	0.2153739
Unigene_90	SYN19282	6	149598449	0.2151027
Unigene_346	PZE-107052204	6	149598449	0.2144816
Unigene_115	PZE-102112650	6	149598449	0.2140526
Unigene_334	chr8.S_151657559	6	149598449	0.2135372
Unigene_66	SYN2764	6	149598449	0.2129887
Unigene_337	chr4.S_229539832	6	150738392	0.2127758
Unigene_214	chr5.S_16694261	6	152182424	0.2117236
Unigene_467	PZE-102121540	6	154131008	0.209993
Unigene_651	PZE-101209179	6	154131008	0.2094527
Unigene_303	PZE-106094290	6	154131008	0.2090746
Unigene_614	PZE-105166788	6	154131008	0.2081229
Unigene_339	PZE-102113792	6	154131008	0.2075913
Unigene_236	chr5.S_59311040	6	154131008	0.20728
Unigene_189	PZE-103051373	6	156378934	0.2064821
Unigene_50	chr1.S_213153061	6	163404705	0.2052417
Unigene_344	chr4.S_238280592	6	167267291	0.2049762
Unigene_258	PZE-103069120	7	1267668	0.2046861
Unigene_276	PZE-104017281	7	4902630	0.2044443
Unigene_231	ZM013153-0344	7	4902820	0.204249
Unigene_370	PZE-103072192	7	12622282	0.2038711
Unigene_419	SYN825	7	13616012	0.2029672
Unigene_400	PZE-105018063	7	15455944	0.2005565
Unigene_175	SYN21804	7	19347165	0.1997841
Unigene_266	PZE-102112078	7	22630180	0.1996168
Unigene_531	SYN19523	7	24795393	0.1992281
Unigene_234	chr2.S_177183870	7	25797408	0.1992203
Unigene_224	PZE-103055573	7	31914200	0.1988544
Unigene_532	chr3.S_15040324	7	33171629	0.1980391
Unigene_75	PZE-101136965	7	33226812	0.1976242
Unigene_49	PZE-106028261	7	41464276	0.1956169
Unigene_67	PZE-106062004	7	84175305	0.1953237
Unigene_144	PZE-103051373	7	94019794	0.1951247
Unigene_225	PUT-163a-30704694-1894	7	94607102	0.1928803
Unigene_388	PZE-107047532	7	97346665	0.1925626
Unigene_187	PZE-103069120	7	97619749	0.1918578
Unigene_280	chr6.S_150738392	7	97619749	0.1914156
Unigene_170	chr6.S_98073154	7	102392865	0.1907616
Unigene_206	SYN11547	7	102392865	0.1904201
Unigene_7	chr3.S_146966814	7	102392865	0.1894988
Unigene_208	PZE-102092530	7	102392865	0.1893228
Unigene_516	chr9.S_148445708	7	102392865	0.1884586
Unigene_18	chr3.S_173373439	7	104884259	0.1880553

Unigene_128	SYN21437	7	105235123	0.1846733
Unigene_325	PZE-104134641	7	105235123	0.1845447
Unigene_415	PZE-101241530	7	105235123	0.1844668
Unigene_34	chr7.S_161684618	7	105235123	0.1843024
Unigene_313	PZE-102092530	7	108534830	0.1839631
Unigene_117	PZE-101188882	7	108534830	0.1837102
Unigene_398	PZE-103051373	7	108534830	0.1832876
Unigene_330	PZE-102092530	7	124212468	0.1818572
Unigene_89	SYN3060	7	127270683	0.1808211
Unigene_91	PZE-102092530	7	130248774	0.1798088
Unigene_228	chr1.S_252257234	7	130784233	0.1793912
Unigene_500	PZE-103078681	7	134972234	0.1786988
Unigene_126	PZE-101086807	7	143693729	0.1785604
Unigene_156	PZE-103052172	7	147162383	0.1777258
Unigene_442	chr1.S_36221209	7	154254921	0.17766
Unigene_329	PZE-105000613	7	154254921	0.1771371
Unigene_440	chr10.S_1499134	7	157359206	0.1767172
Unigene_139	chr7.S_4902820	7	157572103	0.1763982
Unigene_190	PZE-103051373	7	161684618	0.1754804
Unigene_204	PZE-102092530	7	161700825	0.1752692
Unigene_466	chr2.S_36835546	7	164075793	0.1747235
Unigene_349	ZM013153-0344	7	171700452	0.1734214
Unigene_120	PZE-103088828	7	172472359	0.173065
Unigene_222	SYN15191	8	840333	0.171958
Unigene_33	PUT-163a-89249846-4643	8	6396539	0.1696715
Unigene_211	SYN17537	8	6661546	0.1694721
Unigene_332	PZE-103026200	8	10071820	0.169035
Unigene_423	PZE-106012443	8	12831230	0.16843
Unigene_135	PZE-102092530	8	14753943	0.1683777
Unigene_37	PZE-107024153	8	14952326	0.1682218
Unigene_38	chr9.S_146963566	8	17303969	0.1680396
Unigene_40	SYN11547	8	31313144	0.165894
Unigene_278	chr1.S_220375892	8	34734112	0.1646851
Unigene_133	PZE-108104216	8	34734112	0.1635328
Unigene_103	PZE-103020025	8	37679164	0.1635171
Unigene_80	PZE-103051373	8	56662602	0.1633983
Unigene_88	PZE-103095814	8	57385616	0.1629533
Unigene_246	PZE-102135971	8	62285587	0.1623238
Unigene_134	PZE-105099081	8	70994333	0.1620453
Unigene_273	PZE-105071436	8	72932205	0.1618757
Unigene_24	PZE-103102545	8	74681765	0.161489
Unigene_113	PZE-101241535	8	78832554	0.1612476
Unigene_176	PZE-102092530	8	93924012	0.160851
Unigene_217	PZE-105065508	8	102537422	0.160247
Unigene_493	PZE-104005779	8	111626288	0.1601597
Unigene_458	SYN21932	8	115837944	0.1597136
Unigene_205	PZE-104051018	8	121896035	0.1591335
Unigene_382	PZE-104042707	8	122410102	0.1589397

Unigene_122	chr7.S_157359206	8	128555883	0.1589378
Unigene_348	PZE-108091013	8	140749569	0.1574728
Unigene_71	PZE-101188882	8	141103993	0.1573913
Unigene_98	PZE-103051373	8	148139960	0.1567557
Unigene_324	PZE-103051373	8	148139960	0.1564469
Unigene_200	PZE-102129186	8	148139960	0.1561073
Unigene_288	SYN21577	8	148139960	0.1532835
Unigene_261	chr7.S_147162383	8	151186202	0.152716
Unigene_594	PZE-103080084	8	151657559	0.1524688
Unigene_460	chr6.S_113108685	8	154470213	0.1520321
Unigene_17	SYN11547	8	158895597	0.1513361
Unigene_23	PZE-103069120	8	159449928	0.151076
Unigene_64	PZE-104124043	8	159674303	0.1502581
Unigene_132	chr1.S_194663220	8	161560312	0.1488875
Unigene_47	PZE-103069120	8	161560312	0.1486716
Unigene_238	chr3.S_122065637	8	164437822	0.1486701
Unigene_529	SYN3946	8	164936982	0.1483989
Unigene_142	PZE-110005768	8	165594803	0.1473516
Unigene_414	PZE-107052204	8	170434394	0.1471001
Unigene_92	SYN10319	8	171217312	0.1468057
Unigene_137	PZE-108012465	8	174610752	0.1463648
Unigene_220	SYN22812	9	8417637	0.1454888
Unigene_43	PZE-102121540	9	29402811	0.145336
Unigene_53	chr7.S_1267668	9	47507315	0.1446705
Unigene_30	PZE-110015612	9	49188095	0.1439148
Unigene_374	PZE-102169349	9	55869093	0.1432191
Unigene_289	chr6.S_48548810	9	90376842	0.1424634
Unigene_44	PZE-102184087	9	93447327	0.1407828
Unigene_35	PZE-102091469	9	93812227	0.1407354
Unigene_355	SYN3945	9	93812227	0.140606
Unigene_152	SYN25067	9	94183585	0.1400257
Unigene_406	PZE-101246842	9	96775630	0.1399253
Unigene_177	PZE-106094290	9	103683027	0.1389471
Unigene_185	SYN15586	9	109847420	0.1389046
Unigene_310	PZE-102111568	9	111665960	0.1381464
Unigene_99	PZE-103066271	9	112941123	0.1376297
Unigene_51	chr1.S_269299215	9	115056355	0.1375674
Unigene_263	PZE-105039807	9	120803982	0.1366998
Unigene_101	PZE-102153851	9	120878022	0.1360628
Unigene_192	SYN11547	9	122827993	0.1353697
Unigene_494	PZE-102044503	9	124031518	0.1352376
Unigene_79	PZE-107015559	9	124031518	0.1345268
Unigene_283	PZE-102121540	9	124031518	0.1336534
Unigene_221	SYN4382	9	128648849	0.1326146
Unigene_55	PZE-109080594	9	131375711	0.1318513
Unigene_52	PZE-103051373	9	134721246	0.1315761
Unigene_207	SYN825	9	142467499	0.1314295
Unigene_83	chr7.S_161700825	9	146963566	0.1311405
Unigene_84	PZE-102092530	9	148445708	0.1311103

Unigene_342	chr5.S_6329116	9	149015650	0.1307381
Unigene_87	PZE-105065508	9	151999544	0.1303501
Unigene_119	chr1.S_9435531	10	1499134	0.1301605
Unigene_42	PZE-103051373	10	4737187	0.1298583
Unigene_237	chr2.S_19265678	10	4737187	0.1296097
Unigene_320	chr4.S_186596937	10	4737187	0.1295343
Unigene_251	PZE-107074363	10	4737187	0.1291533
Unigene_279	SYN3887	10	4737187	0.1287627
Unigene_61	PZE-106013164	10	4991331	0.1285483
Unigene_112	PZE-110024674	10	11195463	0.128519
Unigene_131	PZE-103001421	10	15407921	0.1283676
Unigene_65	PZE-103051373	10	15407921	0.1275272
Unigene_96	SYN31979	10	18399591	0.1273702
Unigene_199	PZE-102129186	10	18674222	0.1269398
Unigene_240	chr5.S_8459893	10	54368281	0.1258778
Unigene_102	PZE-101101256	10	63065942	0.1255186
Unigene_235	chr1.S_296985852	10	80039769	0.1243271
Unigene_260	PZE-102129186	10	81750216	0.1209412
Unigene_216	SYN11547	10	82104698	0.1208391
Unigene_259	chr8.S_154470213	10	82206101	0.1208351
Unigene_286	chr5.S_6358033	10	82208405	0.1205157
Unigene_14	SYN21437	10	83457291	0.1197589
Unigene_121	chr4.S_21321109	10	83457291	0.1187955
Unigene_148	SYN487	10	83457291	0.1187301
Unigene_167	chr4.S_178468003	10	83457291	0.1182051
Unigene_86	chr9.S_149015650	10	83457291	0.1172959
Unigene_57	chr3.S_229637279	10	83457291	0.1171817
Unigene_56	PZE-101120516	10	83457291	0.1170531
Unigene_154	chr7.S_143693729	10	83457291	0.1170287
Unigene_82	PZE-103051373	10	83457291	0.1157212
Unigene_174	chr3.S_229420316	10	83457291	0.1141845
Unigene_244	PZE-103072192	10	87270271	0.1128187
Unigene_60	PZE-109038031	10	105877918	0.1114043
Unigene_110	SYN11547	10	124237373	0.1111225
Unigene_3	PZE-102129186	10	124299289	0.1079246
Unigene_311	PZE-103051373	10	126870772	0.1069623
Unigene_48	ZM013153-0344	10	129462987	0.1061109
Unigene_147	PZE-106094290	10	130142821	0.1058462
Unigene_166	PZE-102092530	10	139858512	0.1054475
Unigene_195	PZE-104000023	10	141264076	0.1052337
Unigene_230	PZE-103051373	10	141264076	0.104858
Unigene_85	SYN8447	10	141274474	0.1041759
Unigene_39	SYN13863	10	147354775	0.1029457
Unigene_179	PZE-103069120	10	147960881	0.1029377
Unigene_15	PZE-107027923	10	147960881	0.1028202
